# Monomer centred selectivity guidelines for sulfurated ring-opening copolymerisations†

**DOI:** 10.1039/d4sc05858e

**Published:** 2024-10-24

**Authors:** Merlin R. Stühler, Marie Kreische, Christoph Fornacon-Wood, Susanne M. Rupf, Robert Langer, Alex J. Plajer

**Affiliations:** a Makromolekulare Chemie, Universität Bayreuth Universitätsstraße 30 95447 Bayreuth Germany alex.plajer@uni-bayreuth.de; b Intitut für Chemie und Biochemie, Freie Universität Berlin Fabeckstraße 34-36 14195 Berlin Germany; c Institute for Chemistry, Martin-Luther-University Halle-Wittenberg Kurt-Mothes-Str. 2 06120 Halle Germany; d Bayrisches Polymer Institut (BPI), Universität Bayreuth Universitätsstraße 30 95447 Bayreuth Germany

## Abstract

Sulfur-containing polymers, such as thioesters and thiocarbonates, offer sustainability advantages, including enhanced degradability and chemical recyclability. However, their synthesis remains underdeveloped compared to that of their oxygen-containing counterparts. Although catalytic ring-opening copolymerization (ROCOP) can provide access to sulfur-containing polymers, these materials often exhibit uncontrolled microstructures and unpredictable properties. A comprehensive model that elucidates the factors determining selectivity in these catalytic reactions is still lacking, despite its central importance for advancing these polymerizations into widely applicable methodologies. In this study, we investigate the factors that lead to selectivity in sulfurated ROCOP across various monomer combinations, including thioanhydrides or carbon disulfide with epoxides, thiiranes, and oxetanes. We find that unwanted by-products primarily arise from backbiting reactions of catalyst-bound alkoxide chain ends, which can be mitigated by (i) selecting monomers that form primary alkoxide of thiolate chain ends, (ii) maximizing ring strain in the backbiting step, and (iii) timely termination of the polymerization. By applying these strategies, the selectivity of the catalytic ROCOP can be controlled and we successfully synthesized perfectly alternating poly(esters-*alt*-thioesters) from various oxetanes and the highly industrially relevant ethylene oxide. Our study thereby shifts the focus for achieving selectivity from catalyst to monomer choice providing valuable mechanistic insights for the development of future selective polymerizations, paving the way for sulfurated polymers as potential alternatives to current commodity materials.

## Abstract 2

Factors determining sequence selectivity in sulfur-containing ring-opening copolymerization are governed by the types of chain ends generated so that monomers which lead to primary alkoxide and thiolate chain ends, are particularly suitable.

## Graphical abstract

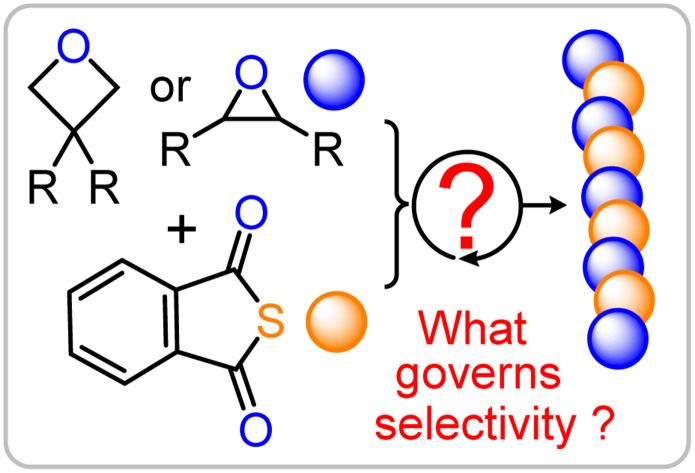

## Introduction

**Fig. 1:**
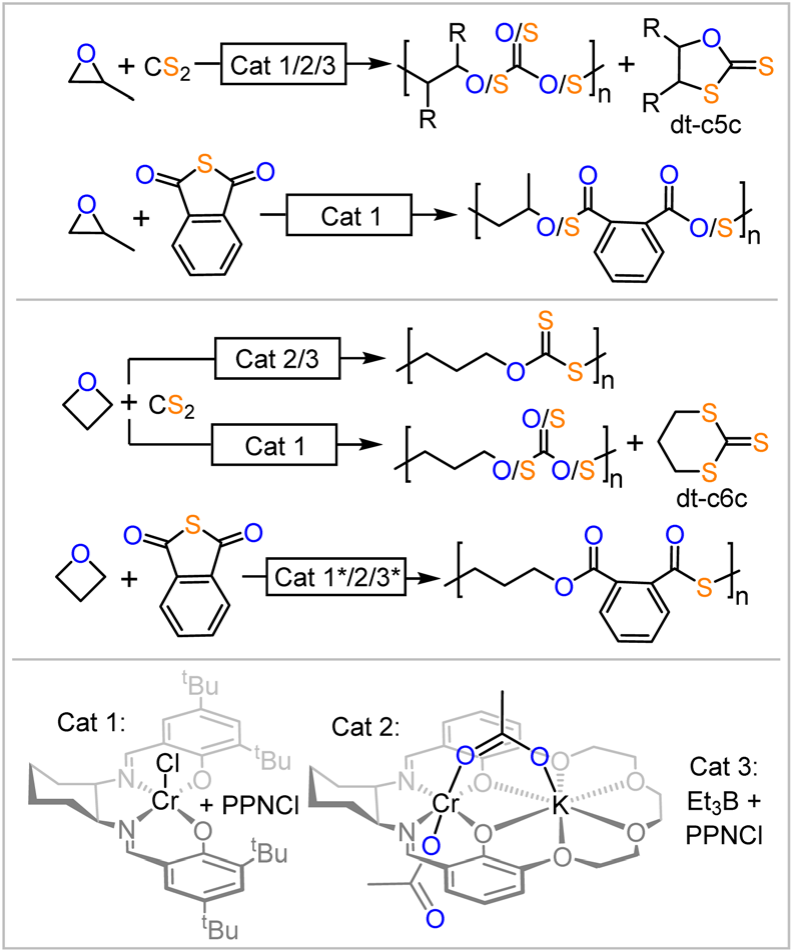
Ring-opening copolymerisations (ROCOP) producing sulfur containing polymers. * New data recorded in this report as part of introductory studies.

Ring-opening copolymerisation (ROCOP) of a strained heterocycle with a heteroallene or cyclic anhydrides gives access to a large variety of polymer microstructures that would not be easily accessible otherwise.^1^ Having gained prominence as a route to access a wide range of polyester from the ROCOP of cyclic anhydride with epoxides or polycarbonate from the ROCOP of CO_2_ with epoxides, this methodology recently emerged to access sulfur containing polymers such as polythiocarbonates and polythioesters.^2–4^ Such polymers have gained increasing interest due to the circumstance that sulfur containing polymers can feature improved degrees of semi-crystallinity, depolymerisability and enhanced optical properties compared to their all-oxygen analogues as well as unique properties such as the ability to coordinate transition metals.^5–24^ In this respect, we have demonstrated that materials selectively degrade at the sulfur-containing link or depolymerise more effectively than their all oxygen analogues.^25,26^ Unfortunately, ROCOPs of sulfurated substrates are often plagued by side reactions that lead to disordered polymer microstructures. The degree of disorder is highly dependent on the specific reaction conditions, which negatively impacts the thermal and mechanical properties, such as the loss of semi-crystallinity due to increased disorder. Additionally, the extent of this disorder can be highly irreproducible between laboratories, further limiting the utility and real-world impact of these materials. Specifically in the ROCOP of phthalic thioanhydride (PTA) and epoxides, one would for example expect the formation of poly(ester-*alt*-thioester) from alternating insertion of the two monomers, yet diester as well as dithioester links are likewise often observed to form (Fig. 1).^27–31^ Due to its industrial relevance propylene oxide is most commonly employed here as the epoxide. Likewise, although one would expect that, for example, CS_2_/epoxide ROCOP yields polydithiocarbonates featuring –O–C(=S)–S– (OSS) links from alternating insertion of the monomers, a distribution of different thiocarbonate links is obtained for all epoxides investigated so far. This process scrambling the oxygen and sulfur centres in the polymer chain has been termed O/S scrambling and is also observed in many other sulfurated ROCOPs.^32–46^ Curiously, the ROCOP of carbonyl sulfide (COS) is not affected by this process delivering alternating copolymers.^47^

Conflicting hypotheses have been formulated leading to O/S scrambling involving intermolecular attack of a random polymer link by alkoxide chain ends, intramolecular attack of alkoxide chain-ends into adjacent polymer links as well as the intermediate formation of small-molecule intermediates.^27,44,48^ Most importantly though none of these hypotheses explain when to expect and how to avoid O/S scrambling. Developing such guidelines, however, is of central importance for advancing these polymerisations into useful and widely applicable methodologies.

Considering the combined literature when scrambling is most pronounced, however, suggest that such rules exist. All of the most performant catalyst classes commonly employed in ROCOP (bicomponent metal catalyst Cat. 1, bimetallic metal catalyst Cat. 2 and bicomponent borane catalyst Cat. 3 in Fig. 1) are unselective in CS_2_/epoxide ROCOP with all epoxides investigated so far. In PTA/epoxide ROCOP, bicomponent catalysis with either Cat. 1 or Cat. 3 produces linkage-scrambled products with propylene oxide; heterobimetallic catalysis (*i.e.*, Cat. 2) remains to be explored. When moving from epoxides to oxetane, the four membered analogue of ethylene oxide, PTA/Oxetane catalysed by Cat. 3 results in perfectly alternating ROCOP, while both Cat. 2 and Cat. 3 catalyse CS_2_/Oxetane ROCOP near perfectly; Cat. 1 yields scrambled products.^25,26,48,49^ Taken together, these results lead us to hypothesize that moving from epoxides to oxetane may result in a catalyst-independent improvement in selectivity. This suggests that certain substrates are intrinsically more suited for sulfurated ROCOP, resulting in high selectivity regardless of the catalyst employed. However, this remains to be understood, as it is unclear if or how the nature of the propagating chain and the differences in associated ring-strain energies when moving to four-membered rings influence selectivity. Similar observations were made for elementary steps in catalytic reactions, such as cross coupling reactions, where the nature of the substrate mostly determines the dominating reaction pathway.^50,51^

In order to confirm this notion, we conducted introductory experiments (see ESI Section S2†) leading into the current study (Fig. 1 highlighted with *) as some common catalysts remained to be explored in the copolymerisation of PTA and oxetane. Hence, PTA/oxetane ROCOP was investigated with Cat. 1 and Cat. 3. These runs were conducted at 80 °C and with a loading of 1 eq. Cat.: 1000 eq. PTA: 1000 eq. OX to also allow for comparison of these results with literature-known copolymerisations and those conducted later in this study.^26^ Cat. 1 results in 20% turnover after 45 min producing a polymer with *M*_n_ = 24.5 kDa (*Đ* = 1.2) and Cat. 3 results in 31% turnover after 7 h producing a polymer with *M*_n_ = 45.9 kDa (*Đ* = 1.3). Both polymers are perfectly alternating poly(esters-*alt*-thioesters) with no signs of scrambled links. This observation confirms our hypothesis that oxetane is intrinsically robust to O/S scrambling side reactions with PTA, as the same catalysts result in scrambled polymers with propylene oxide.

Motivated by the notion that the nature of the monomer, rather than the choice of catalyst, appears to determine ROCOP selectivity, we then proceeded to conduct a combined experimental and computational study on various ROCOPs involving epoxides, oxetanes, PTA and CS_2_ with Cat. 2 as a catalyst to understand how selectivity depends on monomer choice. We chose LCrK to proceed with, as multimetallic catalysts have been shown to function *via* well-defined mechanisms in which each metal takes on a distinct role, making these particularly suited for computational investigations.

## Results and discussion

### Quantifying substituent effects in O/S scrambling

**Fig. 2:**
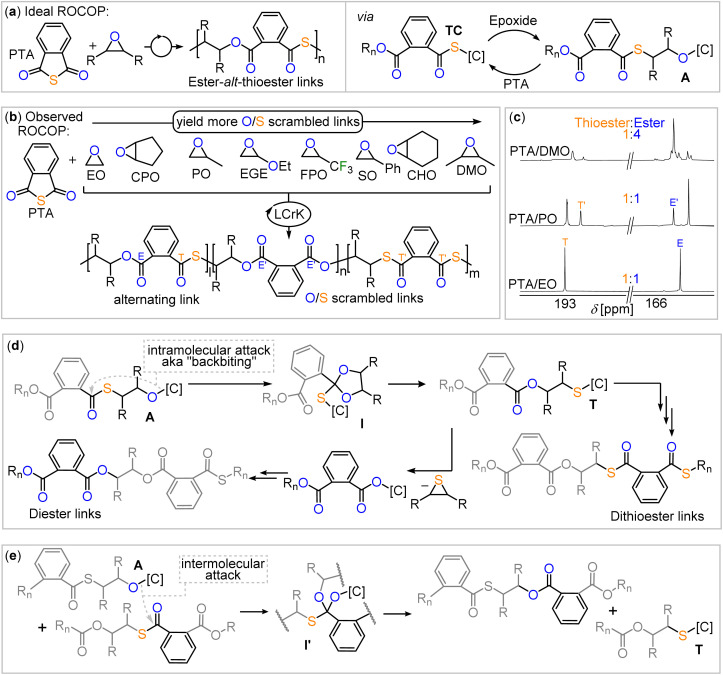
(a) Alternating PTA/epoxide propagation, leading to ester-*alt*-thioester links. (b) PTA/epoxide scope explored in this study and observed scrambling behaviour. (c) Comparison of the carbonyl region of the ^13^C NMR spectrum (CDCl_3_, 126 MHz) of the copolymers obtained from PTA/DMO, PTA/PO and PTA/EO ROCOP. (d) O/S scrambling pathways producing dithioester and diester links started by an intramolecular attack of an alkoxide intermediate into a thioester link and (e) intermolecular variant. [C] denotes catalyst, *R*_n_ denotes polymer chain.

As many epoxides are commercially available, we hypothesised that their exploration in copolymerisations with PTA employing Cat. 2 as a catalyst could shed light on the scrambling process. As shown in Fig. 2(a), PTA/epoxide ROCOP leads in first instance to alternating ester-*alt*-thioester motifs, while the O/S scrambling reactions give rise to diester as well as dithioester links. Consequently, the quaternary carbonyl region (150–200 ppm) of the ^13^C NMR spectrum shows two signals (Fig. 2(c)) if alternating ROCOP occurs and additional signals if side reactions occur. Therefore, we used this region, and in particular integration of the respective signals, as an experimental handle to obtain insight into the polymer microstructure and scrambling process. To ensure quantitative interpretability of the ^13^C NMR data, we determined the spin-lattice relaxation times of each of the signals (*T*_1_ = 1.29–1.51 s, see Table S1†) and chose scan times of 2 s, at which all full relaxation occurred for each of the resonances. This allows for the analysis of the microstructure by relative integration of the ^13^C resonances to quantify the precise amount of scrambling that occurs in the copolymerisation reaction.

Employing LCrK (Cat. 2) in PTA/epoxide ROCOP at 80 °C and a loading of 1 eq. Cat. 2: 1000 eq. epoxide: 1000 eq. PTA, results in quantitative polymer formation (*M*_n_ = 6.7–40.8 kg mol^−1^, *Đ* = 1.3–1.6) with a starkly variable degree of O/S scrambling summarised in Table 1 and ESI Section S3.† Moving from ethylene oxide (EO, run #1) to the mono- (PO, run #2) and to the 1,2-dimethylsubstituted (DMO, run #3) derivative, leads to a progressing increase of scrambling from 100% alternating ester-*alt*-thioester links for EO to 67% for PO to 28% for DMO. Not only does the percentage of alternating ester-*alt*-thioester links in the polymer decrease, but the proportion of overall ester to thioester links shift from a 1 : 1 for PTA/EO and PTA/PO ROCOP to a 1 : 4 ratio for PTA/DMO ROCOP. Clearly, deviation from the 1 : 1 ratio is a further measure, to which degree scrambling occurs. The number of substituents is more important than their nature, as the monosubstituted epoxide SO, EGE and FPO (runs #6, #7, #8) featuring substituents with electron-rich and poor substituents of different steric demands show only slight deviations from the 1 : 1 ester : thioester ratio, which is substantially more selective than for DMO. Furthermore, for SO and TFPO, stereoselectivity is lost, preventing identification of the proportion of alternating ester-*alt*-thioester links. Remaining at 1,2 disubstituted but now alicyclic epoxides, CHO (run #4) and CPO (run #5) an increase in selectivity compared to DMO is observed. CHO leads to a 1.4 : 1 ester : thioester ratio in 56% ester-*alt*-thioester selectivity and CPO yields a copolymer with a 1 : 1 ester : thioester ratio >95% ester-*alt*-thioester selectivity.‡‡We previously reported the ROCOP of 3,3′-substituted oxetanes with CS_2_ employing Cat. 2 under comparable conditions, which generally resulted in some O/S scrambling.^25^ Comparatively, this suggests that CS_2_ ROCOP is more prone to scrambling than PTA ROCOP.

**Table 1:** LCrK catalysed (Cat 2) PTA/epoxide ROCOP^a^

Run	Epoxide	*t* [h]	Conversion^b^ [%]	Alternation^c^ [%]	Ester : thioester^d^	*M*_n,app_ e [kDa], (*Đ*)
#0^g^	OX	2.3	99	100	1 : 1	52.1 (1.4)
#1	EO	27	94	100	1 : 1	33.0 (1.4)
#2	PO	2	68	67	1 : 1	17.1 (1.4)
#3	DMO	180	55	28	1 : 4	6.9 (1.4)
#4	CHO	38	26	56	1 : 1.3	6.7 (1.4)
#5	CPO	38	41	95	1 : 1	10.7 (1.4)
#6	SO	17	24	n.d.^f^	1 : 1.2	8.4 (1.5)
#7	EGE	9	70	61	1 : 1.1	11.0 (1.3)
#8	FPO	0.1	80	n.d.^f^	1 : 1.2	40.8 (1.6)

a ROCOP conducted at 80 °C with 1 eq. LCrK (cat. 2): 1000 eq. epoxide: 1000 eq. PTA. *T* = 80 °C.

b PTA conversion calculated by comparing the relative integrals in the normalised ^1^H NMR spectrum (CDCl_3_, 400 MHz) of aromatic resonances due to polymer *versus* unconsumed PTA.

c Relative integrals in the ^13^C NMR spectrum (CDCl_3_, 126 MHz) from carbonyl resonances due to alternating ester-*alt*-thioester links compared to other carbonyl resonances.

d Relative ratio of the integrals in the ^13^C NMR spectrum (CDCl_3_, 126 MHz) from ester to thioester carbonyl resonances.

e Determined by GPC (gel permeation chromatography) measurements conducted in THF, using narrow MW polystyrene standards to calibrate the instrument.

f Not determined due to complexity of the spectrum from regio-unselective epoxide ring-opening.

g Reported in our previous work ref. 26.

It should be noted that no clear correlation between conversion and reached molecular mass could be established and hence, these result must not be overinterpreted. We attribute this to varying amounts of protic impurities in the different epoxide monomers, potentially remaining even after purification.^2^

### Intra- *versus* intermolecular scrambling

Considering the hypothesis outlined in the introduction, that linkage selectivity might be tied to comonomer choice, we wondered whether the PTA/epoxide selectivity observed in Table 1 can also be understood in this context. Looking at previous investigations,^27,44,48^ side reactions in which catalyst bound alkoxide chain-ends A react with thioester links, rather than with PTA in the case of selective propagation, forming an ester link and a catalyst bound thiolate T are essential the O/S scrambling reaction (see Fig. 2(d) and (e)). This series of steps effectively exchanges the oxygen atom at the alkoxide chain end with a sulfur atom of a thioester link, and it is termed O/S exchange. Yet, in order to answer the question under which circumstances O/S scrambling is likely to arise, it is pertinent to understand whether this O/S exchange occurs in an intramolecular fashion at the propagating chain end, also known as backbiting (Fig. 2(d)), or between a propagating chain end and a random link (Fig. 2(e)) in an intermolecular fashion. With respect to the deviation from the 1 : 1 ester : thioester ratio, this can be rationalised as shown in Fig. 2(d). Here, a secondary cycle occurs in which catalyst bound thiolate chain ends T eliminate thiiranes to form catalyst bound carboxylate chain ends, which then propagate. However the key O/S exchange step must occur first.^44^

**Fig. 3:**
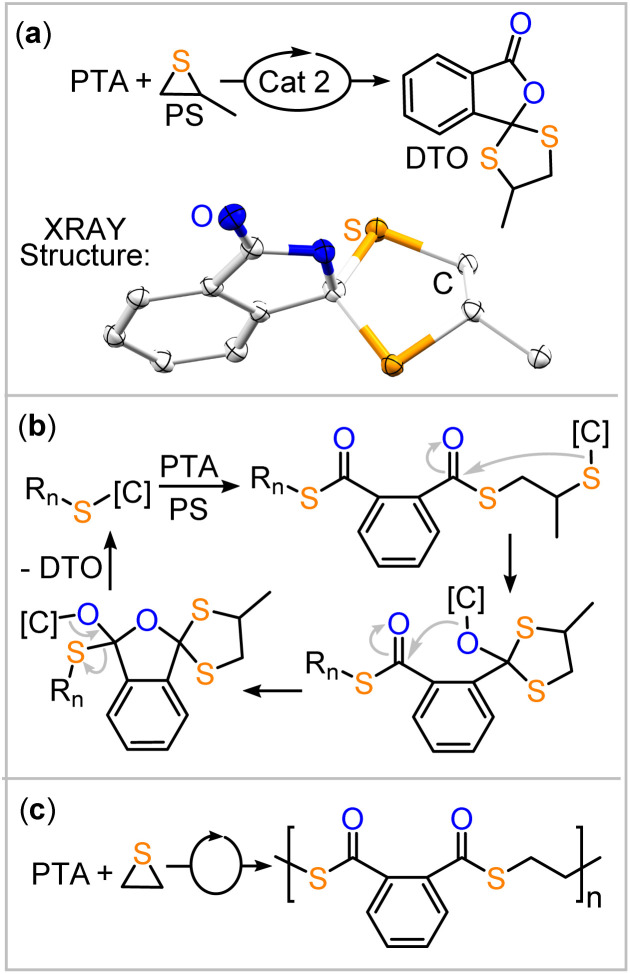
(a) PTA/PS coupling resulting in dithio-orthoester (DTO) formation; conditions analogous to Table 1. (b) Proposed mechanism of formation. (c) PTA/ES ROCOP resulting in poly(thioester) formation.

Direct evidence for the existence of the backbiting pathway (Fig. 2(d) in the ROCOP of PTA was obtained by its ROCOP with propylene sulfide (PS), the sulfur homologue of PO (ESI Section S4†). In contrast to the ROCOP of PTA/PO, the combination of PTA and PS results in rapid solidification of the reaction mixture, forming a crystalline solid. No polymeric product could be identified to form by GPC analysis. Single crystal XRD analysis of the product reveals formation of a cyclic dithio orthoanhydride as shown in Fig. 3(a). Its formation can be rationalised by a backbiting pathway outlined in Fig. 3(b) forming a cyclic intermediate which continues to react under elimination of a catalyst bound thiolate and generation of the spirocyclic product. In fact, the spirocycle represents a trapped form of intermediate formed by backbiting. These findings provide direct crystallographic evidence for a pathway involving backbiting of thiolate chain-ends to adjacent thioester links, as depicted in Fig. 2(d). With the unsubstituted variant of propylene sulfide, ethylene sulfide (ES), this pathway is disfavoured and polymer formation is observed rather than exclusive small molecule formation. PTA/ethylene sulfide (ES) ROCOP yields polythioester in 60% yield (*M*_n_ = 8.7 kDa, *Đ* = 1.7) under conditions analogous to Table 1.

**Fig. 4:**
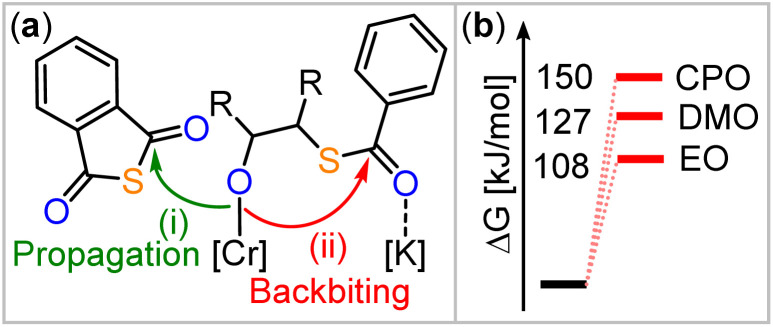
(a) Model alkoxide intermediate comparing intermolecular propagation with PTA in pathway (i) with backbiting in pathway (ii). R = H (for EO), Me (for DMO), –C_3_H_6_– (for CPO). (b) Comparison of backbiting energies in pathway (b).

Consequently, we focused our attention to the backbiting pathway shown in Fig. 2(d), which involves formation of a cyclic intermediate I, to rationalise the observed O/S scrambling listed in Table 1. Hence, for the formation of I, substituent effects must be considered in light of their tendency to induce cyclisation *via* backbiting. Therefore, we turned to quantum chemical investigations, using density functional theory (DFT) on B97D3/def2-TZVPP level of theory to initially investigate backbiting by alkoxides sitting adjacent to a thioester link (see Fig. 4 and ESI Section S6†). We focused on intermediates from the ROCOP of EO, CPO and DMO with PTA as these monomer combinations yield the least and most heavily scrambled polymers respectively. The assumed structure of the alkoxide intermediate in which the chain end is coordinated in proximity to the incoming PTA monomer was chosen in reference to related heteroallene copolymerisations of bimetallic catalysts.^44^

The transition state for backbiting (pathway (ii) in Fig. 4) was calculated to be significantly higher in Gibbs energy (Δ*G*^‡^ = +149.7 kJ mol^−1^) for CPO than for DMO (Δ*G*^‡^ = +126.6 kJ mol^−1^) and EO (Δ*G*^‡^ = +108.1 kJ mol^−1^). The high barrier for back biting in case of CPO is in line with a small degree of scrambling, whereas the methyl-substituted oxirane DMO gives rise to a smaller barrier and a high degree of scrambling. The high Gibbs energy of the transition state for CPO can be understood due to the formation of a bicyclic intermediate I, meaning that increasing strain in the backbiting step is a strategy to reduce O/S scrambling. This also provides a potential explanation why DMO results in more scrambling than monosubstituted epoxides, as backbiting for DMO could be more favoured by angular compression effects.^52^

However, as EO has the lowest barrier for backbiting, but yields perfectly alternating polymer with PTA, another effect must be considered. When comparing the thermodynamics of propagation, *i.e.* PTA insertion, *versus* backbiting for all studied epoxides (EO, DMO and CPO), all reaction steps of the model alkoxide were found to be uphill in Gibbs energy, indicating that after an initial and exergonic epoxide opening the resulting alkoxide intermediate may be a resting state. In line with the absence of scrambling in case of EO, the ring-opening of PTA in pathway (i) is thermodynamically favoured for EO (ΔΔ*G* = −9.7 kJ mol^−1^) with respect to the backbiting in pathway (ii), whereas for DMO (ΔΔ*G* = +4.5 kJ mol^−1^) and CPO (ΔΔ*G* = +9.8 kJ mol^−1^) the backbiting pathway (ii) is thermodynamically more favourable. We infer that this is a consequence of the fact that EO ring-opening forms primary alkoxide chain ends rather than secondary alkoxide chain as is the case for DMO and CPO (as well as for monosubstituted epoxide after ring-opening at the CH_2_ position), favouring propagation thermodynamically *versus* backbiting. Hence, a decrease in steric hindrance at the alkoxide chain end appears to favor nucleophilic attack during propagation more than it affects the one associated with backbiting.

### Weighing selectivity determining factors

**Fig. 5:**
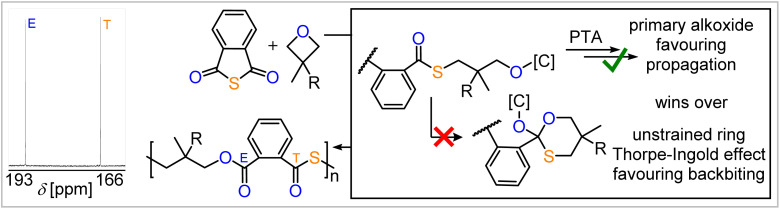
Controlled PTA ROCOP with various 3,3′-geminally substituted oxetanes, revealing the importance of primary alkoxide formation. Zoom into the ^13^C NMR spectrum (CDCl_3_) of the PTA/3,3′-dimethyloxetane ROCOP product.

Taken together the formation of primary alkoxide intermediates is key to avoid O/S scrambling even if the backbiting step is kinetically available. To test this hypothesis, we employed a variety of 3,3′-geminally disubstituted oxetanes in ROCOP with PTA (see ESI Section S5†). For these, cyclisation *via* backbiting should be favoured, as angular compression effects in cyclisation reaction are most pronounced for geminal disubstitution. Furthermore, backbiting in the case of alkoxide intermediates originating from oxetane ring-opening would form six-membered cyclic intermediates rather than five-membered cyclic intermediates I as for epoxides. The former should be less strained and hence be more favoured to form leading to O/S scrambling. However, ring-opening of the investigated oxetanes though also results in the formation of primary alkoxides, which, according to our mode developed for epoxides, should overwrite the effects on cyclisation and lead to little O/S scrambling. Indeed, as can be seen in Table 2 and Fig. 5, an excellent linkage selectivity is obtained in quantitative polymer selectivity (*M*_n_ = 9.8–28.4 kg mol^−1^, *Đ* = 1.2–2.2) for every monomer investigated, confirming our model that primary alkoxide formation is most important to avoid O/S scrambling. Thereby, access to a variety of ester-*alt*-thioesters with even functional unsaturated substituents is achieved.§§Notably these selectivity trends are also observed in CS_2_ ROCOP which we also investigated as part of this study (see ESI Section S7†) albeit complicated by the fact that cyclic carbonate formation is also observed. In CS_2_/epoxide ROCOP under analogous conditions, epoxides which yield less O/S scrambling in PTA ROCOP lead to increased polymer selectivity during the reaction.

**Table 2:** LCrK (Cat. 2) catalysed PTA ROCOP with 3-Me/3-R-substituted oxetanes^a^

Run^b^	*R*	*t* [h]	Conv.^a^ [%]	Alt.^c^ [%]	*M*_n_ d [kDa] (*Ð*)
#1	Me	19	99	99	11.4 (1.2)
#2	CH_2_OEt	60	75	99	28.4 (2.2)
#3*	CH_2_OBn	72	80	97	9.8 (1.2)
#4	CH_2_OAllyl	220	60	99	16.2 (1.6)

a ROCOP conducted at 80 °C or *120 °C with 1 eq. LCrK: 1000 eq. 3-Me,3′-R-Oxetane: 1000 eq. PTA.

b Conversion calculated by comparing the relative integrals in the normalised ^1^H NMR spectrum (CDCl_3_, 400 MHz) of aromatic resonances due to polymer *versus* unconsumed PTA.

c Relative integrals in the ^13^C NMR spectrum (CDCl_3_, 126 MHz) from carbonyl resonances due to alternating ester-*alt*-thioester links compared to other carbonyl resonances.

d Determined by GPC (gel permeation chromatography) measurements conducted in THF, using narrow MW polystyrene standards to calibrate the instrument.

### On the occurrence of intermolecular scrambling

**Fig. 6:**
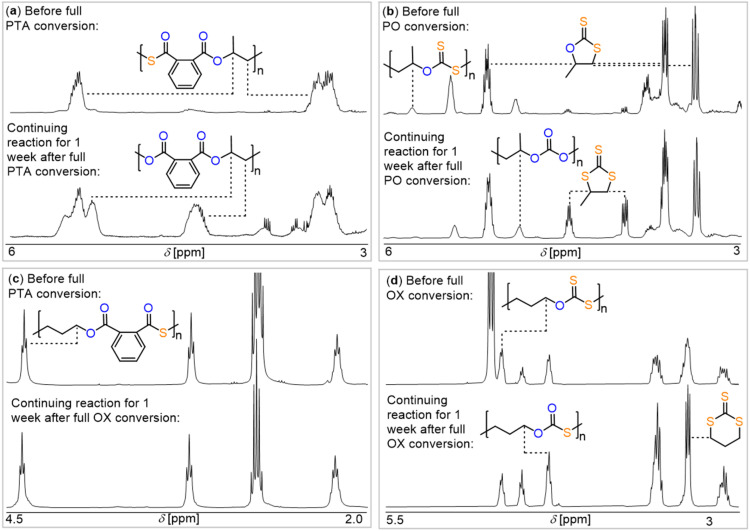
Zoom into the ^1^H NMR spectra (CDCl_3_) of the crude reaction mixture of (a) PTA/PO ROCOP performed at 1 SalCyCrCl: 1 PPNCl: 1000 PTA: 2000 PO: at 80 °C as well as (b) CS_2_/PO ROCOP performed at 1 SalCyCrCl: 1 PPNCl: 2000 CS_2_: 1000 PO: at 50 °C at different reaction stages. (c) PTA/OX ROCOP performed at 1 SalCyCrCl: 1 PPNCl: 1000 PTA: 2000 OX: at 80 °C as well as (d) CS_2_/OX ROCOP performed at 1 SalCyCrCl: 1 PPNCl: 1000 OX: 2000 CS_2_ at 50 °C at different reaction stages.

The fact that backbiting processes at the polymer chain end dominate the selectivity of the ROCOP process does, however, not completely exclude the possibility of the occurrence of intermolecular scrambling, as per Fig. 2(e). We investigated the change of the polymer microstructure once propagation ceases due to full monomer consumption (ESI Section S8†) as here all scrambling, as per Fig. 2(d), must have been finished. Therefore, we performed both PTA/PO and CS_2_/PO ROCOP with the commercial SalCyCrCl/PPNCl catalyst pair (Cat. 1; being most prone to scramble) well past full monomer conversion of one of the comonomers. Continuation of PTA/PO ROCOP (Fig. 6(a)) past the consumption of all PTA (bottom) leads to the clear emergence of diester links and this can be explained by scrambling reactions following thiirane elimination (Fig. 2). Continuing CS_2_/PO ROCOP past full PO consumption (Fig. 6(b)) leads to a continuation of the scrambling process, decreasing polymer signals and the formation of cyclic trithiocarbonate. At the end of the experiments the polymer exhibits a higher proportion of all-oxygen carbonate links than during propagation and the initially formed unscrambled dithiocarbonate links almost entirely disappeared. Clearly, linkage scrambling occurred once propagation ceased, leading to an oxygen enriched polymer *via* thiirane elimination pathways. The so produced thiirane then couples with residual CS_2_ explaining the formation of cyclic trithiocarbonate. A similar situation is observed for CS_2_/OX ROCOP (Fig. 6(d)). While the unscrambled OSS link is most dominant before full OX conversion, the oxygen enriched OOS link becomes most pronounced when the reaction is continued past full OX conversion. Furthermore, six-membered cyclic trithiocarbonate forms, which can be rationalized by some of the polymer being depolymerised to thietane, which is then coupled with CS_2_ yielding the heterocycle. The situation is different for PTA/OX ROCOP as here no scrambling occurs upon continuation of the reaction past full PTA conversion. The perfectly alternating poly(ester-*alt*-thioester) sequence is maintained 1 week after full OX conversion, and this suggests that these copolymers seem intrinsically more robust against scrambling even through intermolecular pathways. Yet, the underlying reasons for this are unclear at the current stage and require further investigation. Combined above results imply that some care must been taken when assessing the selectivity of new sulfurated ROCOP catalysts as the time at which the reaction is stopped can drastically influence the observed selectivity and this is most important for CS_2_ copolymerisations.

## Conclusion

In summary, through the exploration of a series of new monomer combinations in heterobimetallic ROCOP, we have developed a comprehensive mechanistic understanding of the factors governing linkage selectivity in the ROCOP of sulfur-containing monomers, independent of the catalyst employed. This has led to the identification of general guidelines to control scrambling side reactions and maximize the selectivity of sulfurated ROCOP:

(i) Avoid scrambling by selecting monomers that lead to primary alkoxide or thiolate formation upon ring opening.

(ii) Reduce scrambling by choosing bicyclic monomers.

(iii) Minimize scrambling by terminating the reaction before full monomer consumption.

By following these guidelines, we achieved the highly selective synthesis of a series of new poly(thioesters). In a more general sense, we demonstrated that the optimisation of catalyst independent parameters rather than catalyst optimisation can be a successful strategy to establish selective catalytic processes. Our findings, hence, provide valuable guidance for designing selective polymerization strategies, advancing the development of new sulfur-containing polymers as potential substitutes for current commodity materials.

## Author contributions

M. R. S., M. K., and C. F.-W. performed the experimental work. S. M. R. conducted the XRD analysis, and R. L. carried out the DFT calculations. M. R. S., R. L., and A. J. P. wrote the supporting information and main manuscript. A. J. P. conceived the project, supervised the research, and secured funding.

## Conflicts of interest

There are no conflicts of interest.

## Supplementary Material

SC-015-D4SC05858E-s001

SC-015-D4SC05858E-s002

## Data Availability

The data supporting this article have been included as part of the ESI.†
