# Supplementary material for: Monomer centred selectivity guidelines for sulfurated ring-opening copolymerisations
Source: Chem Sci. 2024 Oct 24;15(45):19029–36. doi: 10.1039/d4sc05858e (PMC11515943; doi:10.1039/d4sc05858e)
Supplement: SC-015-D4SC05858E-s001 [file SC-015-D4SC05858E-s001.pdf]

Supplementary Information (SI) for Chemical Science.
This journal is © The Royal Society of Chemistry 2024

Supplementary Information

Table of Contents

Section S1: Methods 3
Section S2: Introductory Experiments 4
Section S3: Epoxide/PTA Copolymerisations 11
Section S4: Thiirane/PTA Copolymerisations 36
Section S5: Oxetane/PTA Copolymerisations 40
Section S6: DFT Studies 54
Section S7: Epoxide/CS_2 Copolymerisations 54
Section S8: Exceeding Reaction Times for (PO,OX)/(PTA,CS_2) Copolymerisations 58
Section S9: Crystallography 61
Section S10: Bibliography 62

Section S1: Methods

Solvents and reagents were obtained from commercial sources and used as received unless stated otherwise.

All epoxides were dried over calcium hydride at room temperature for at least 3 days, vacuum transferred and stored inside an argon filled glovebox prior to use. Oxetane was dried over calcium hydride and elemental sodium and stored inside an argon filled glovebox prior to use. The other oxetanes employed (synthesized previously outlined[1]) were either dried over 4Å molecular sieves or calcium hydride and stored inside an argon filled glovebox prior to use.

Phthalic acid thioanhydride (PTA) was synthesized by an adapted literature procedure:[2] Phthalic anhydride (0.3 mol, 1.0 equiv) was dissolved in 600 mL THF and an aqueous $Na_2S \cdot 9H_2O$ (0.2 mol, 0.7 equiv) solution was added slowly. The resulting mixture was stirred at room temperature and the reaction progress was monitored by NMR analysis. After all phthalic anhydride was consumed, THF was removed in vacuum and the aqueous phase was extracted 3 times with DCM and dried over $MgSO_4$. The crude thioanhydride was recrystallized twice from *tert*-butyl methyl ether and further purified by sublimation and stored inside a glovebox.

PPNCl was recrystallized from dry CH_2Cl_2 and dried in vacuum prior to use. The catalyst LCrK was synthesized as previously reported.[1]

NMR spectra were recorded by using a Jeol JNM-ECA 400II or ECZ600, Bruker Advance 600 and 700 MHz spectrometer. ^1H and $^{13}C\{^1H\}$ chemical shifts are referenced to the residual proton resonance of the deuterated solvents. Differential scanning calorimetry (DSC) was measured on a Netzsch 204 F1 "Phoenix" at a heating rate of 10.0 K/min. DSC thermograms are presented for the data obtained after the first heating cycle to ensure removal of the thermal history of the sample. The molecular mass and polydispersity of the polymers were determined by a Waters 1515 Gel permeation chromatography (GPC) instrument equipped with two linear PLgel columns (Mixed-C) following a guard column and a differential refractive index detector using tetrahydrofuran as the eluent at a flow rate of 1.0 mL/min at 30 °C and a series of narrow polystyrene standards for the calibration of the columns. Each polymer sample was dissolved in HPLC-grade THF (6 mg/mL) and filtered through a 0.20 µm porous filter frit prior to analysis.

General polymerisation protocol:

The catalyst and the monomers were added to a flame dried vial equipped with a flame dried stirrer bar and sealed with a melamine cap containing a Teflon inlay. The vial was brought outside the glovebox and placed in a pre-heated aluminium block at the specified temperature for the specified time. At the specified end point of the reaction, the polymerisation mixture was cooled down to room temperature and an aliquot was removed and analysed by ^1H NMR for the determination of the conversion. The mixture was solubilized with ca. 5 ml of DCM and then added to 50 mL of MeOH causing the precipitation of the polymer which was isolated by centrifugation, washed with pentane and dried in a vacuum oven.

Section S2: Introductory Experiments

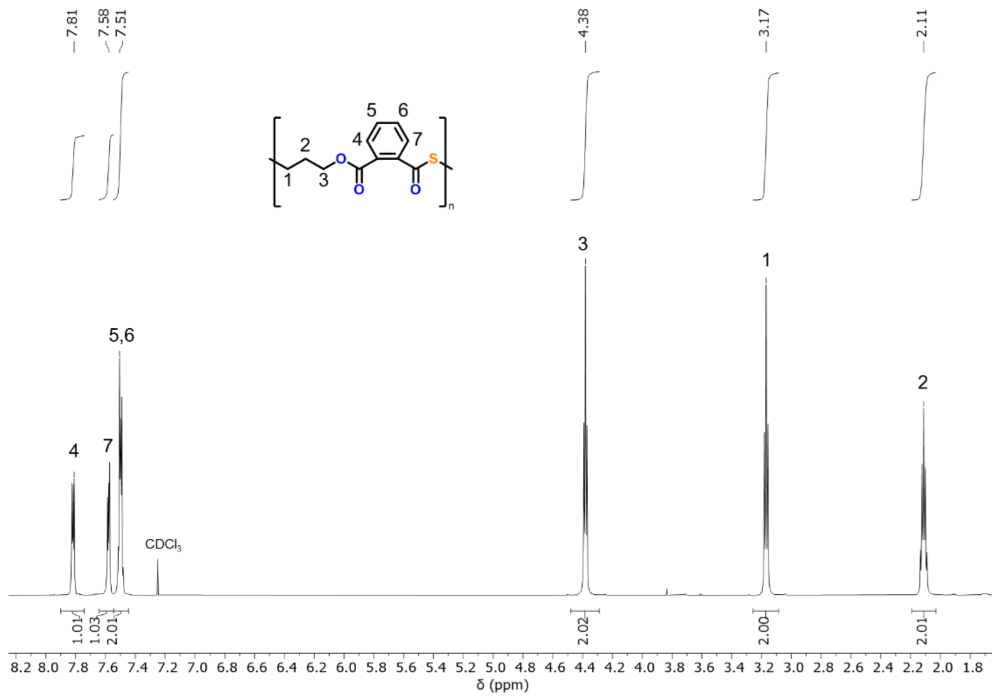

Figure S 1: ^{1}H NMR spectrum (600 MHz, $CDCl_3$, 25°C) of the precipitated polymer obtained from OX/PTA ROCOP using the bicomponent metal catalyst Cat 1 showing a selective poly(ester-*alt*-thioester) formation.

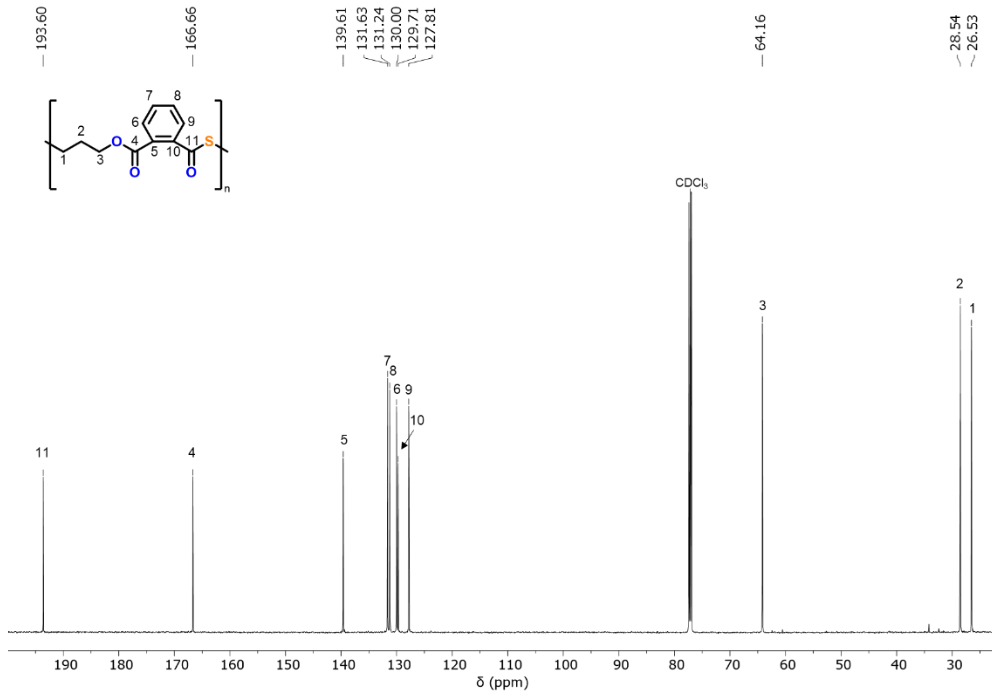

Figure S 2: ^{13}C NMR spectrum (151 MHz, $CDCl_3$, 25°C) of the precipitated polymer obtained from OX/PTA ROCOP using the bicomponent metal catalyst Cat 1.

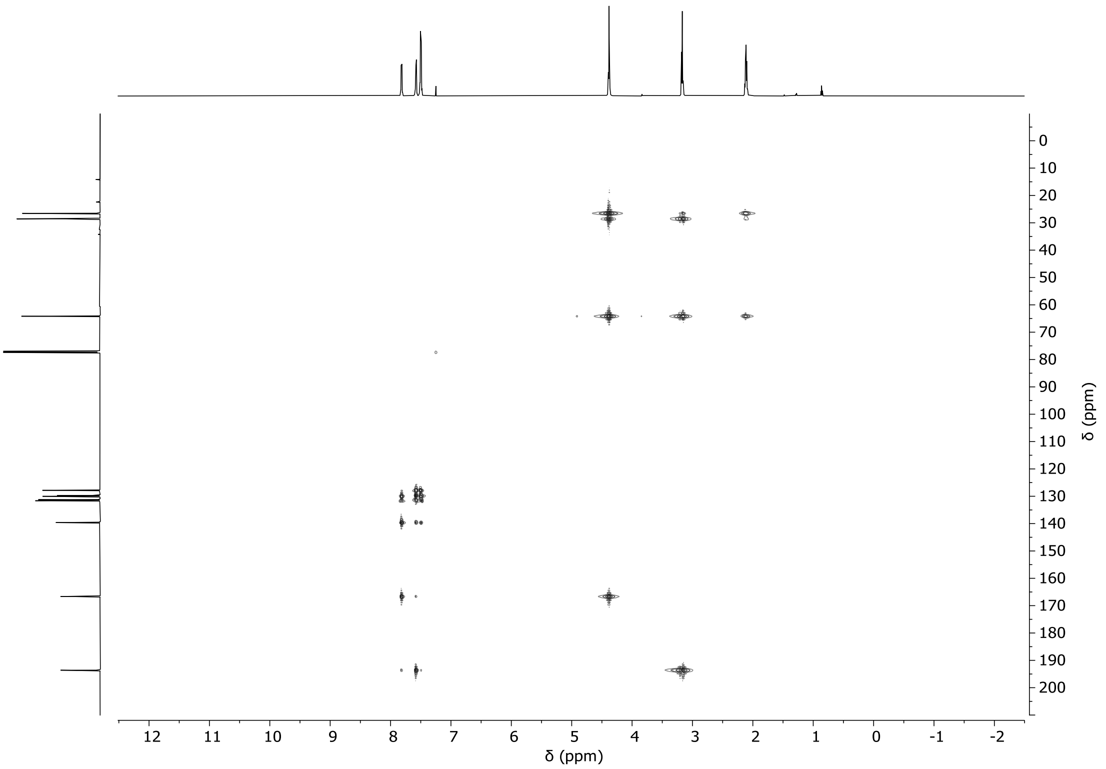

Figure S 3: ^{1}H-^{13}C HMBC NMR spectrum ($CDCl_3$, 25°C) of the precipitated polymer obtained from OX/PTA ROCOP using the bicomponent metal catalyst Cat 1.

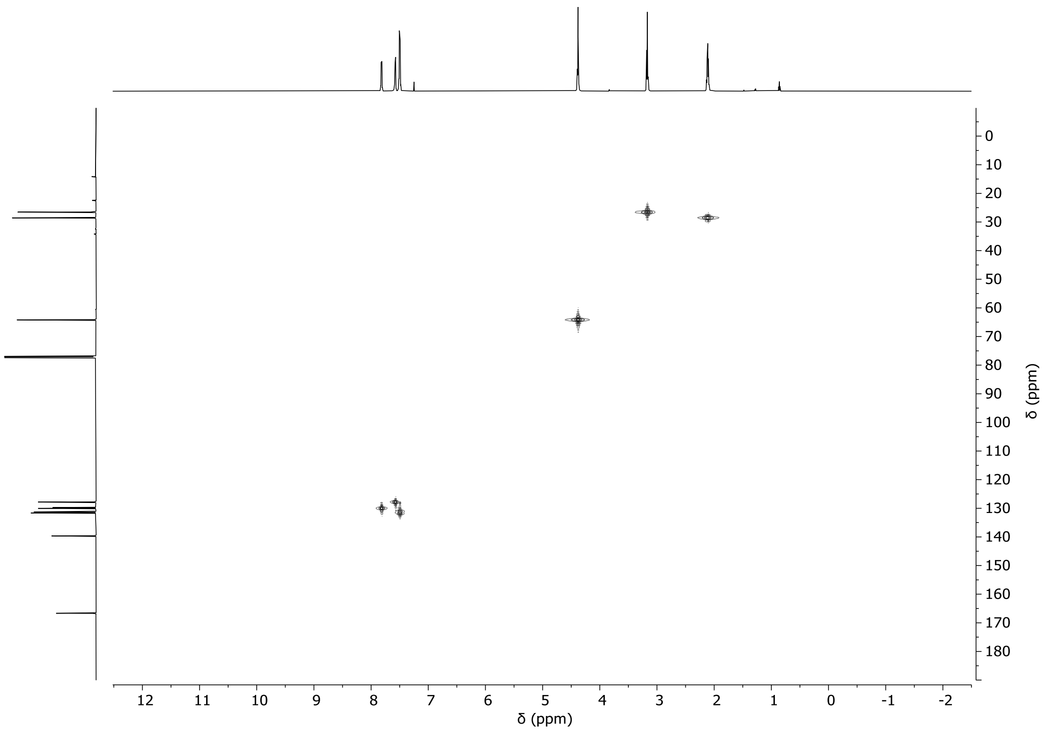

Figure S 4: ^{1}H-^{13}C HMQC NMR spectrum ($CDCl_3$, 25°C) of the precipitated polymer obtained from OX/PTA ROCOP using the bicomponent metal catalyst Cat 1.

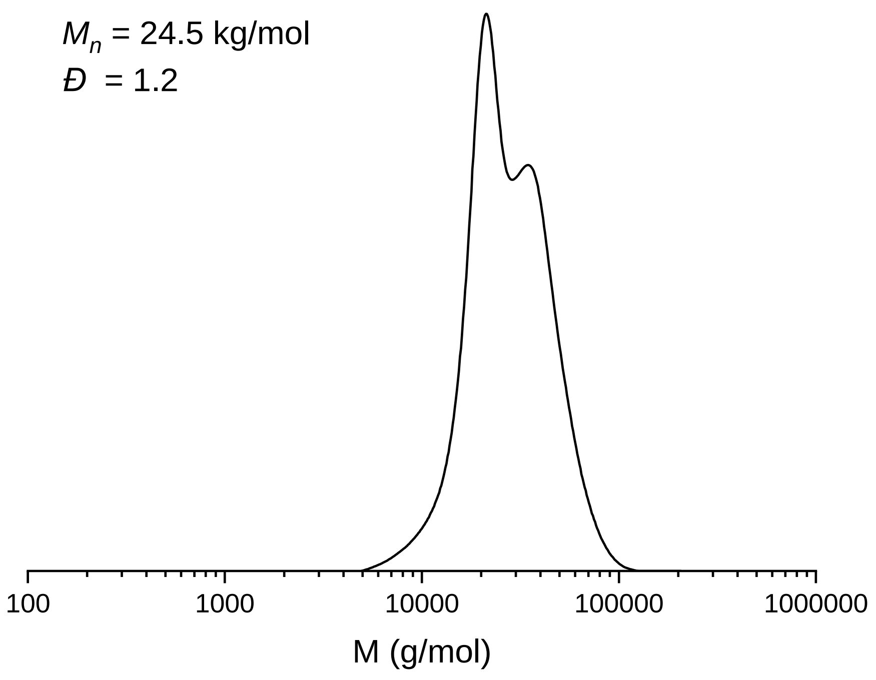

Figure S 5: GPC trace corresponding to the polymer obtained from OX/PTA ROCOP using the bicomponent metal catalyst Cat 1.

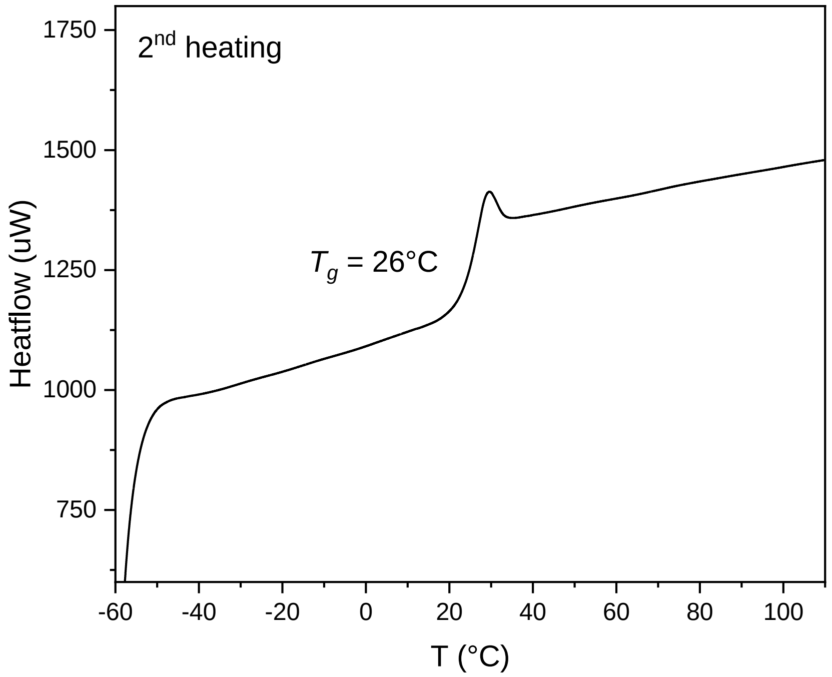

Figure S 6: DSC heating curve of the polymer obtained from OX/PTA ROCOP using the bicomponent metal catalyst Cat 1.

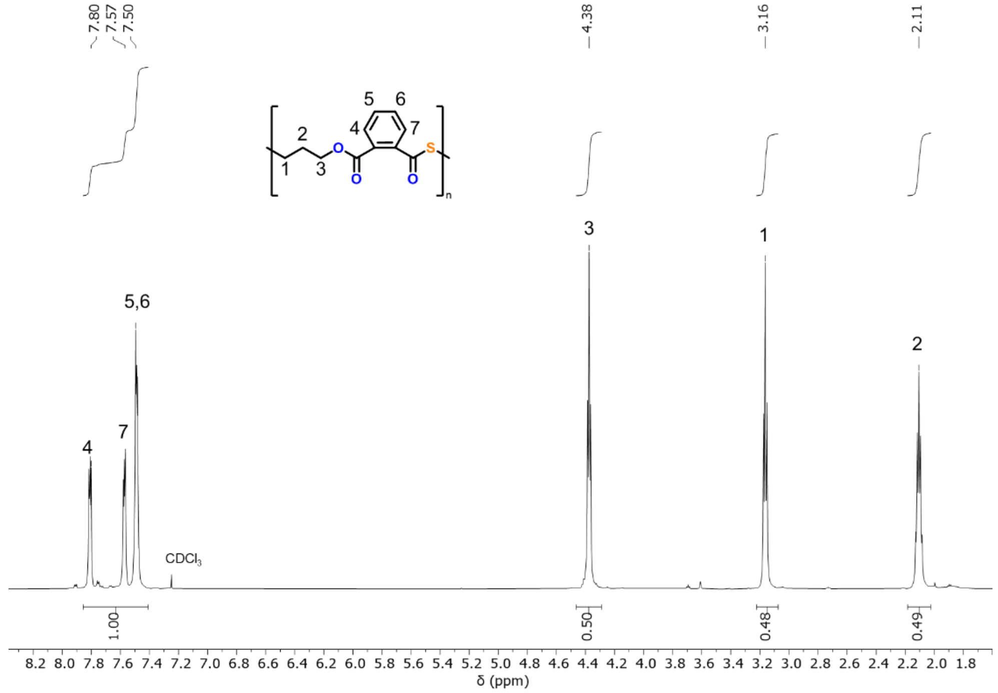

Figure S 7: ^{1}H NMR spectrum (600 MHz, $CDCl_3$, 25°C) of the precipitated polymer obtained from OX/PTA ROCOP using the bicomponent organocatalyst Cat 3 showing a selective poly(ester-*alt*-thioester) formation.

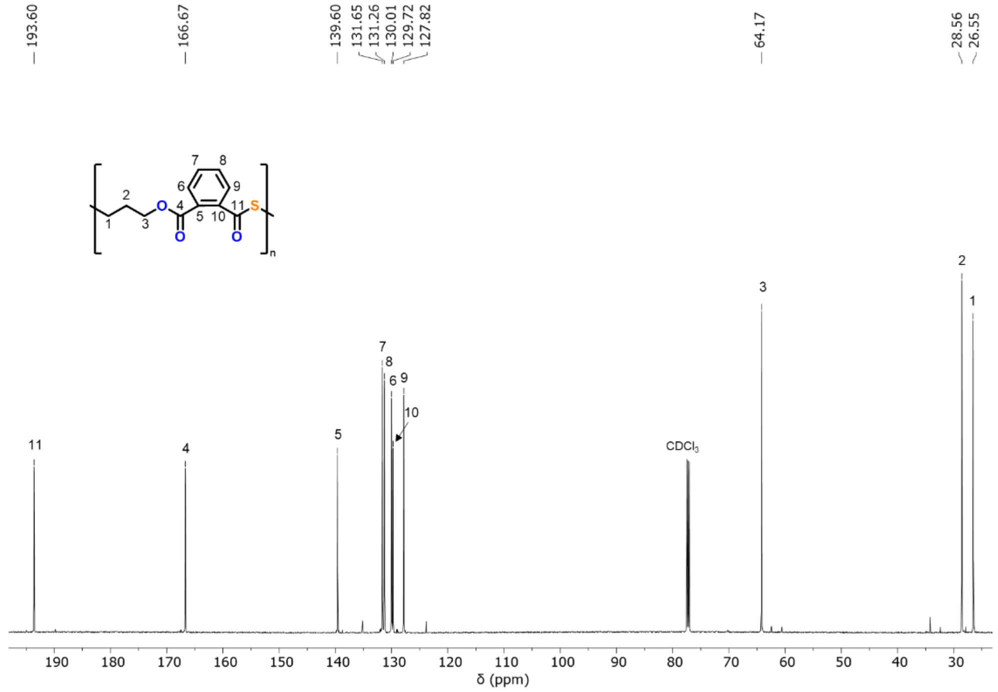

Figure S 8: ^{13}C NMR spectrum (151 MHz, $CDCl_3$, 25°C) of the precipitated polymer obtained from OX/PTA ROCOP using the bicomponent organocatalyst Cat 3.

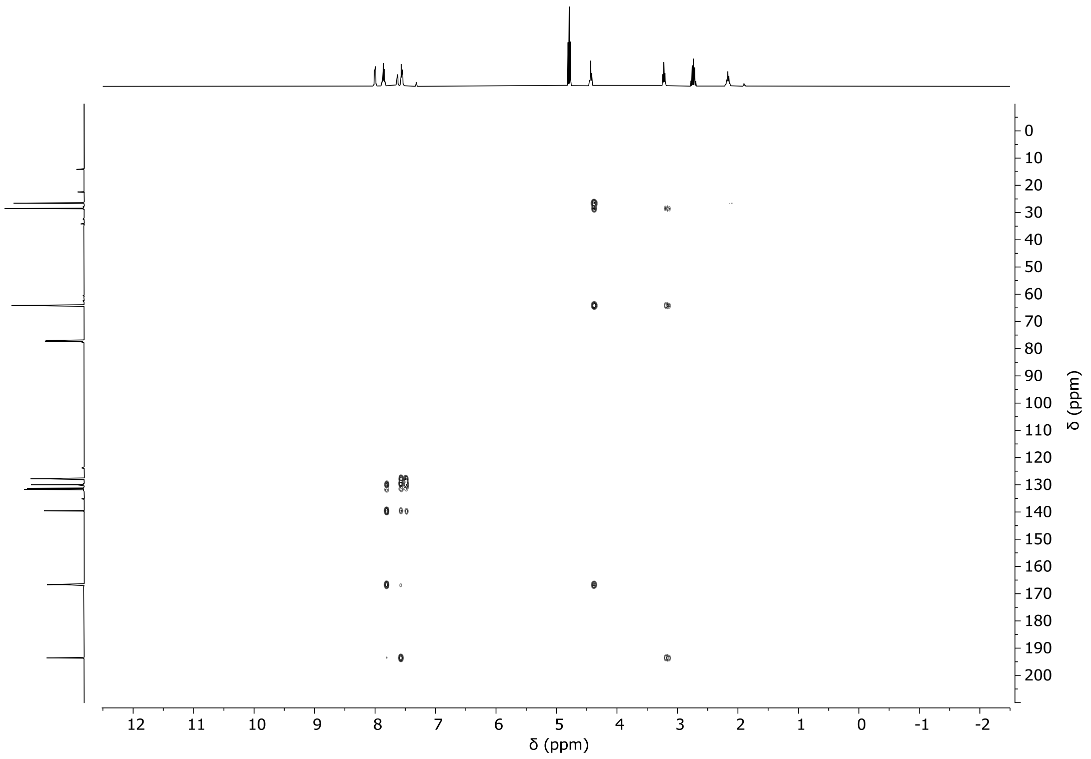

Figure S 9: ^{1}H-^{13}C HMBC NMR spectrum ($CDCl_3$, 25°C) of the precipitated polymer obtained from OX/PTA ROCOP using the bicomponent organocatalyst Cat 3.

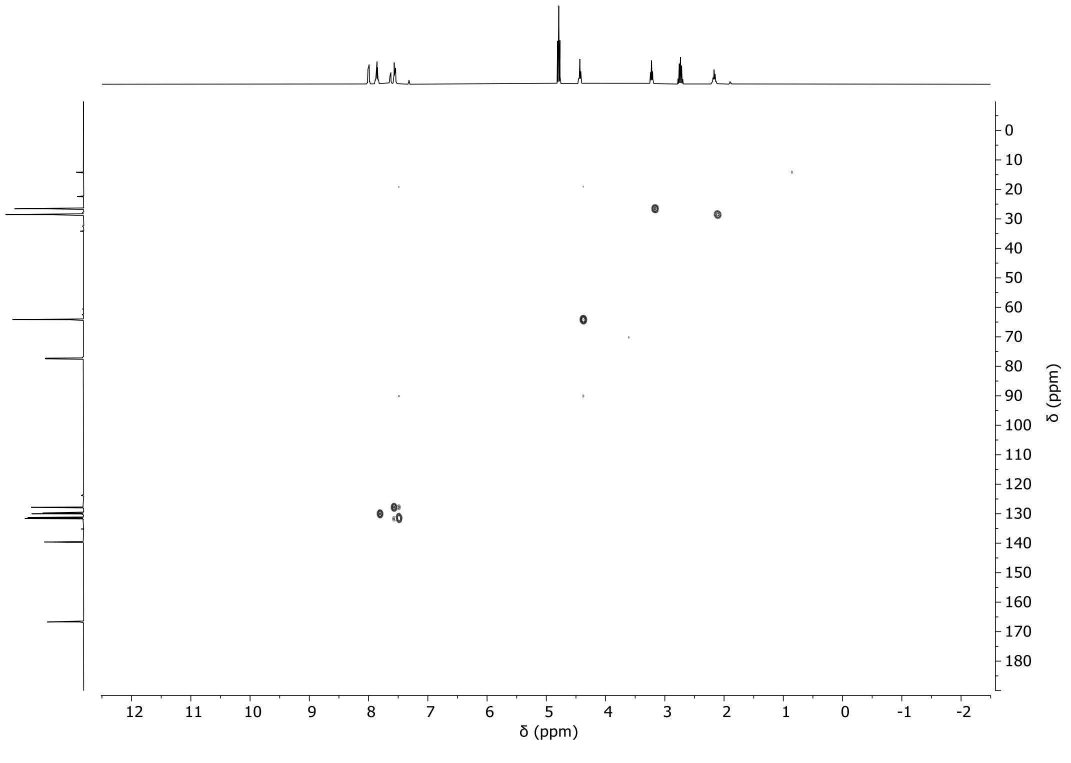

Figure S 10: ^{1}H-^{13}C HMQC NMR spectrum ($CDCl_3$, 25°C) of the precipitated polymer obtained from OX/PTA ROCOP using the bicomponent organocatalyst Cat 3.

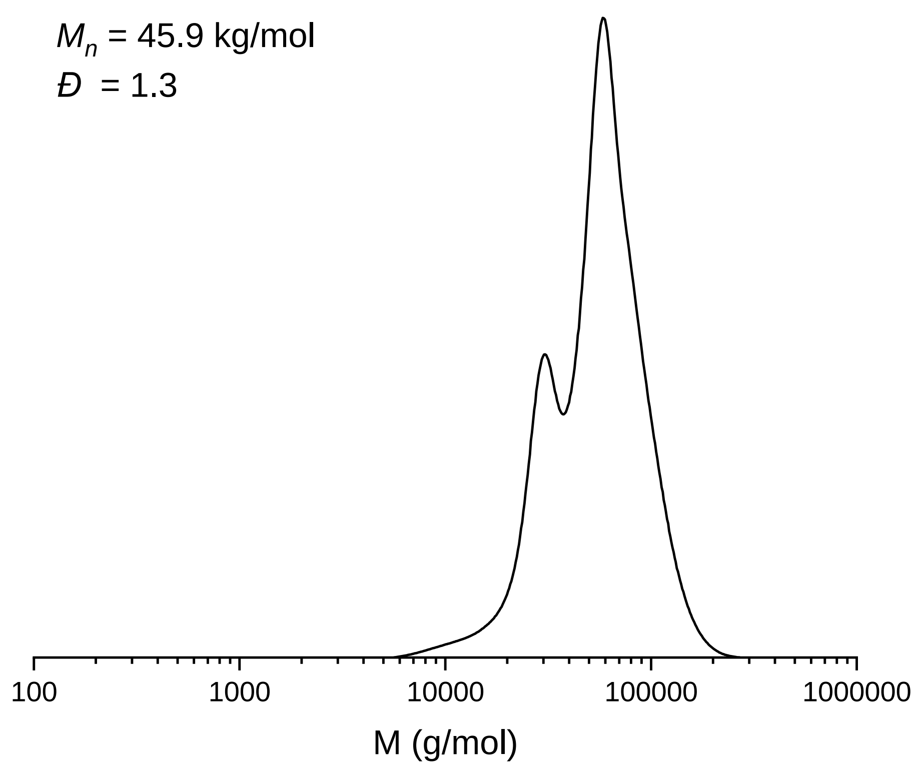

Figure S 11: GPC trace corresponding to the polymer obtained from OX/PTA ROCOP using the bicomponent organocatalyst Cat 3.

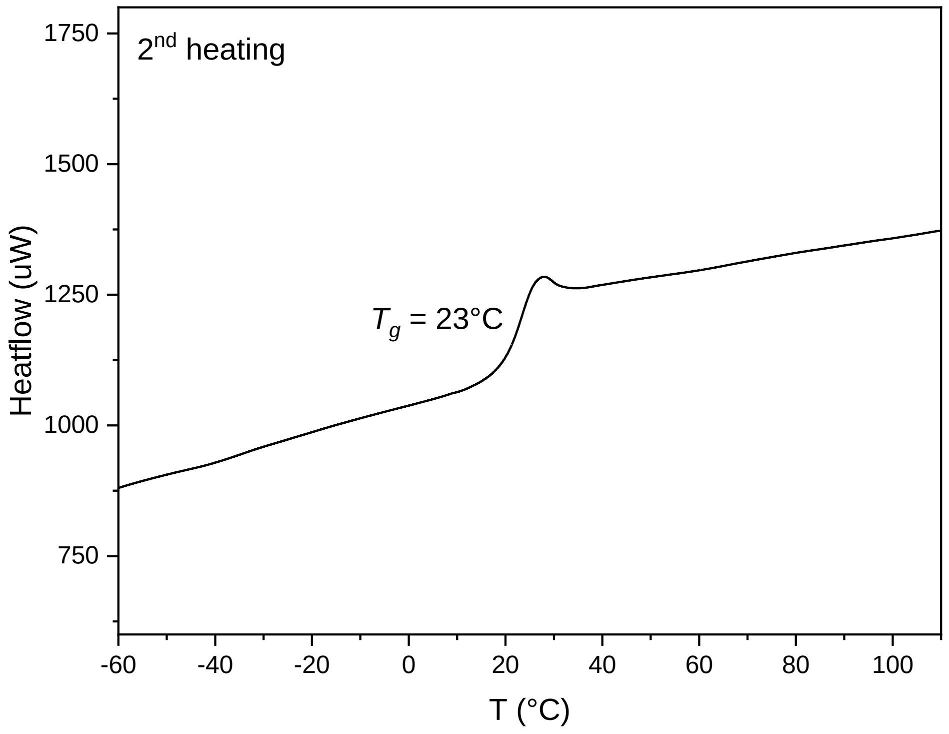

Figure S 12: DSC heating curve of the polymer obtained from OX/PTA ROCOP using the bicomponent organocatalyst Cat 3.

Determination of spin-lattice relaxation times:

In order to verify the quantitative interpretability of the relative integrals of the carbonyl resonances in the ^{13}C NMR spectra, investigations of their spin-lattice relaxation times were performed. For this purpose, the PO/PTA copolymer (Table 1, run #2) was selected as representative since it contains all four types of carbonyl resonances due to ester-*alt*-thioester, as well as diester and dithioester linkages.

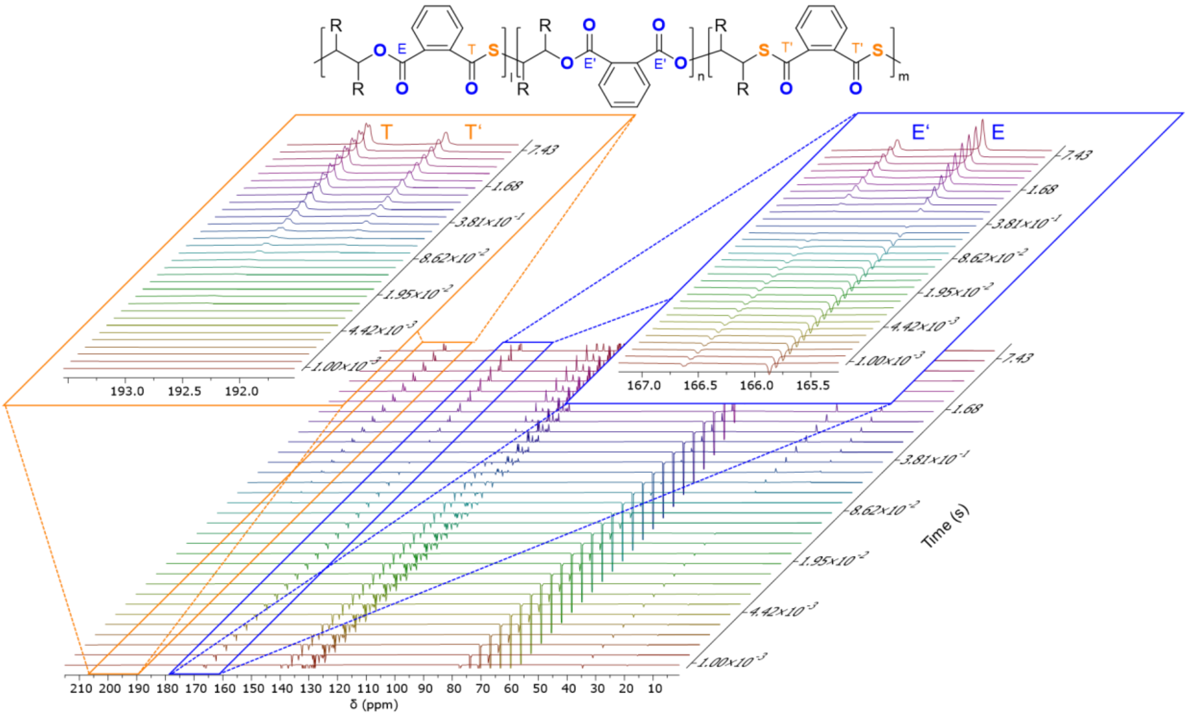

Figure S 13: Stacked time-dependent ^{13}C NMR measurements of the polymer corresponding to Tabe 1, run #2 that were used to determine the spin-lattice relaxation times of the carbonyl resonances.

The collection was started after 1 ms and run until 10 s. The different carbonyl resonances were plotted individually (magnetization vector M_z *versus* time) and a three-parameter exponential fit of B+F*exp(-x*G) was employed. Since the signal M(t) is defined by the equation of $M(t) = M_0(1-2*\exp(-t/T_1))$, where t is the inversion recovery delay and M_0 is the signal at thermal equilibrium state, the spin-lattice relaxation time T_1 is equal to 1/G. The analysis was performed using the built-in data analysis tool for T_1 measurements in MestReNova (Mestrelab Research S.L.). The results are shown in table S 1.

Table S 1: Fitting parameter and spin-lattice relaxation times.

Linkage type[1]	Fitting parameter B; F; G	Relaxation time T_1 [s]
Ester (E)	0.0053; -0.0072; 0.6622	1.51
Diester (E‘)	0.0026; -0.0034; 0.6446	1.55

Thioester (T)	0.0059; -0.0058; 0.7515	1.33
Dithioester (T‘)	0.0026; -0.0026; 0.7745	1.29

[1]Chemical shifts for each linkage type selected for analysis: ester (165 ppm), diester (166 ppm), dithioester (191 ppm) and thioester (192 ppm).

Since the duration of the standard NMR measurements performed in this study (2 s) exceeds the relaxation times of the carbonyl resonances, we can therefore confirm that integration of these signals in the ^{13}C NMR spectra can be interpreted quantitatively.

Section S3: Epoxide/PTA Copolymerisations

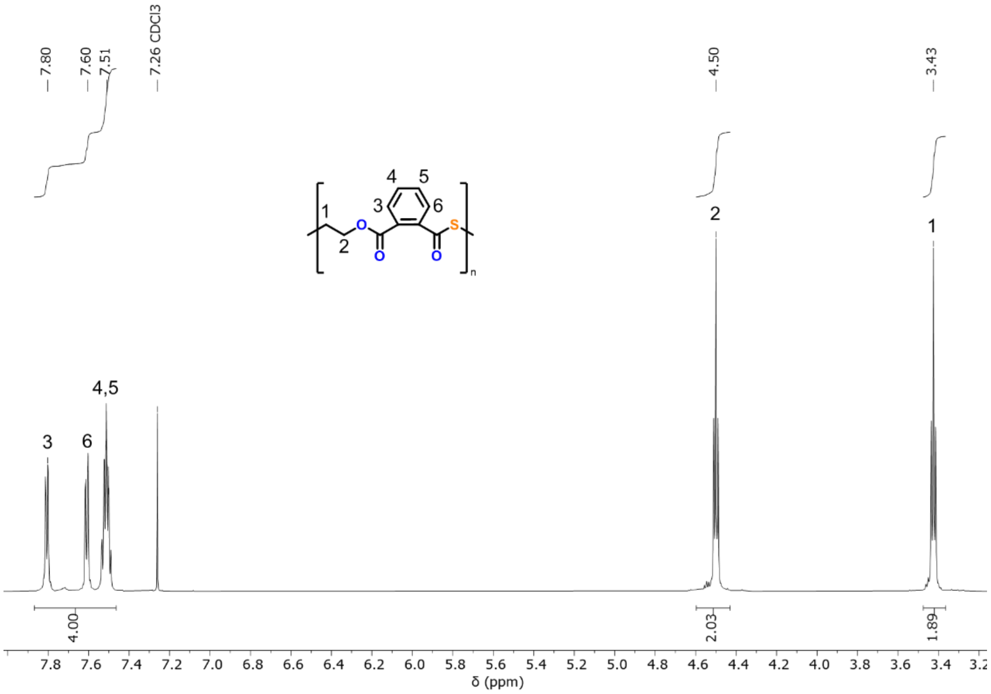

Figure S 14: ^{1}H NMR spectrum (600 MHz, $CDCl_3$, 25°C) of the precipitated polymer corresponding to table 1, run #1 showing a selective poly(ester-*alt*-thioester) formation.

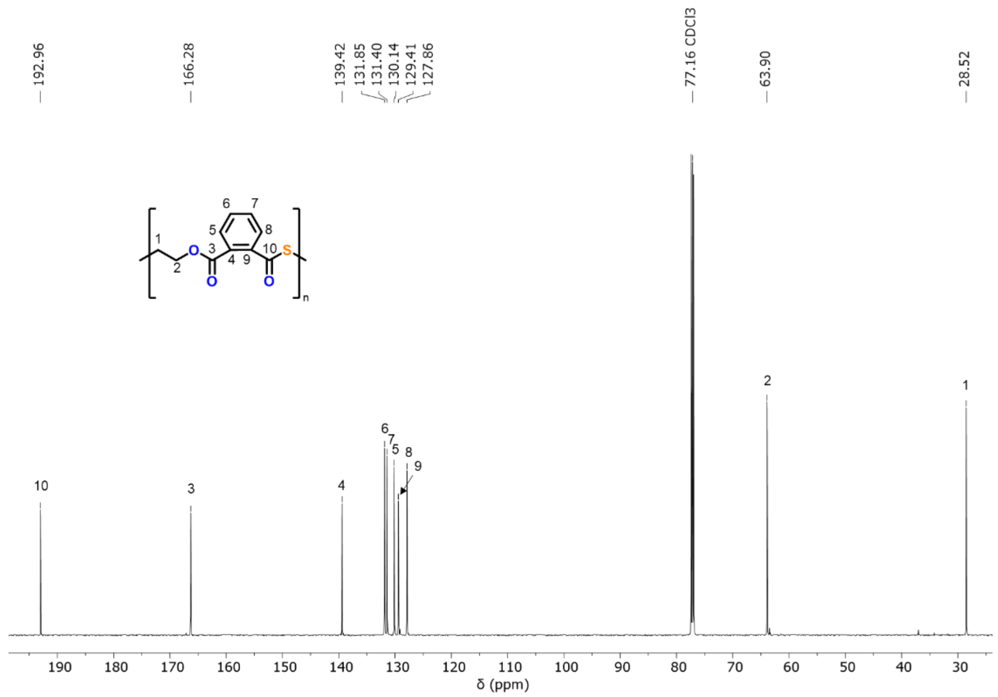

Figure S 15: ^{13}C NMR spectrum (151 MHz, $CDCl_3$, 25°C) of the precipitated polymer corresponding to table 1, run #1 showing a selective poly(ester-*alt*-thioester) formation.

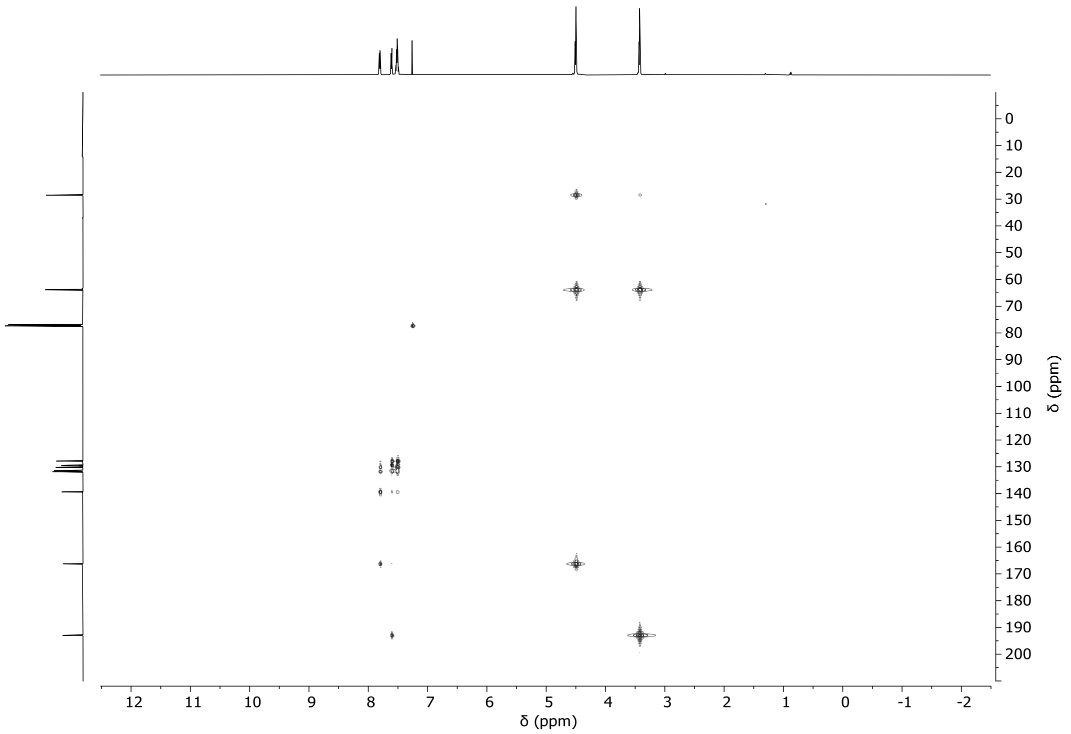

Figure S 16: ^{1}H-^{13}C HMBC NMR spectrum ($CDCl_3$, 25°C) of the precipitated polymer corresponding to table 1, run #1.

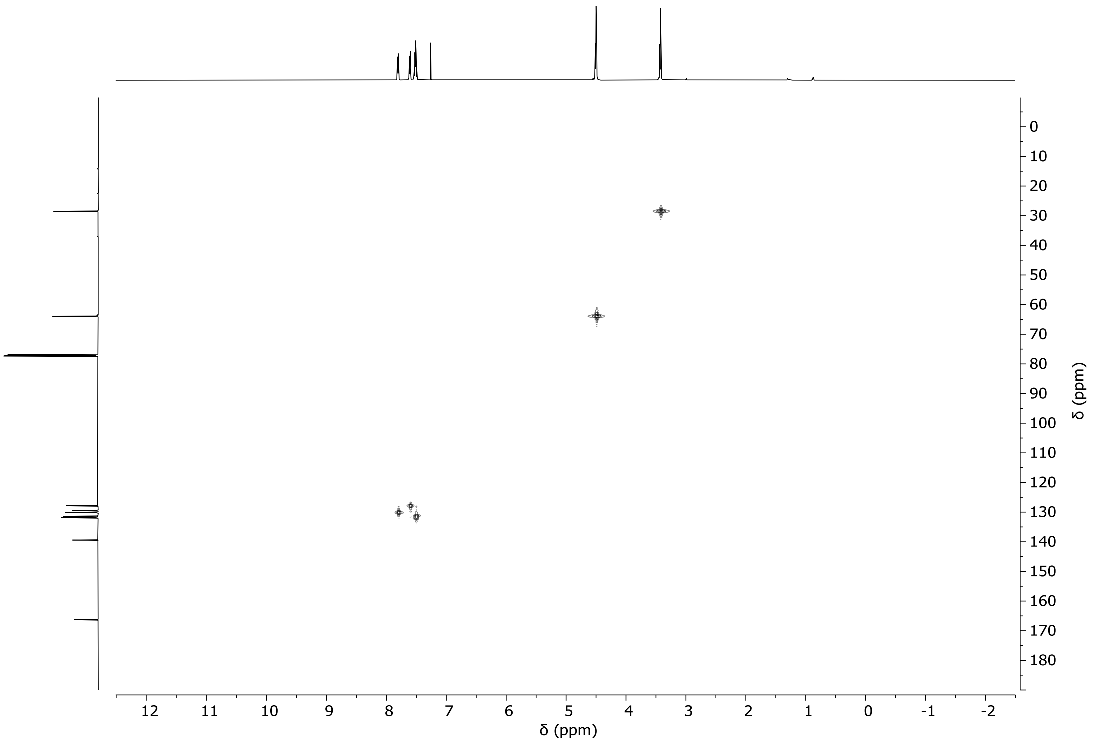

Figure S 17: ^1H-^{13}C HMQC NMR spectrum ($CDCl_3$, 25°C) of the precipitated polymer corresponding to table 1, run #1.

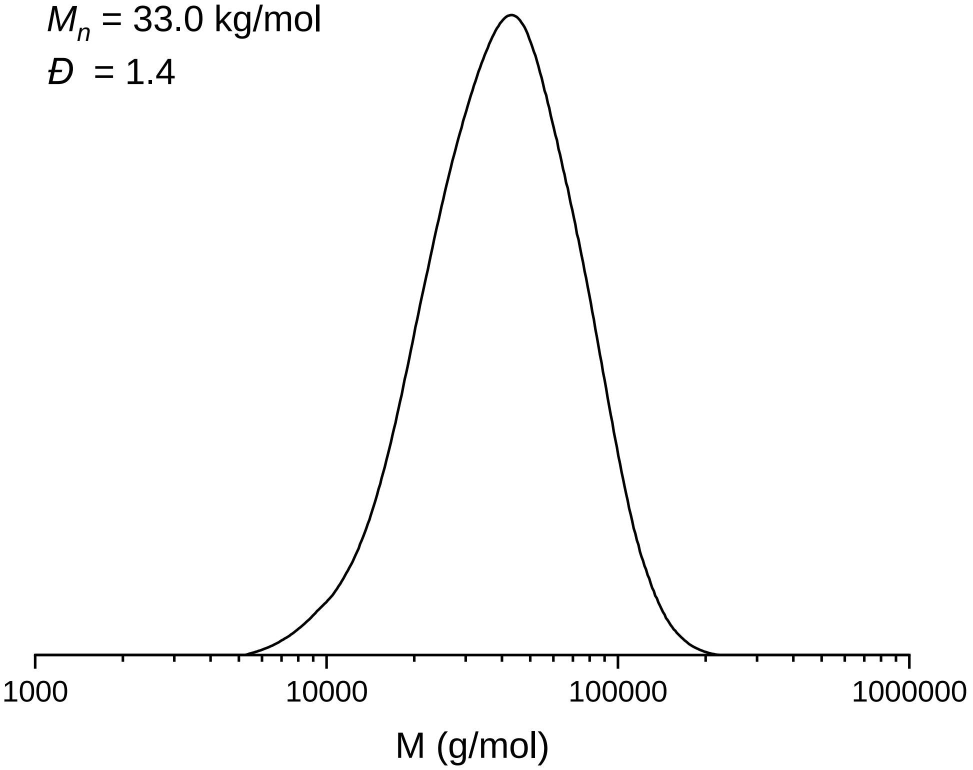

Figure S 18: GPC trace corresponding to table 1, run #1.

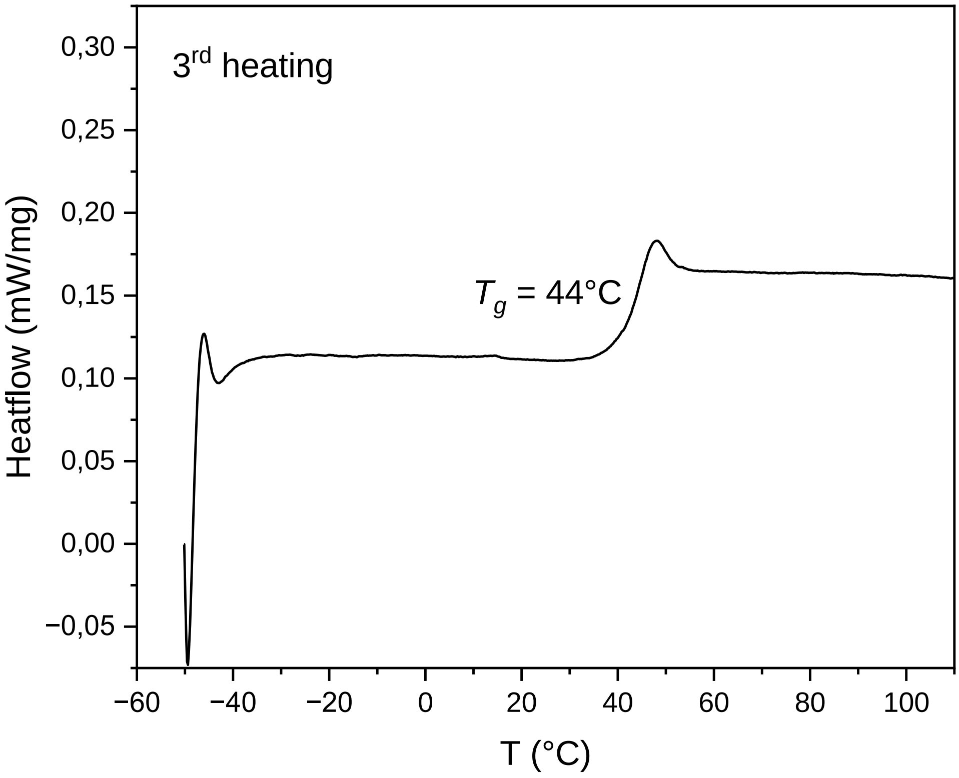

Figure S 19: DSC heating curve of the polymer corresponding table 1, run #1.

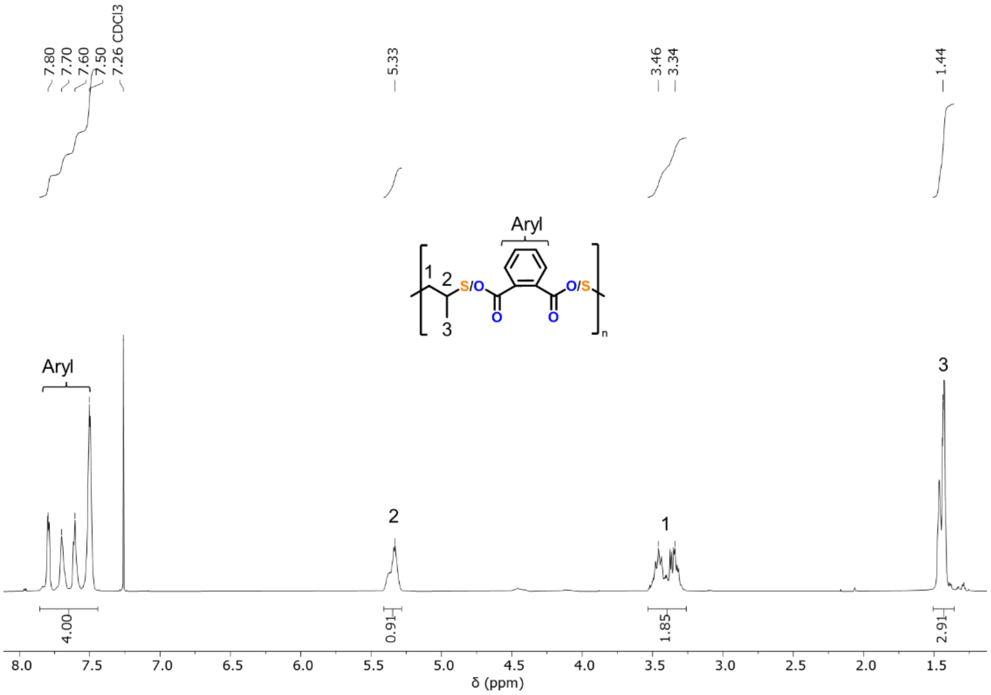

Figure S 20: ^{1}H NMR spectrum (600 MHz, $CDCl_3$, 25°C) of the precipitated polymer corresponding to table 1, run #2.

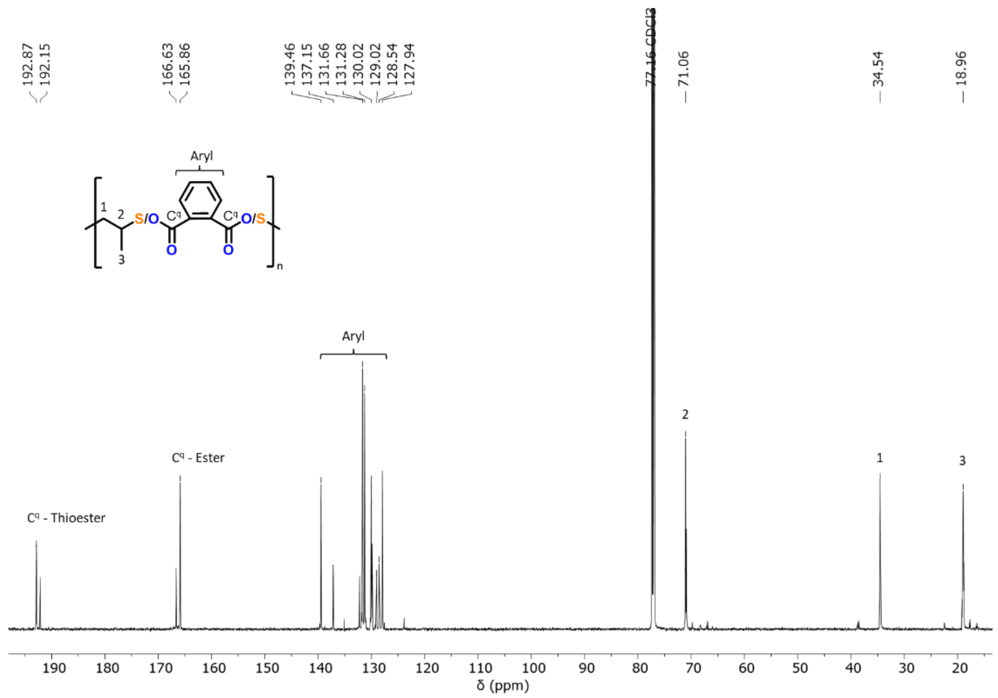

Figure S 21: ^{13}C NMR spectrum (151 MHz, $CDCl_3$, 25°C) of the precipitated polymer corresponding to table 1, run #2 showing an ester-*alt*-thioester selectivity of 67%.

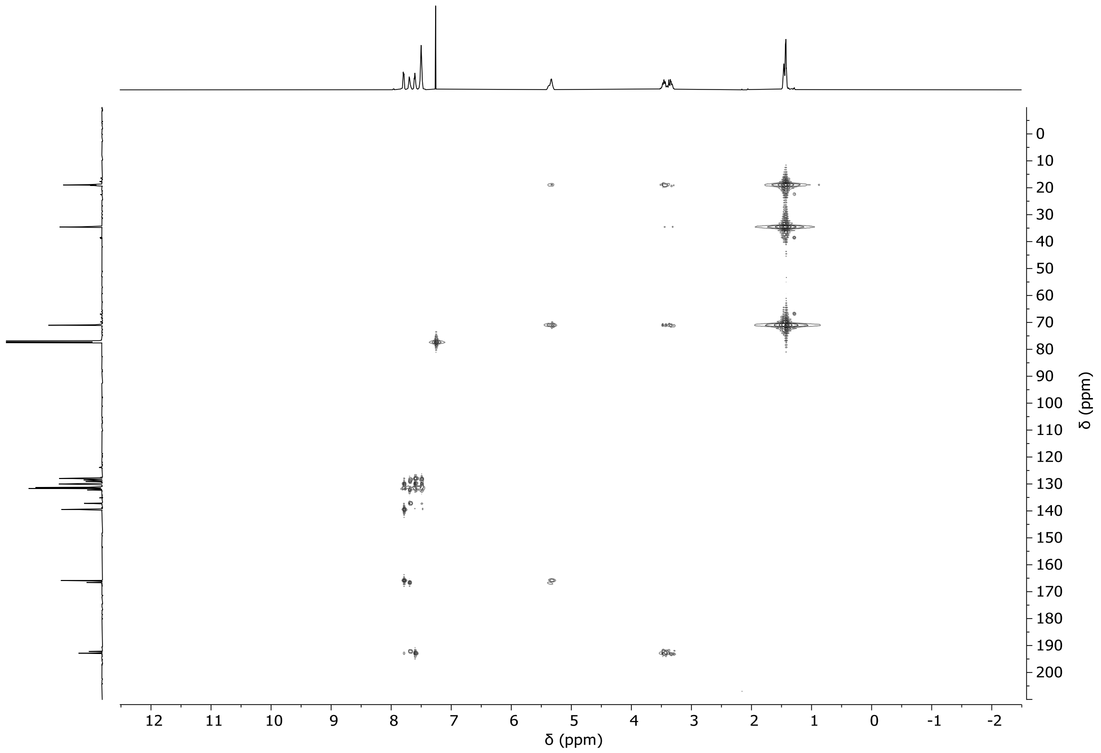

Figure S 22: ^{1}H-^{13}C HMBC NMR spectrum ($CDCl_3$, 25°C) of the precipitated polymer corresponding to table 1, run #2.

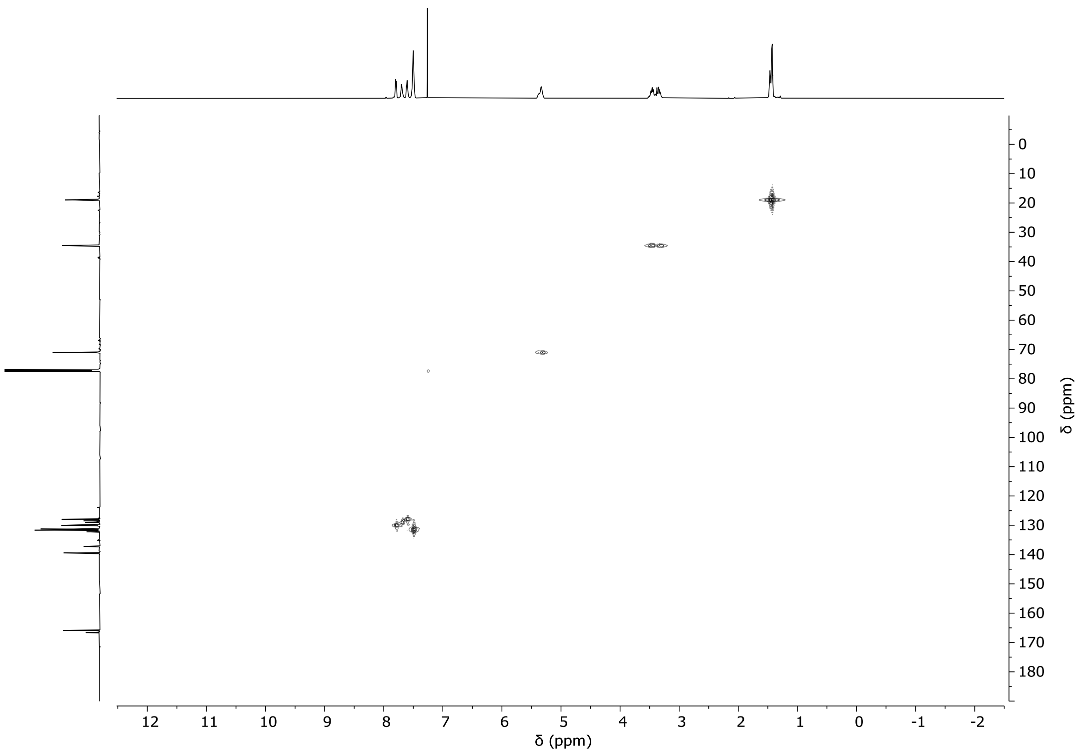

Figure S 23: ^{1}H-^{13}C HMQC NMR spectrum ($CDCl_3$, 25°C) of the precipitated polymer corresponding to table 1, run #2.

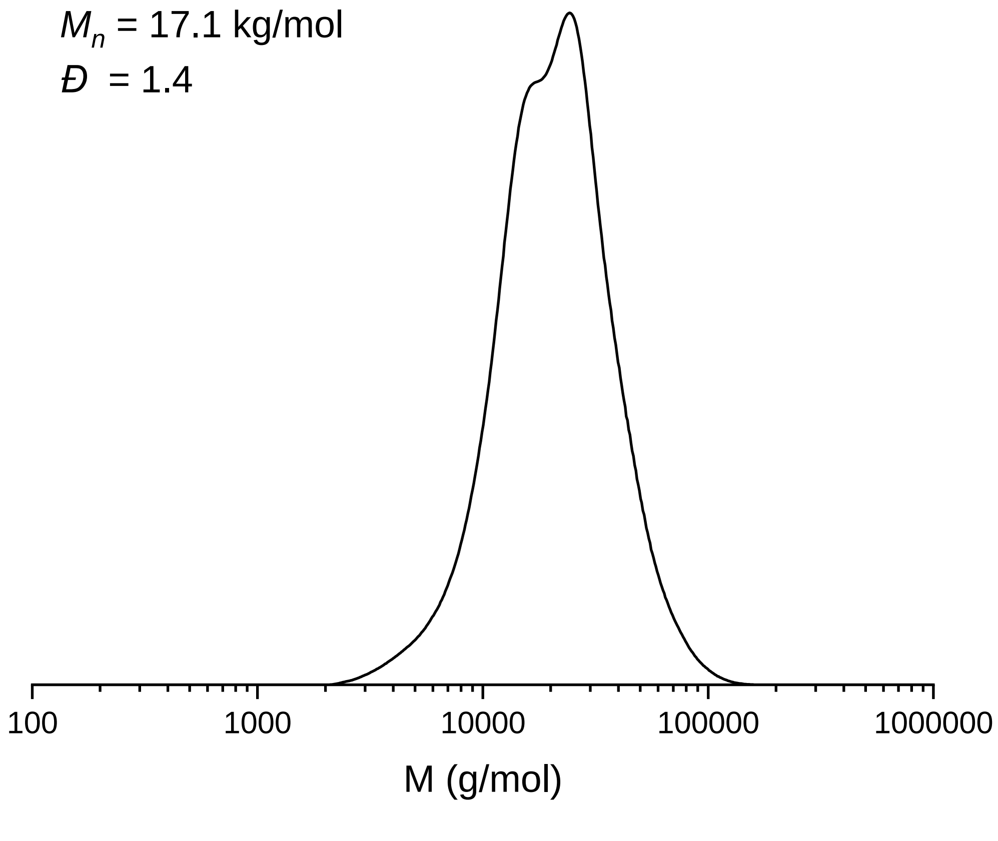

Figure S 24: GPC trace corresponding to table 1, run #2.

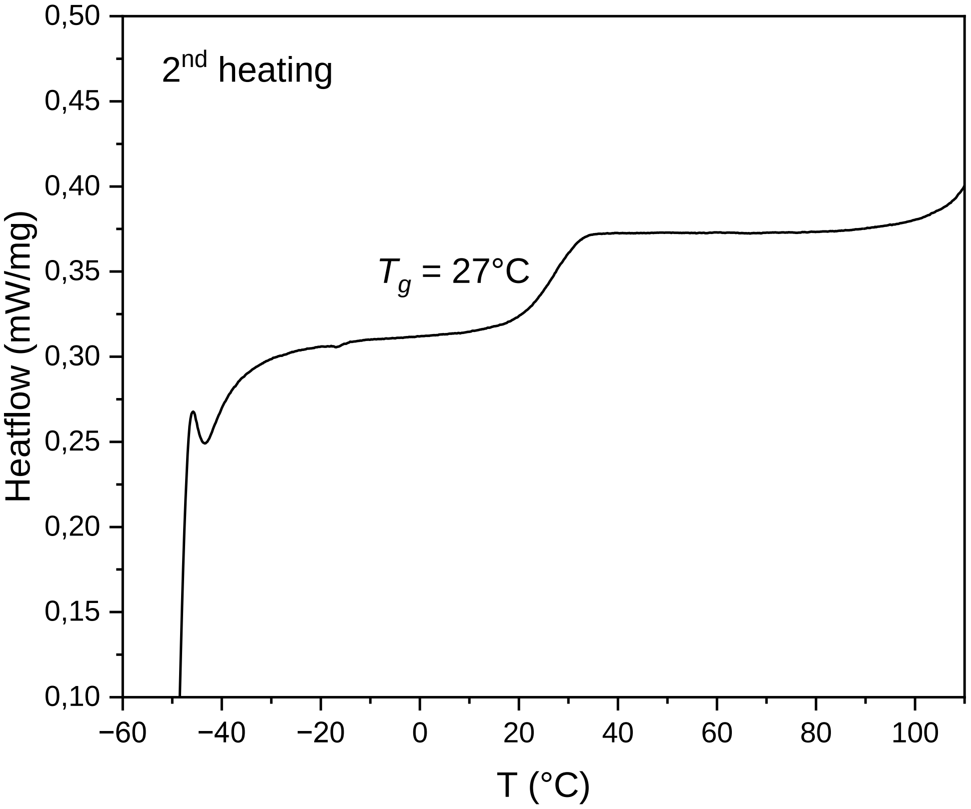

Figure S 25: DSC heating curve of the polymer corresponding table 1, run #2.

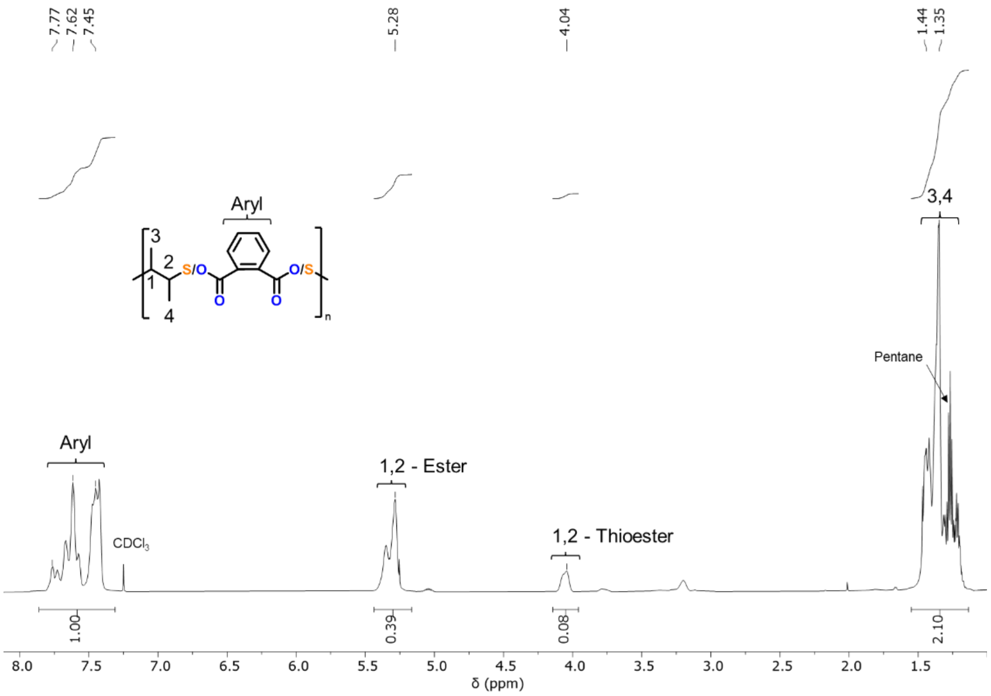

Figure S 26: ^{1}H NMR spectrum (600 MHz, $CDCl_3$, 25°C) of the precipitated polymer corresponding to table 1, run #3.

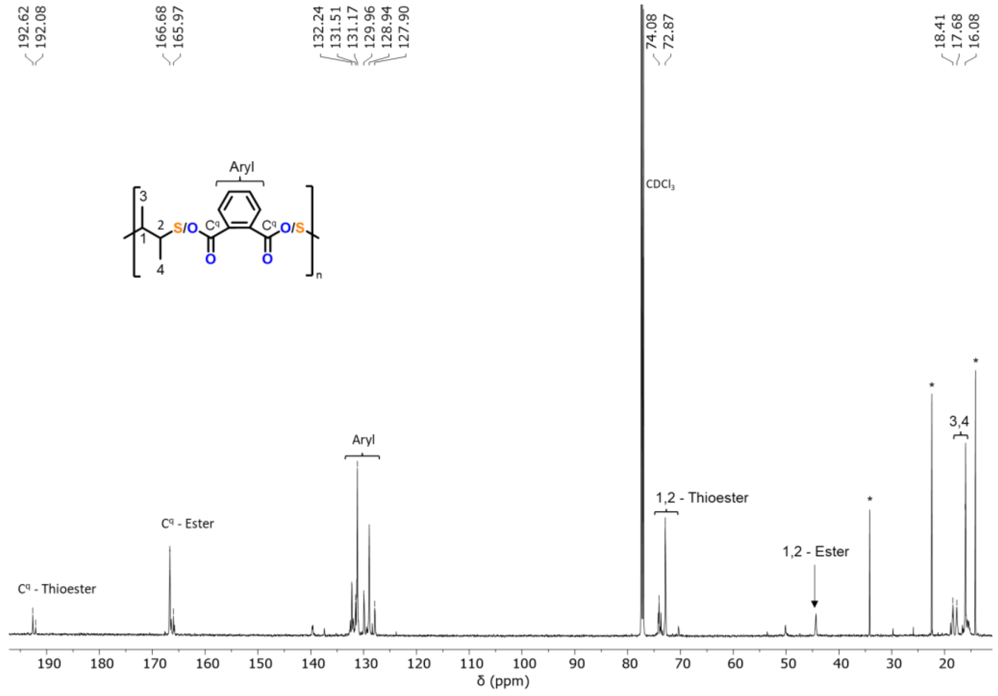

Figure S 27: ^{13}C NMR spectrum (151 MHz, $CDCl_3$, 25°C) of the precipitated polymer corresponding to table 1, run #3 showing an ester-*alt*-thioester selectivity of 28%. *denotes residual pentane.

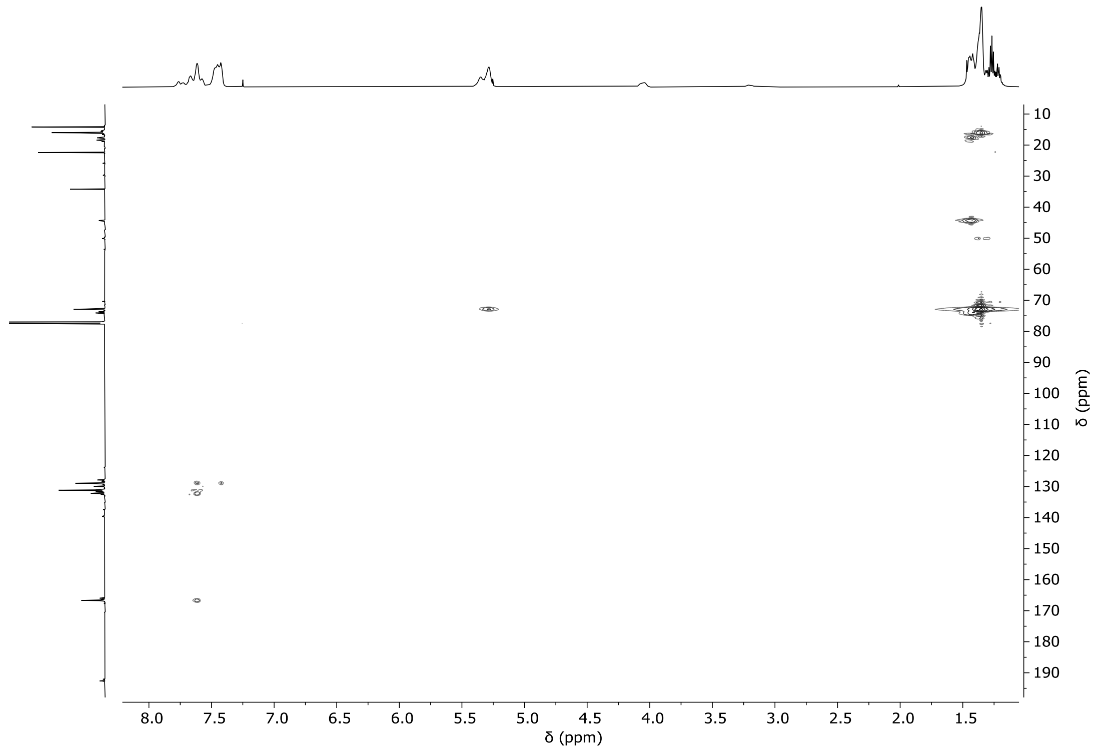

Figure S 28: ^{1}H-^{13}C HMBC NMR spectrum ($CDCl_3$, 25°C) of the precipitated polymer corresponding to table 1, run #3.

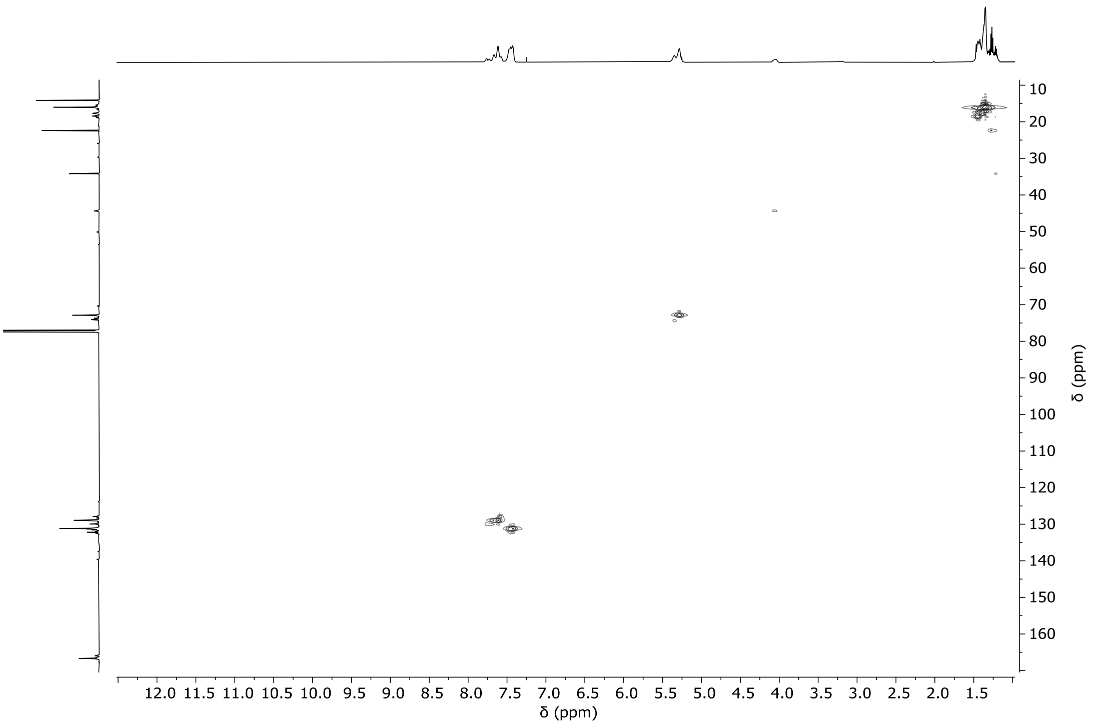

Figure S 29: ^{1}H-^{13}C HMQC NMR spectrum ($CDCl_3$, 25°C) of the precipitated polymer corresponding to table 1, run #3.

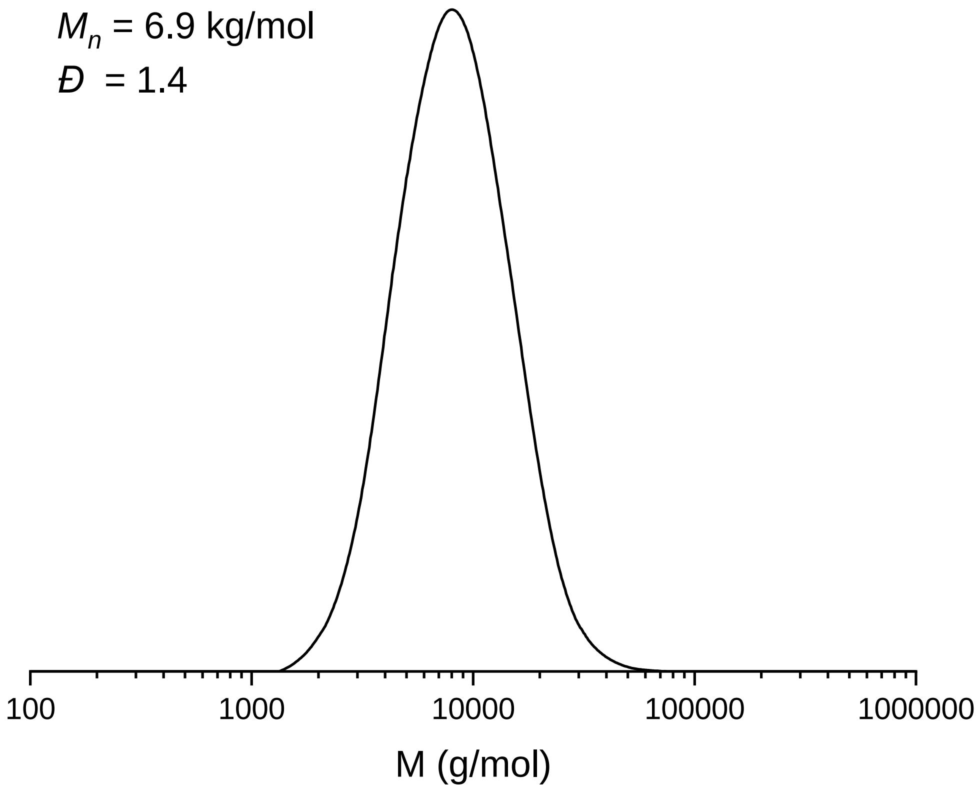

Figure S 30: GPC trace corresponding to table 1, run #3.

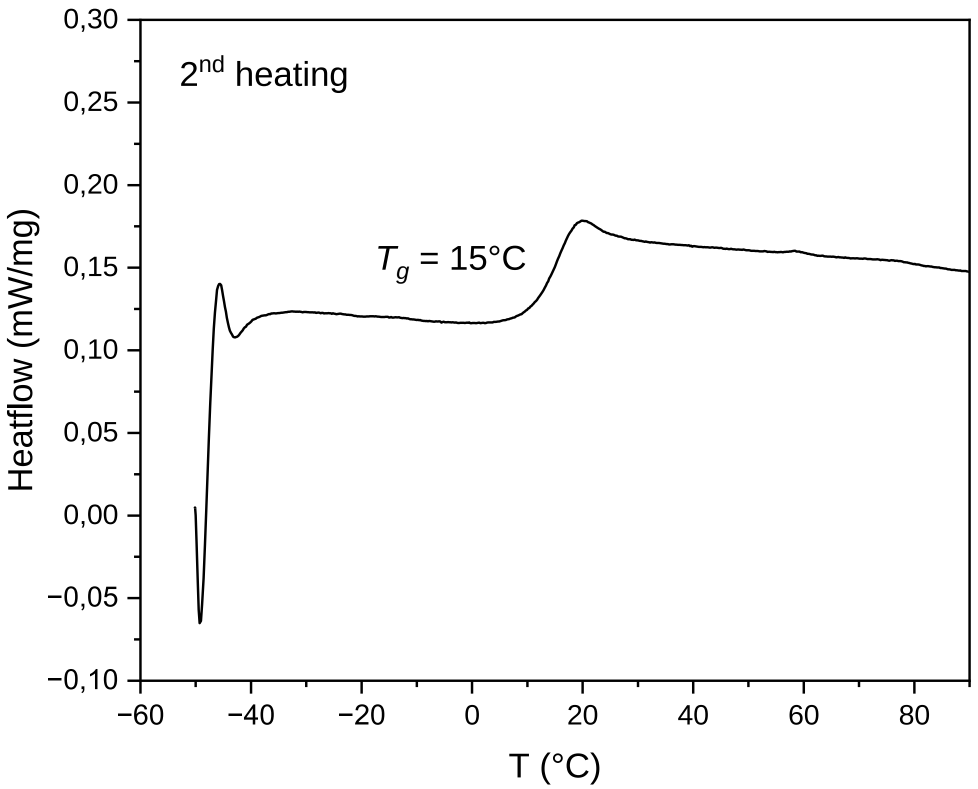

Figure S 31: DSC heating curve of the polymer corresponding table 1, run #3.

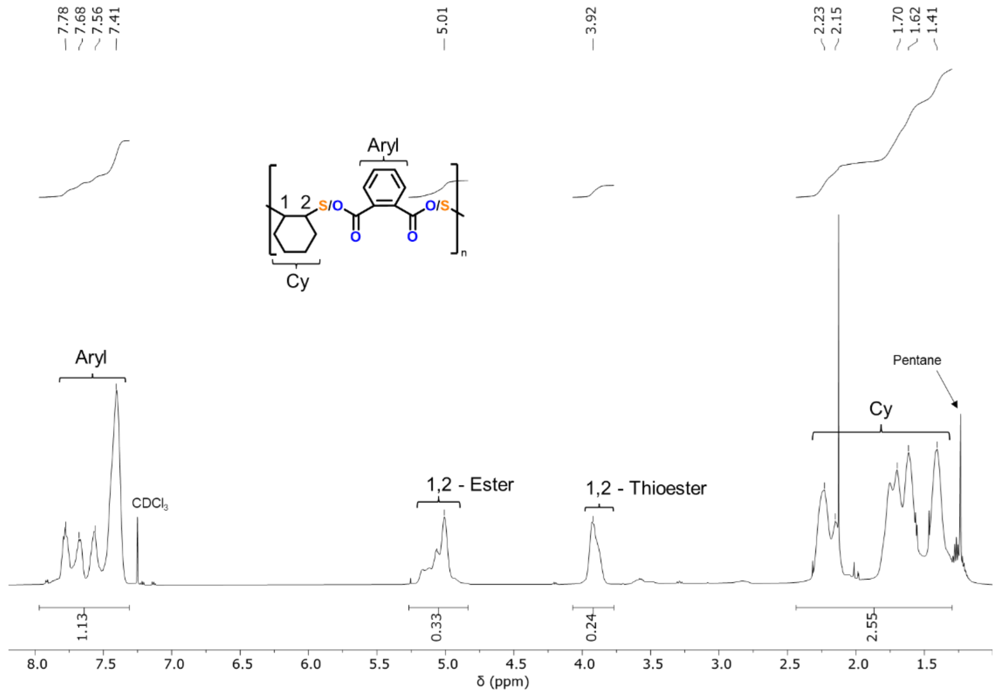

Figure S 32: ^{1}H NMR spectrum (600 MHz, $CDCl_3$, 25°C) of the precipitated polymer corresponding to table 1, run #4.

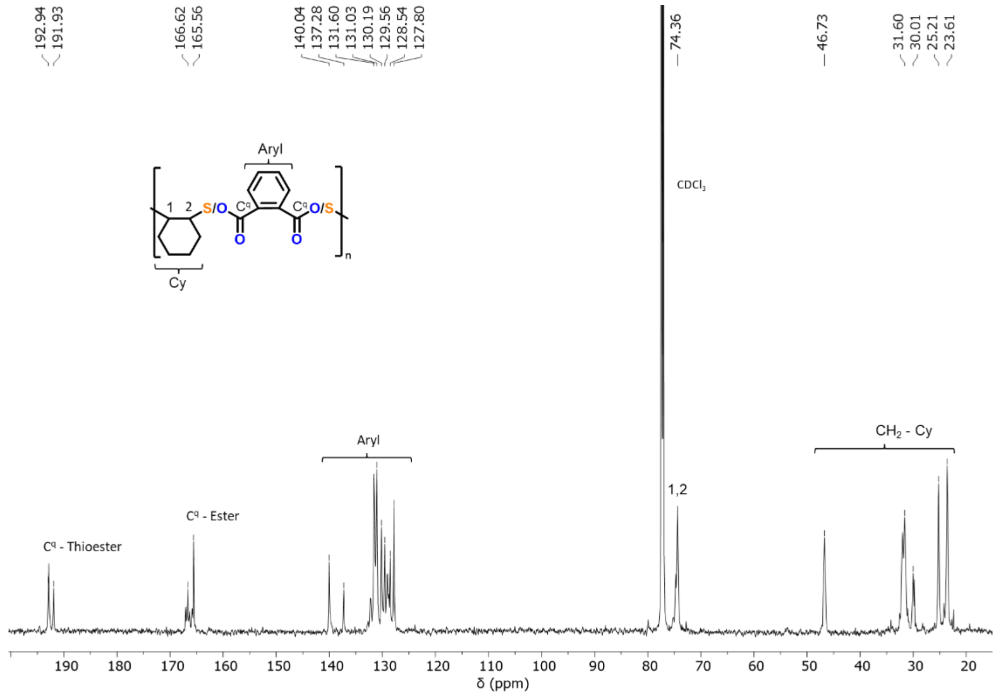

Figure S 33: ^{13}C NMR spectrum (151 MHz, $CDCl_3$, 25°C) of the precipitated polymer corresponding to table 1, run #4 showing an ester-*alt*-thioester selectivity of 56%.

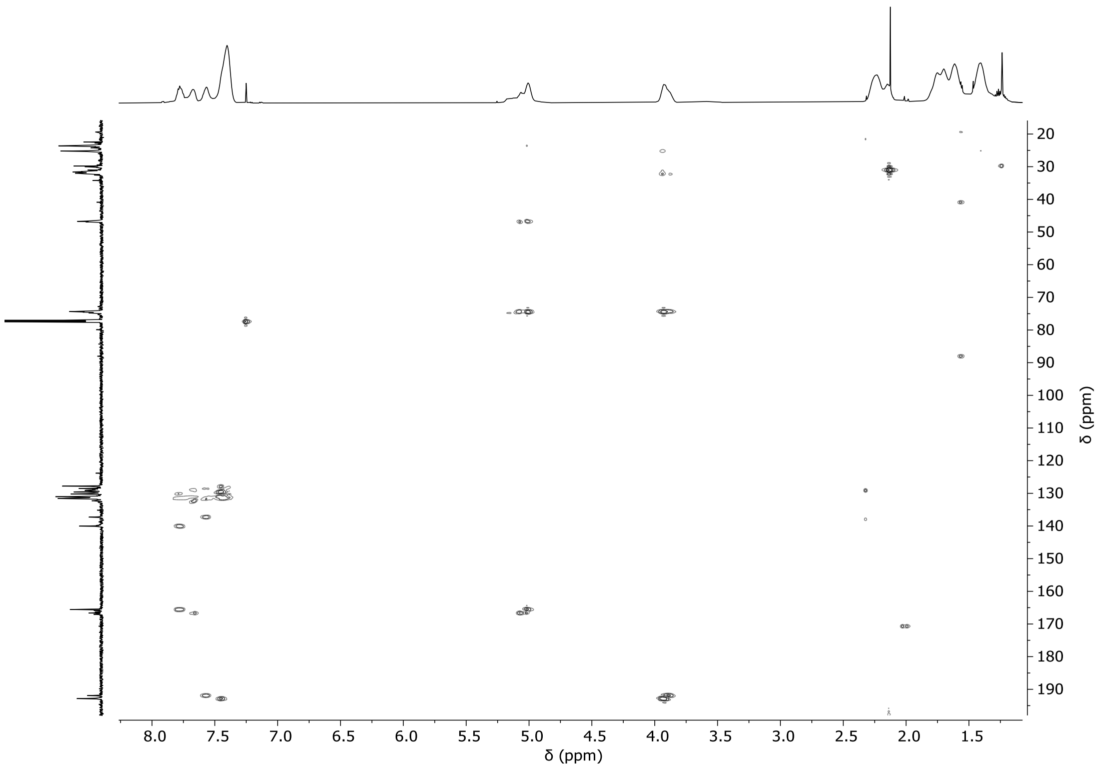

Figure S 34: ^1H-^{13}C HMBC NMR spectrum ($CDCl_3$, 25°C) of the precipitated polymer corresponding to table 1, run #4.

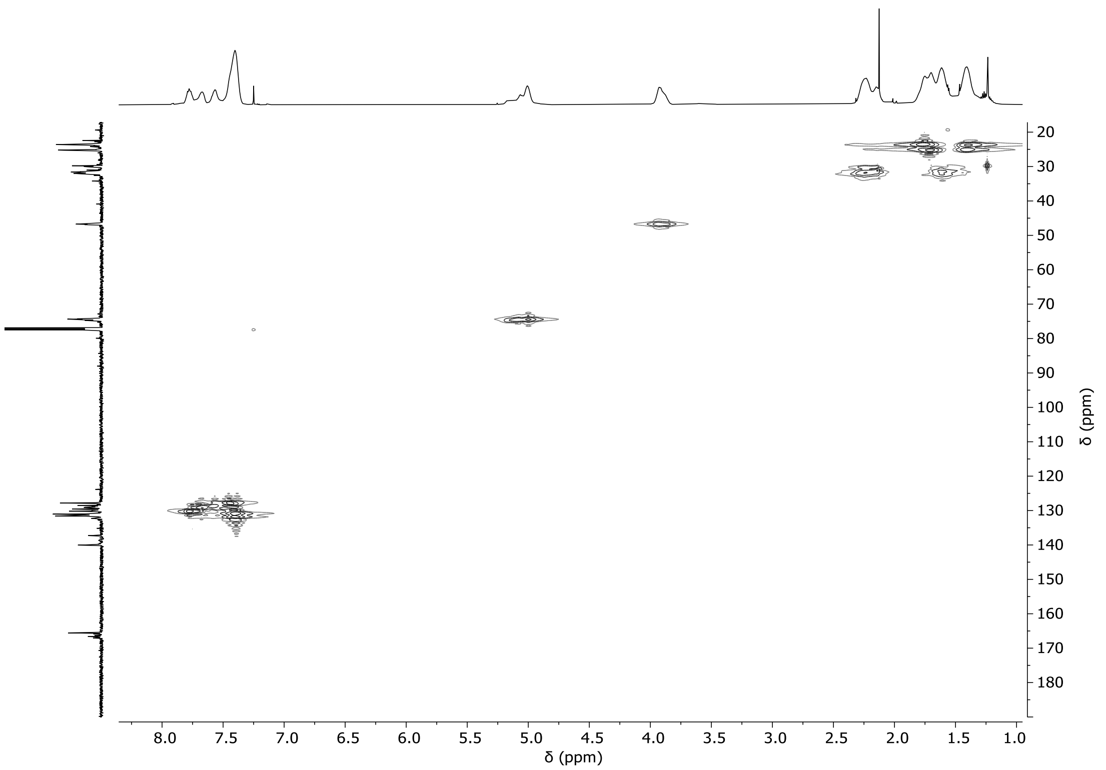

Figure S 35: ^{1}H-^{13}C HMQC NMR spectrum ($CDCl_3$, 25°C) of the precipitated polymer corresponding to table 1, run #4.

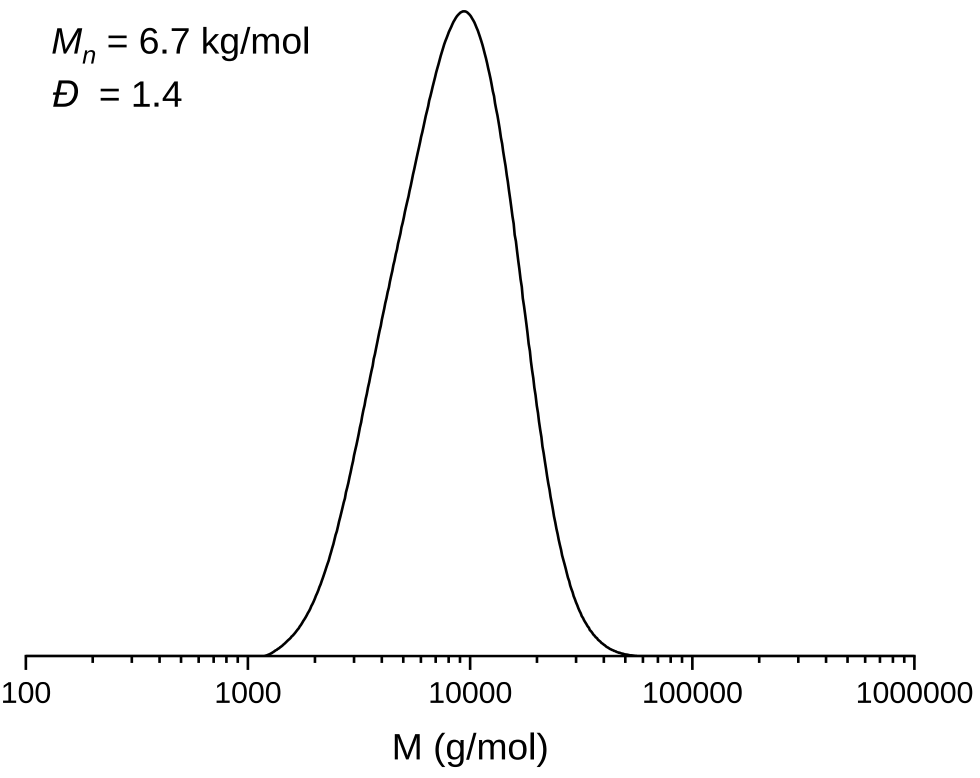

Figure S 36: GPC trace corresponding to table 1, run #4.

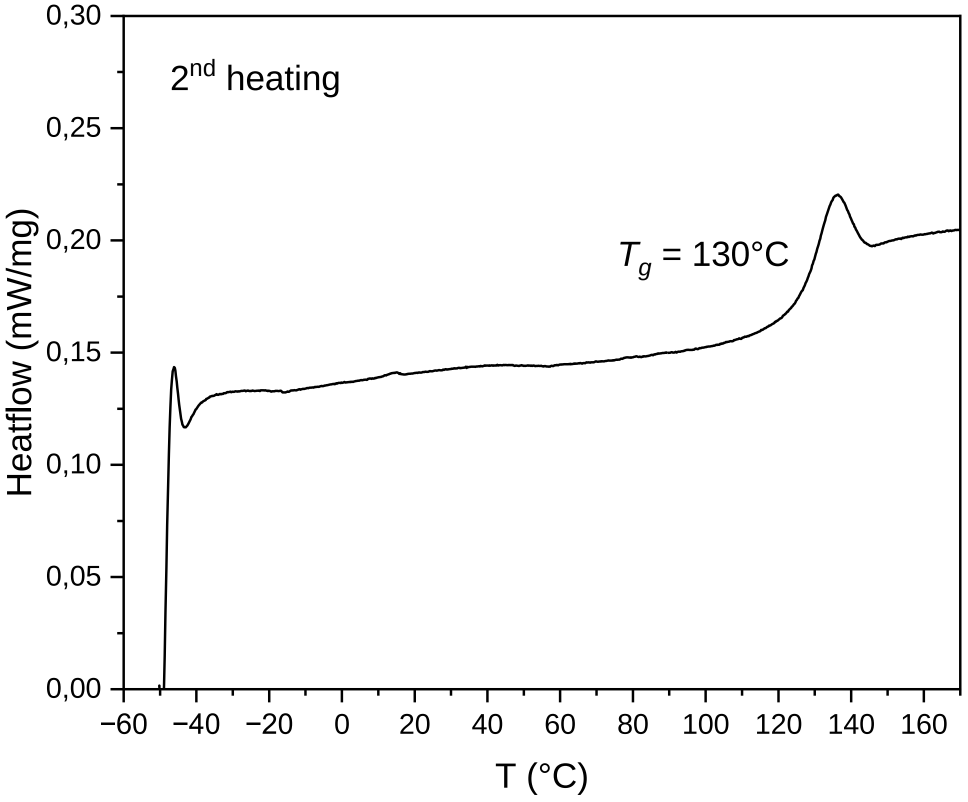

Figure S 37: DSC heating curve of the polymer corresponding table 1, run #4.

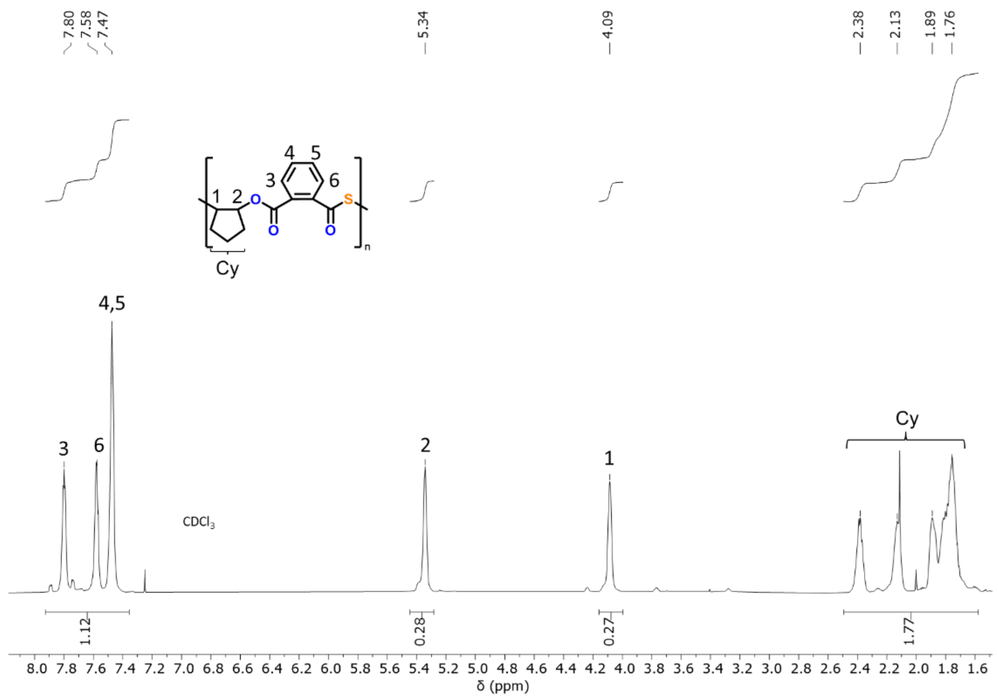

Figure S 38: ^{1}H NMR spectrum (600 MHz, $CDCl_3$, 25°C) of the precipitated polymer corresponding to table 1, run #5.

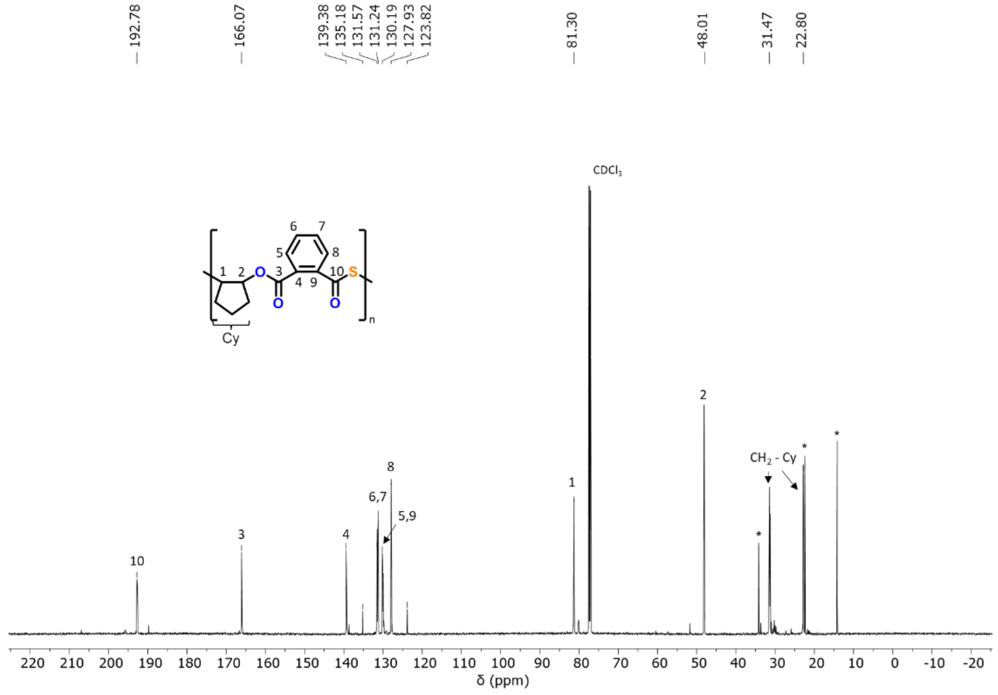

Figure S 39: ^{13}C NMR spectrum (151 MHz, $CDCl_3$, 25°C) of the precipitated polymer corresponding to table 1, run #5. *denotes residual pentane.

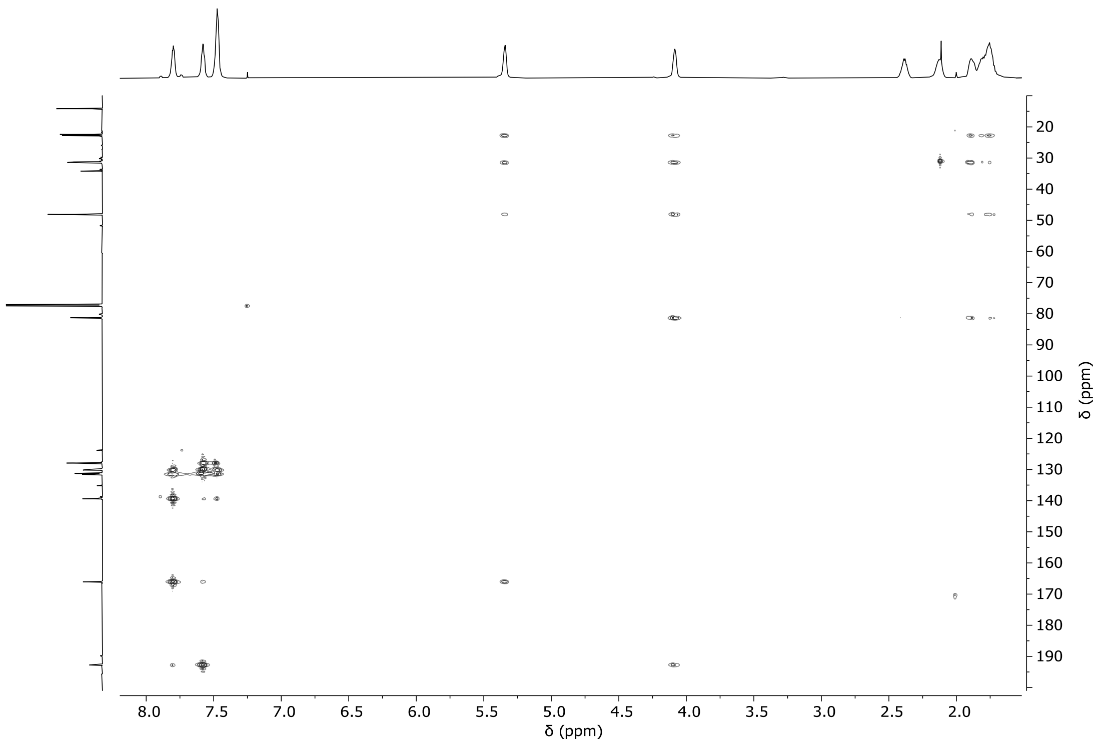

Figure S 40: ^{1}H-^{13}C HMBC NMR spectrum ($CDCl_3$, 25°C) of the precipitated polymer corresponding to table 1, run #5.

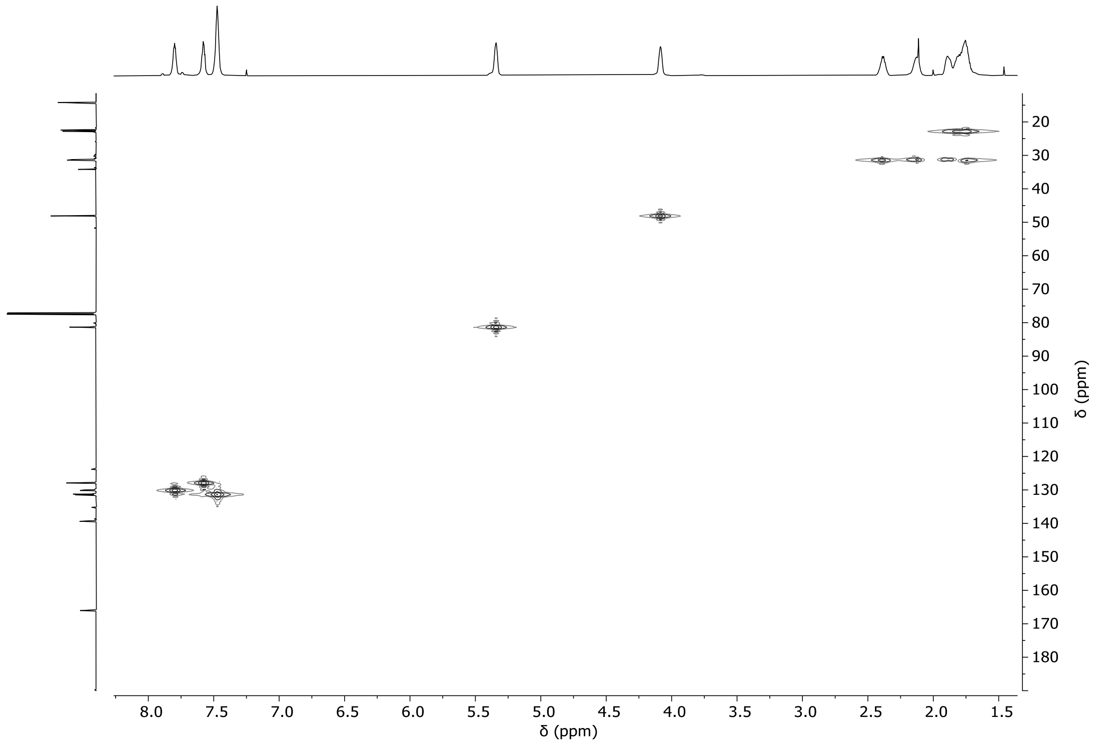

Figure S 41: ^{1}H-^{13}C HMQC NMR spectrum ($CDCl_3$, 25°C) of the precipitated polymer corresponding to table 1, run #5.

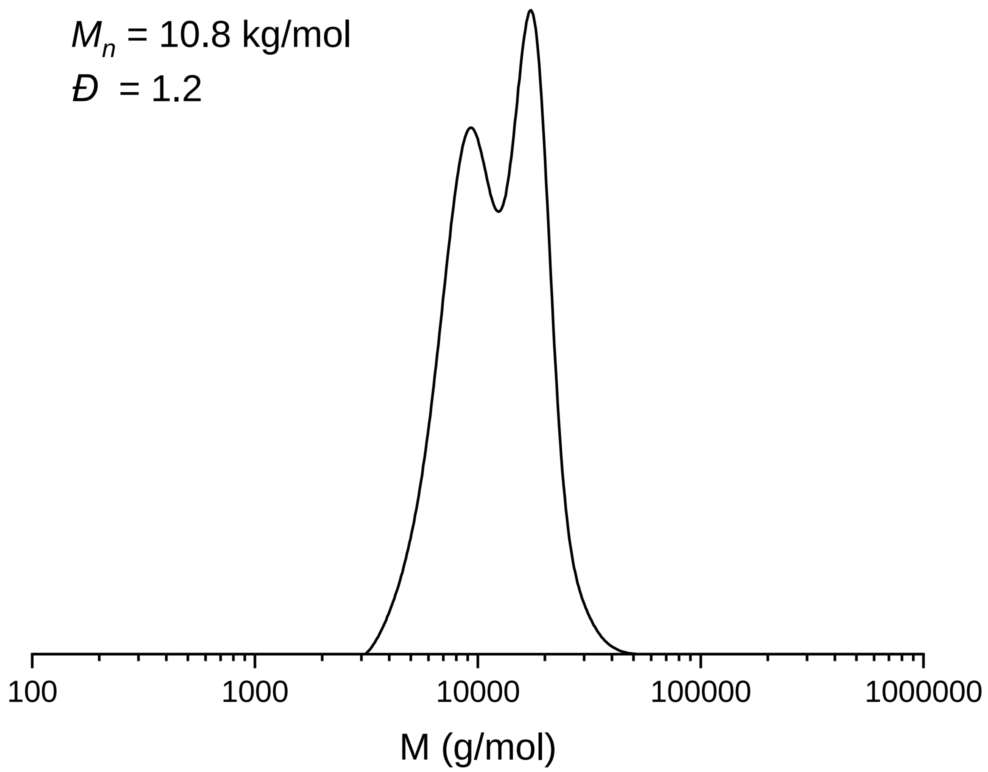

Figure S 42: GPC trace corresponding to table 1, run #5.

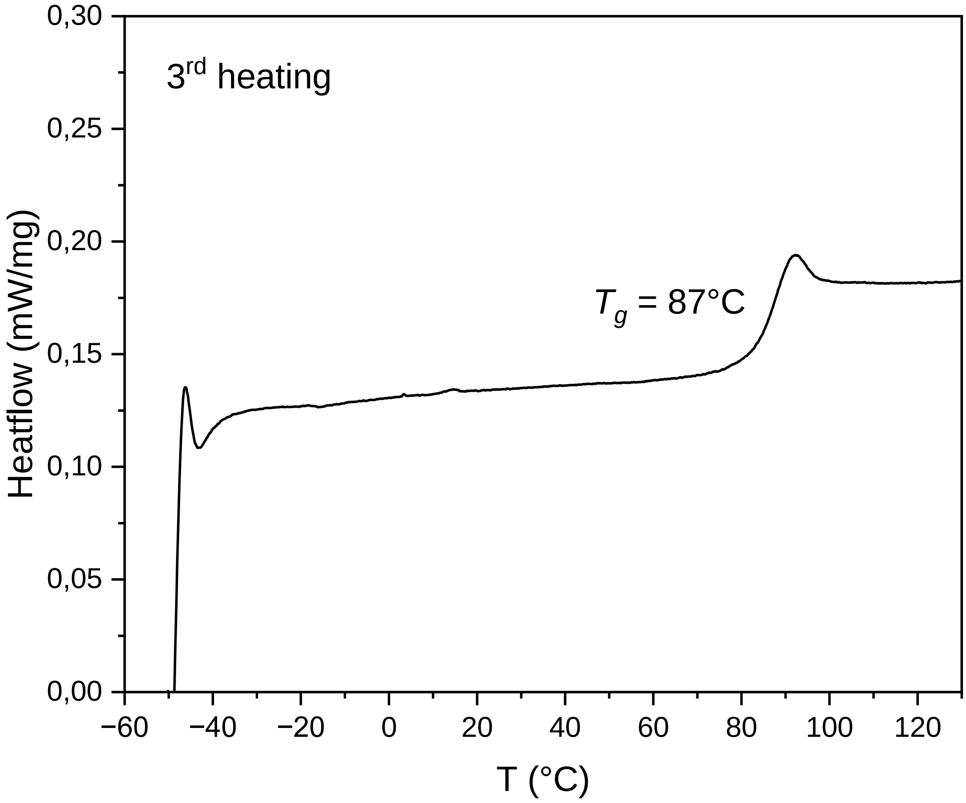

Figure S 43: DSC heating curve of the polymer corresponding table 1, run #5.

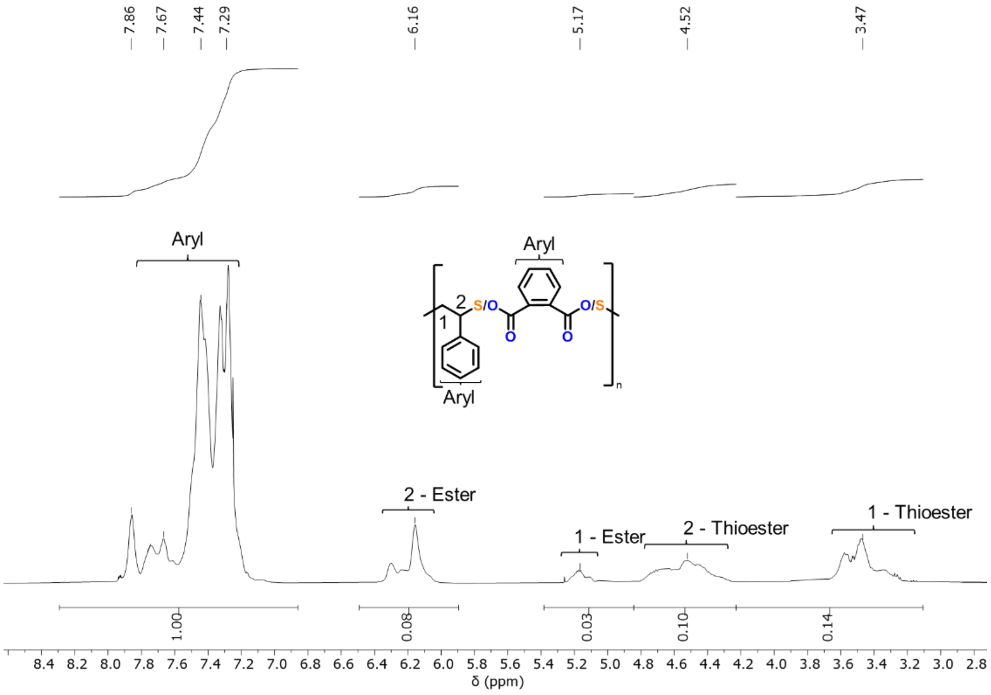

Figure S 44: ^{1}H NMR spectrum (600 MHz, $CDCl_3$, 25°C) of the precipitated polymer corresponding to table 1, run #6.

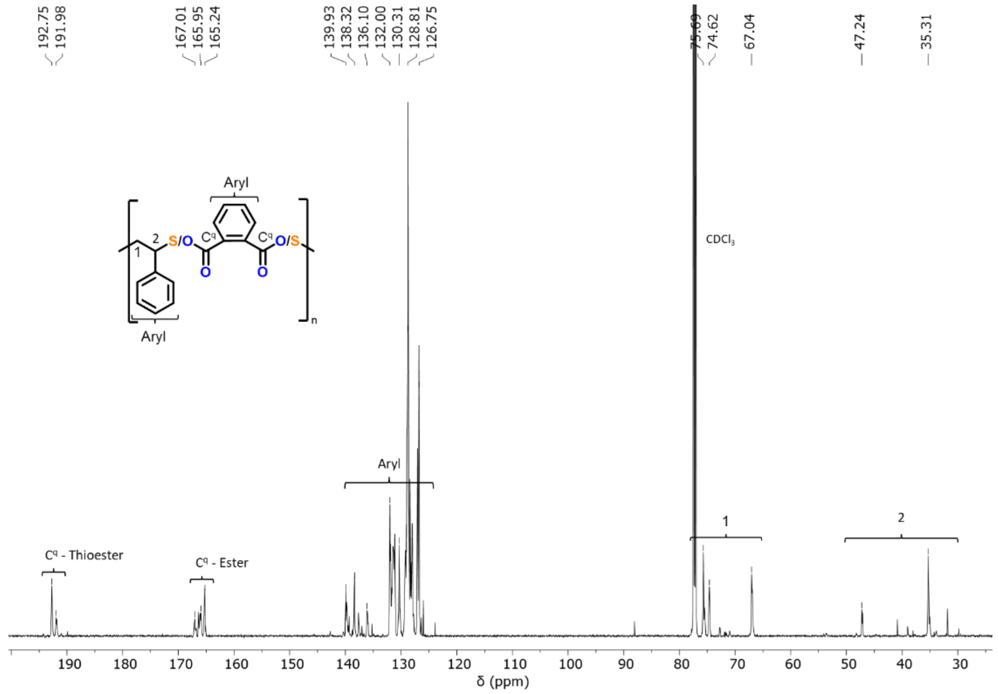

Figure S 45: ^{13}C NMR spectrum (151 MHz, $CDCl_3$, 25°C) of the precipitated polymer corresponding to table 1, run #6.

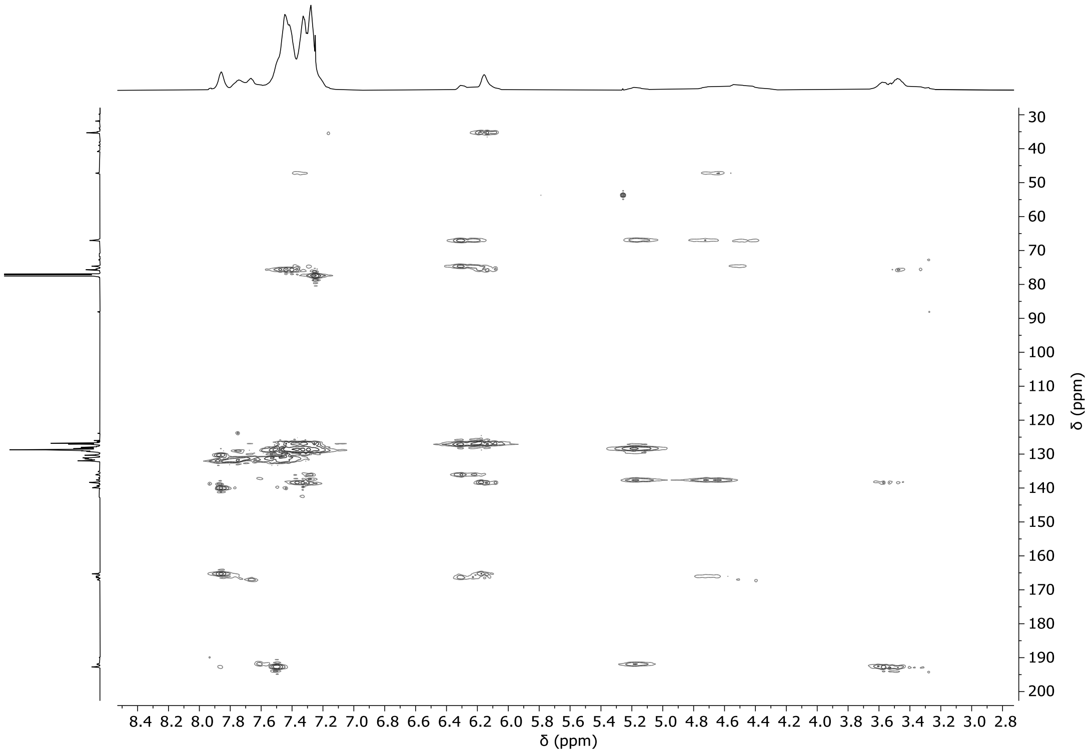

Figure S 46: ^{1}H-^{13}C HMBC NMR spectrum ($CDCl_3$, 25°C) of the precipitated polymer corresponding to table 1, run #6.

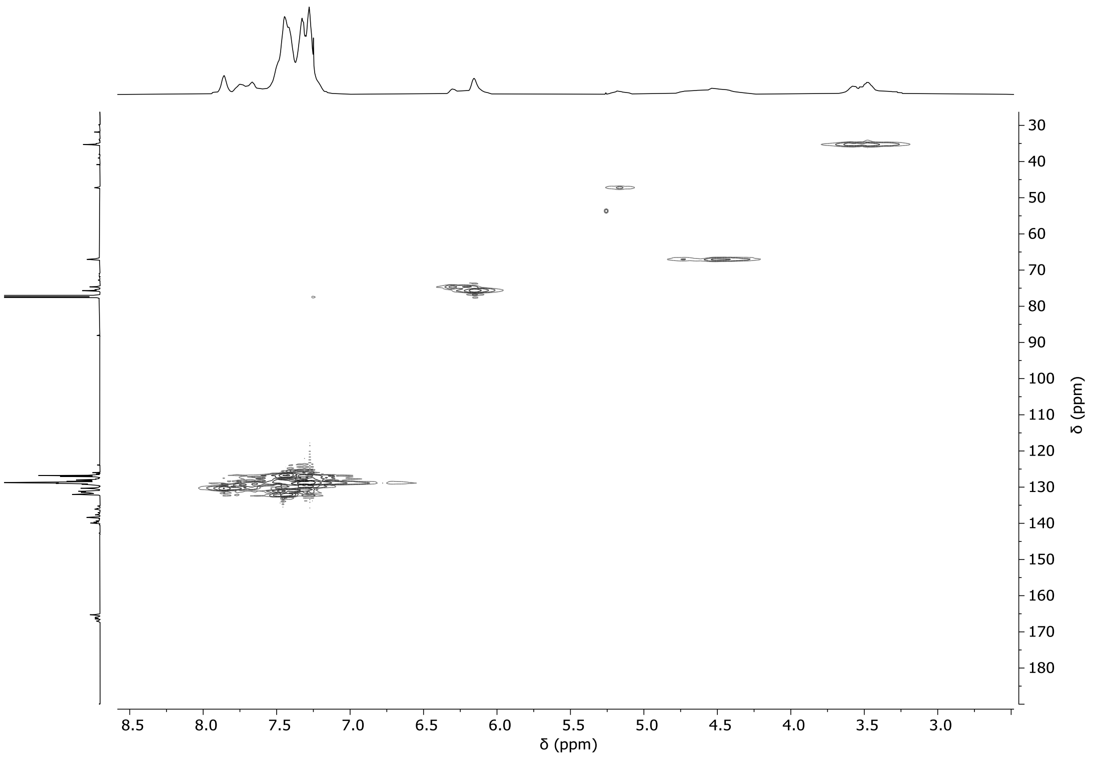

Figure S 47: ^{1}H-^{13}C HMQC NMR spectrum ($CDCl_3$, 25°C) of the precipitated polymer corresponding to table 1, run #6.

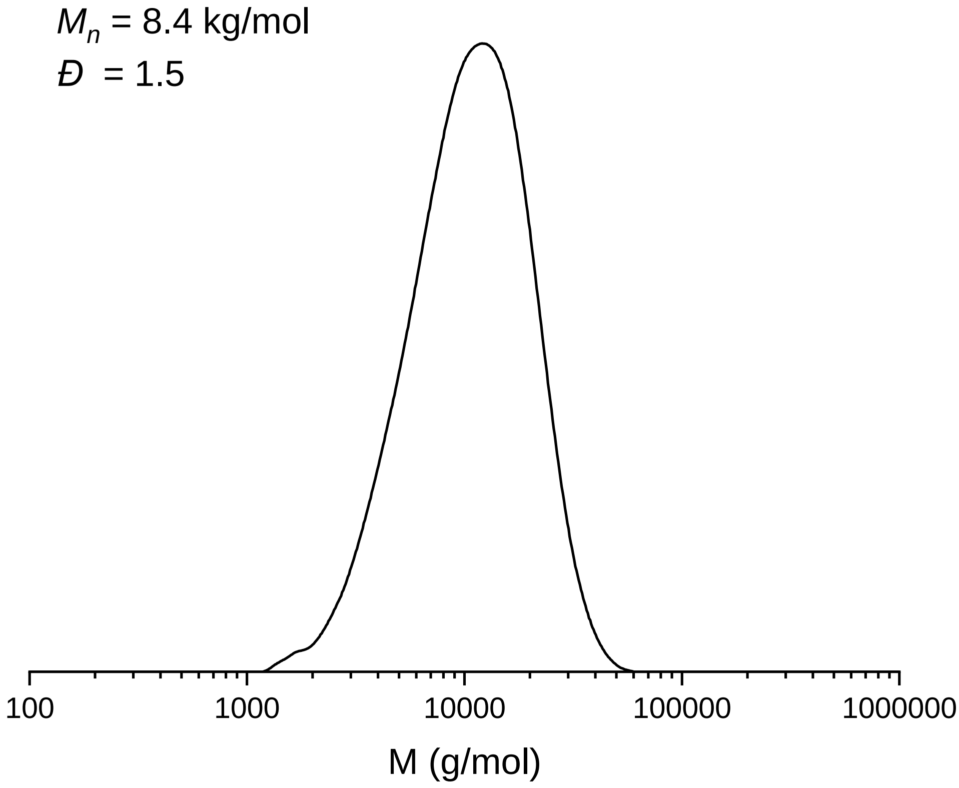

Figure S 48: GPC trace corresponding to table 1, run #6.

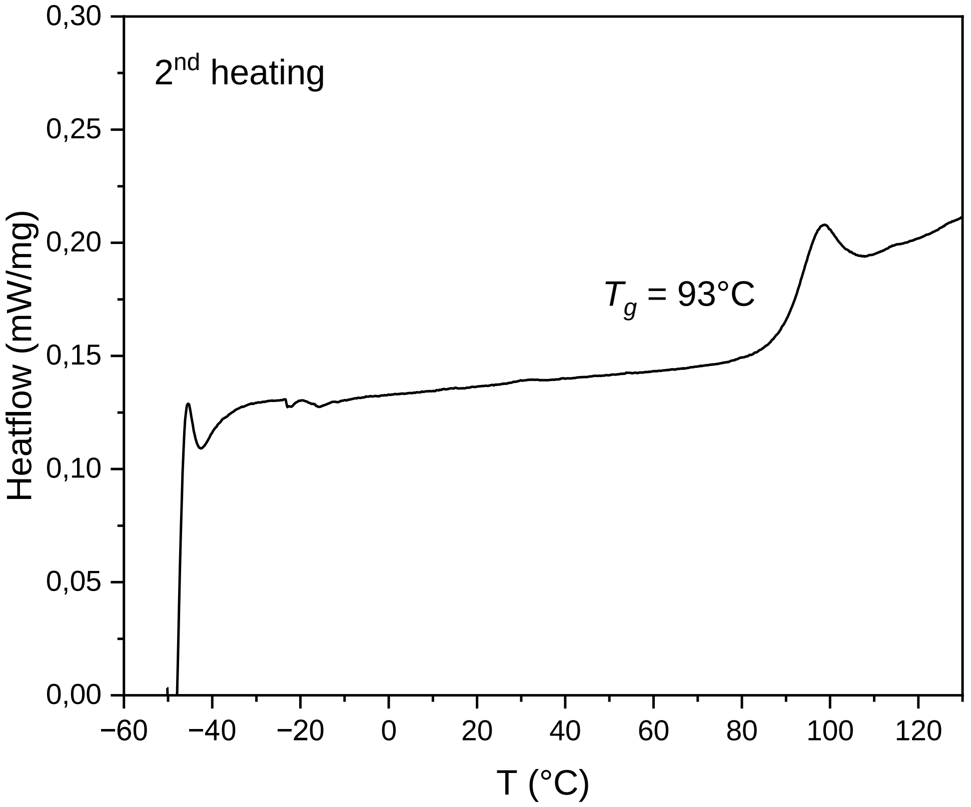

Figure S 49: DSC heating curve of the polymer corresponding table 1, run #6.

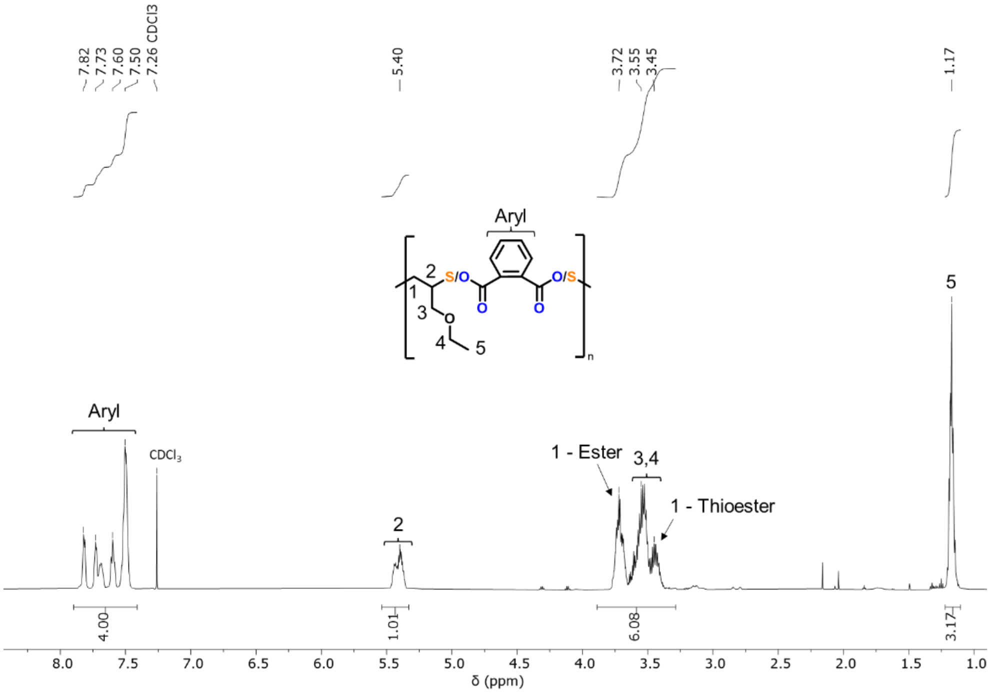

Figure S 50: ^{1}H NMR spectrum (600 MHz, $CDCl_3$, 25°C) of the precipitated polymer corresponding to table 1, run #7.

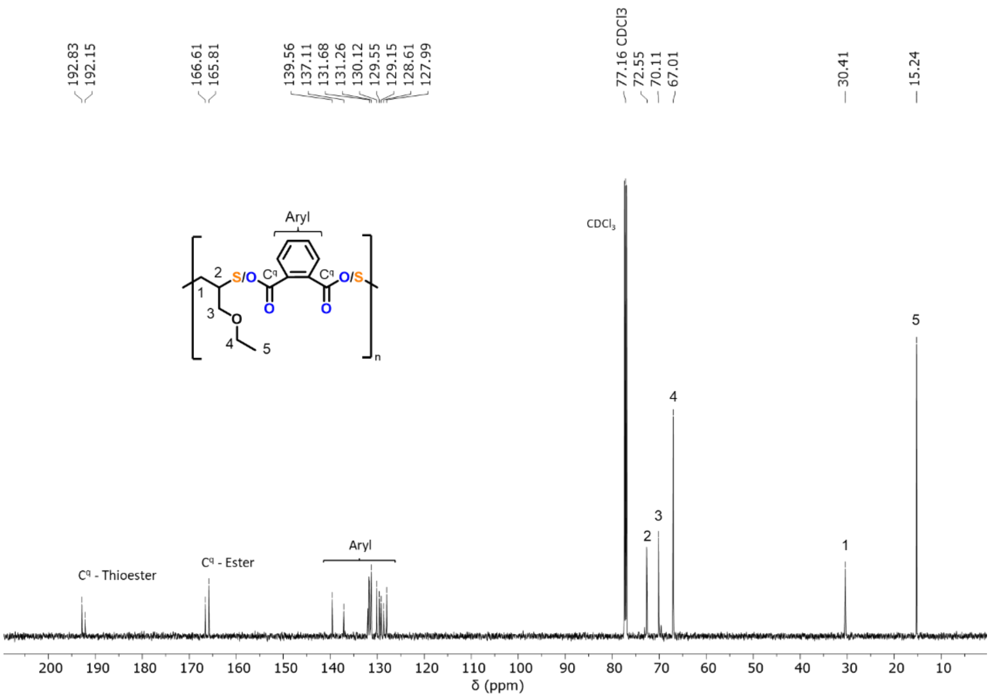

Figure S 51: ^{13}C NMR spectrum (151 MHz, $CDCl_3$, 25°C) of the precipitated polymer corresponding to table 1, run #7 showing an ester-*alt*-thioester selectivity of 61%.

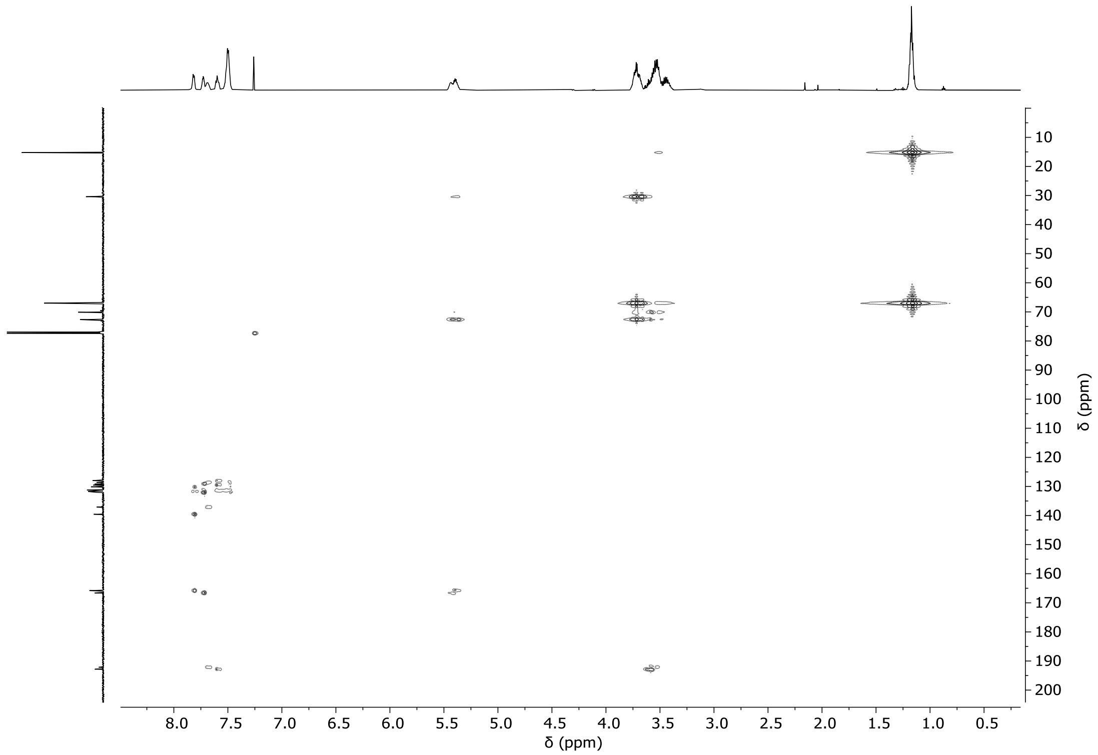

Figure S 52: ^{1}H-^{13}C HMBC NMR spectrum ($CDCl_3$, 25°C) of the precipitated polymer corresponding to table 1, run #7.

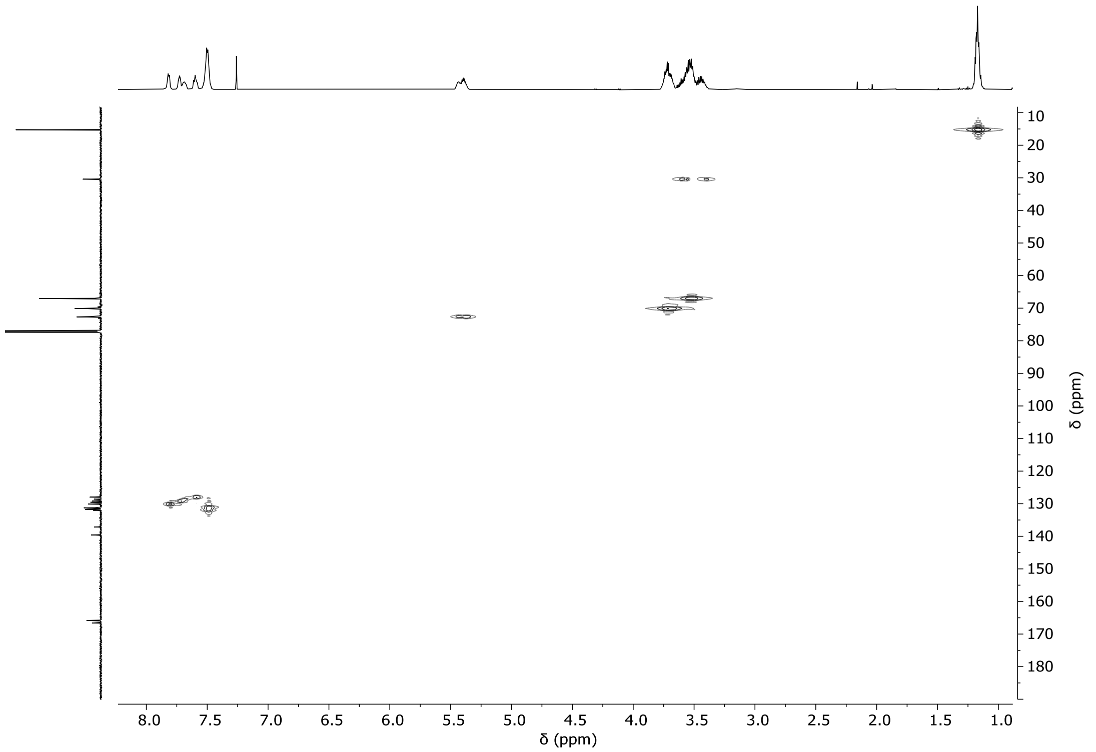

Figure S 53: ^1H-^{13}C HMQC NMR spectrum ($CDCl_3$, 25°C) of the precipitated polymer corresponding to table 1, run #7.

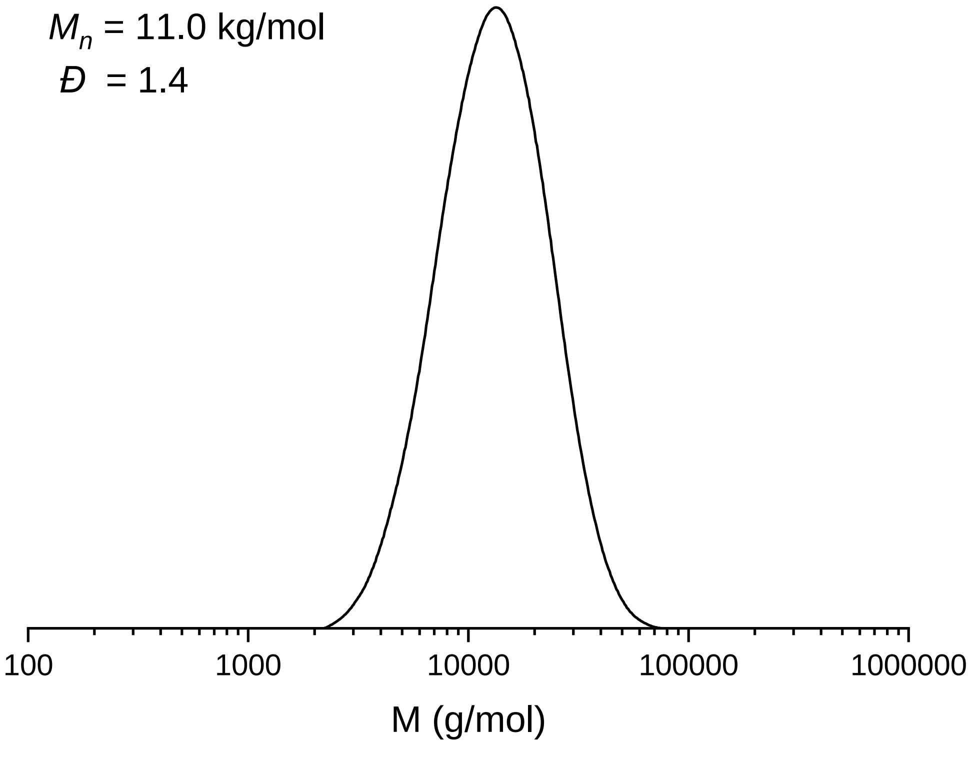

Figure S 54: GPC trace corresponding to table 1, run #7.

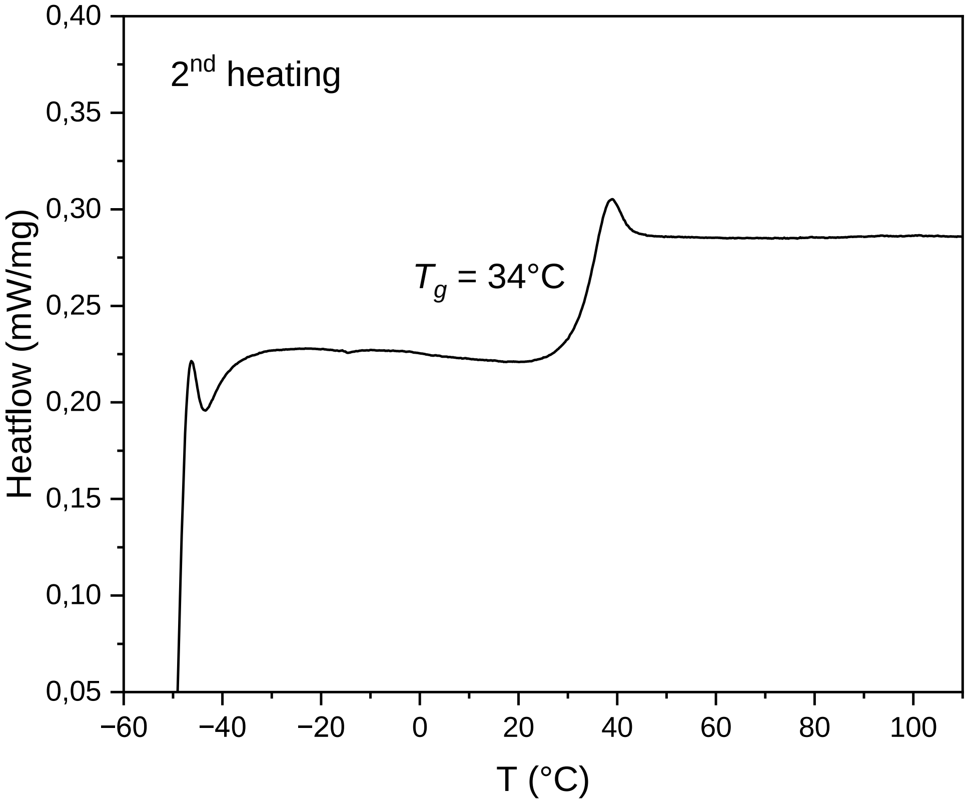

Figure S 55: DSC heating curve of the polymer corresponding table 1, run #7.

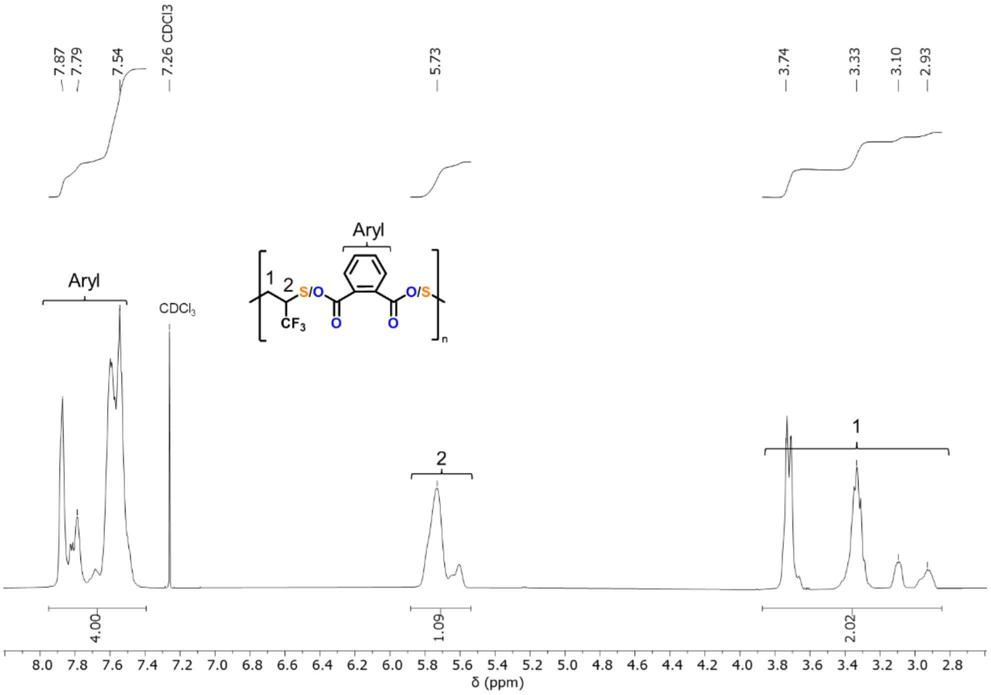

Figure S 56: ^{1}H NMR spectrum (600 MHz, $CDCl_3$, 25°C) of the precipitated polymer corresponding to table 1, run #8.

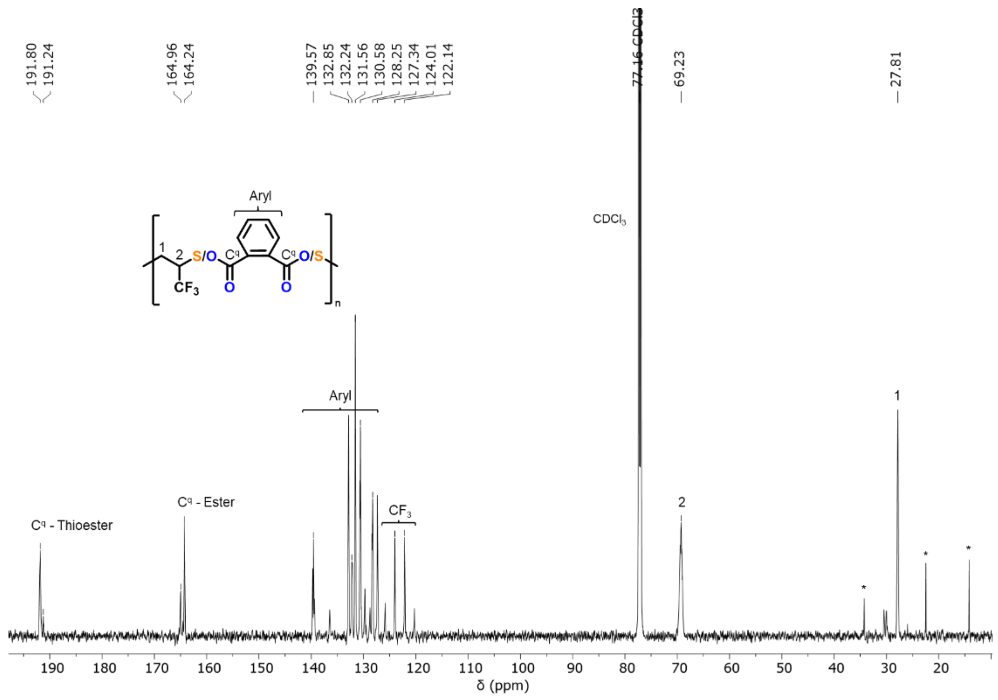

Figure S 57: ^{13}C NMR spectrum (151 MHz, $CDCl_3$, 25°C) of the precipitated polymer corresponding to table 1, run #8. *denotes residual pentane.

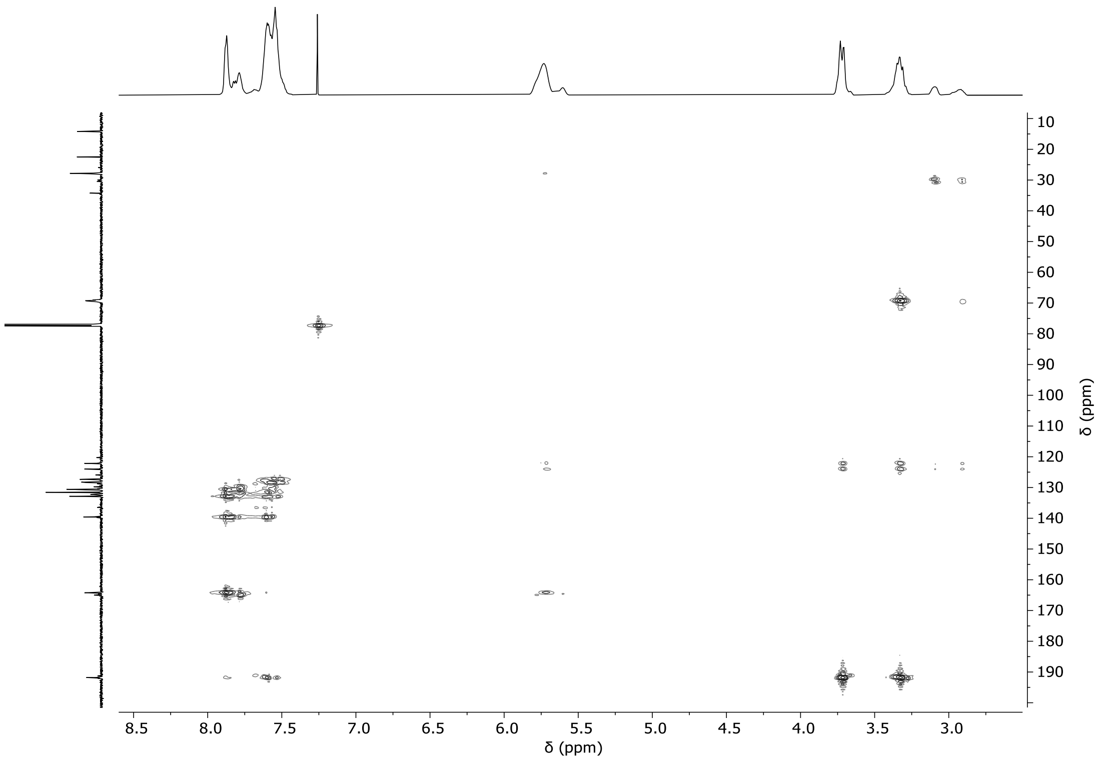

Figure S 58: ^{1}H-^{13}C HMBC NMR spectrum ($CDCl_3$, 25°C) of the precipitated polymer corresponding to table 1, run #8.

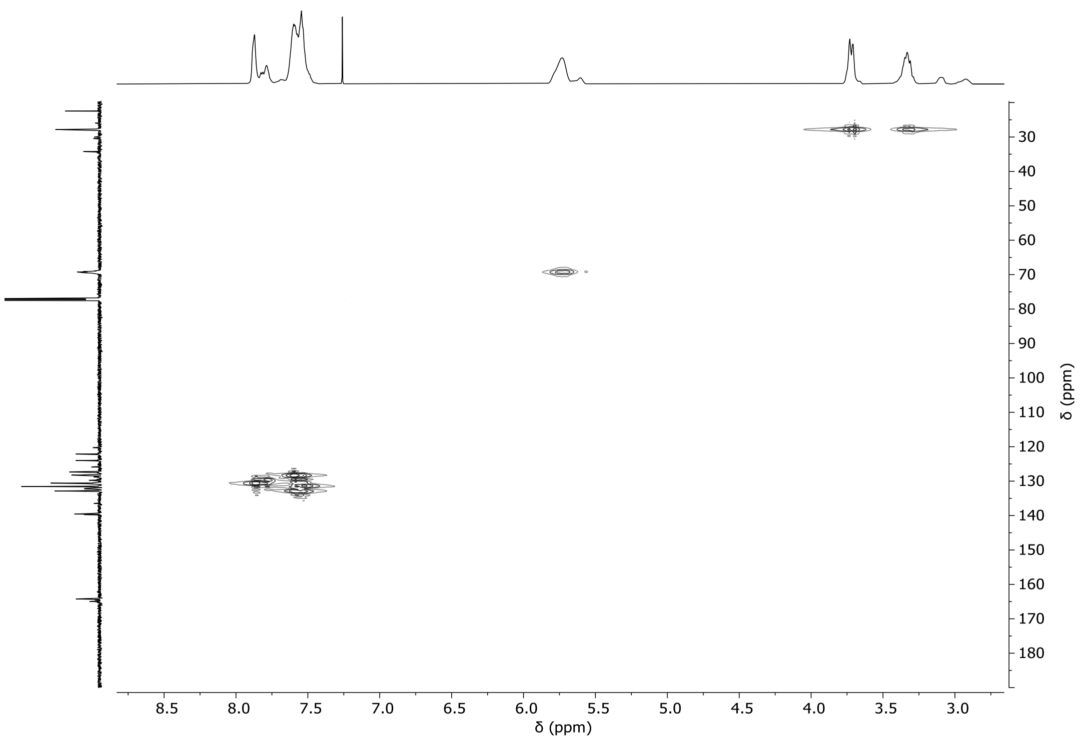

Figure S 59: ^{1}H-^{13}C HMQC NMR spectrum ($CDCl_3$, 25°C) of the precipitated polymer corresponding to table 1, run #8.

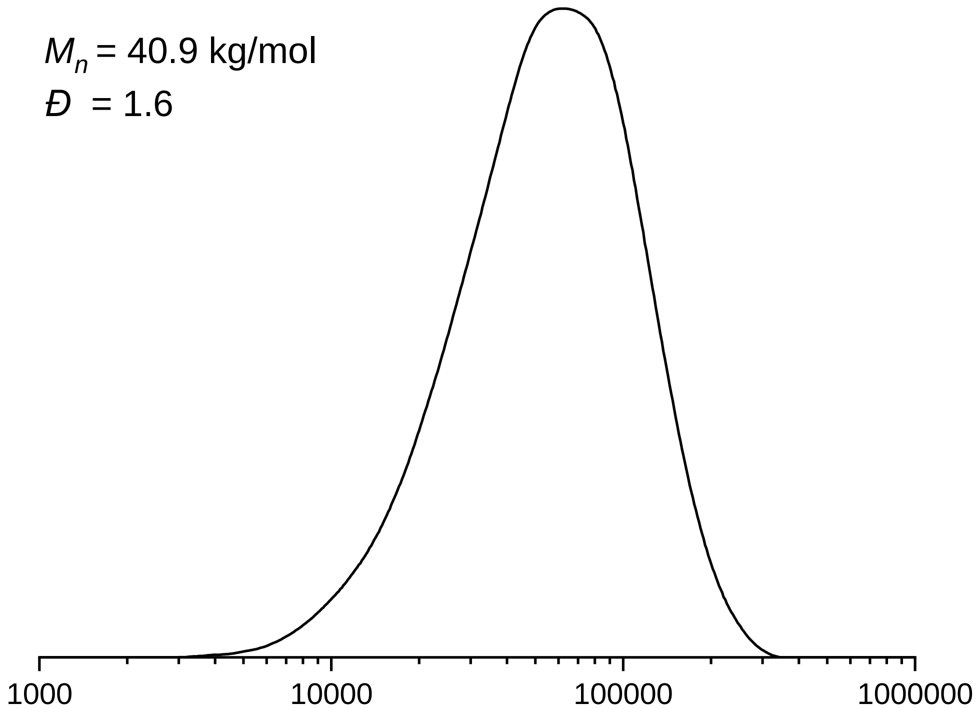

Figure S 60: GPC trace corresponding to table 1, run #8.

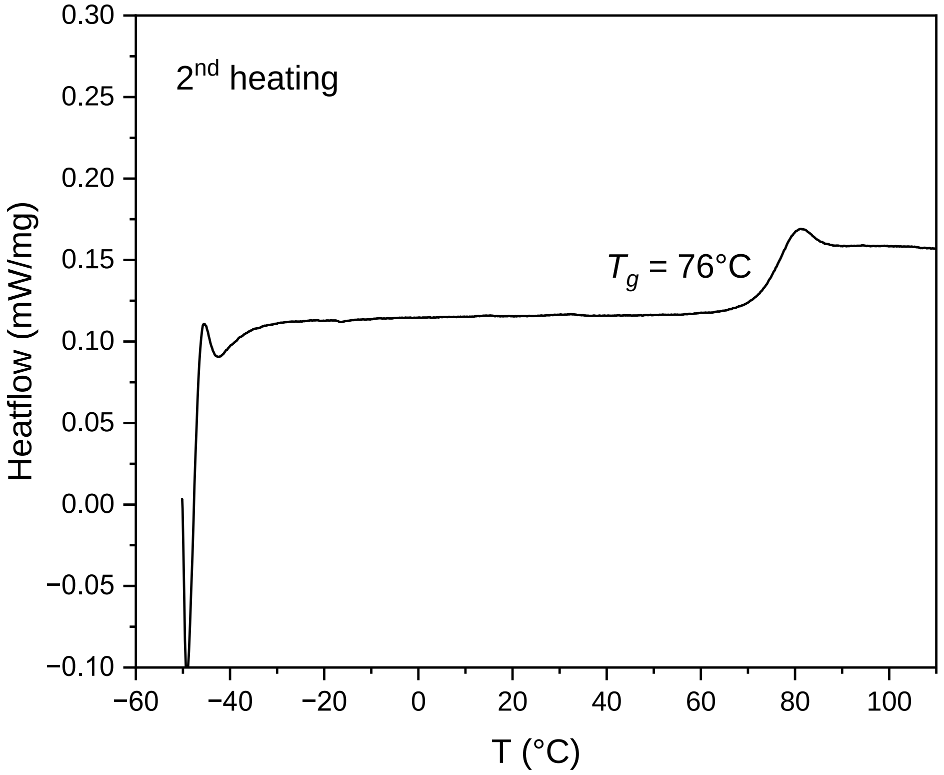

Figure S 61: DSC heating curve of the polymer corresponding table 1, run #8.

Section S4: Thiirane/PTA Copolymerisations

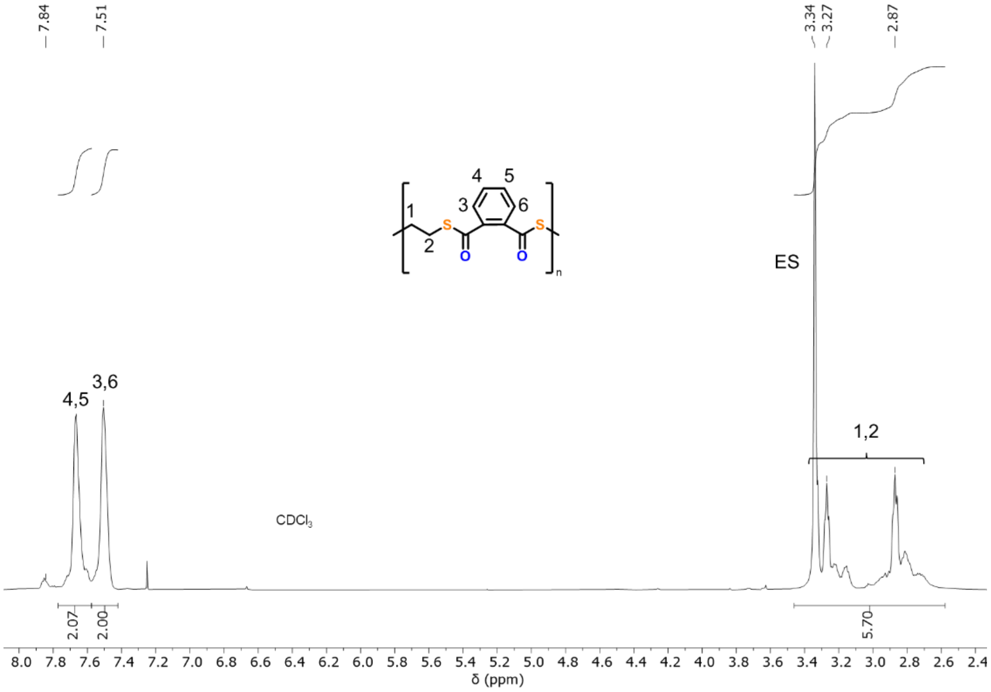

Figure S 62: ^{1}H NMR spectrum (600 MHz, $CDCl_3$, 25°C) of the precipitated polymer obtained from ethylene sulfide/PTA ROCOP.

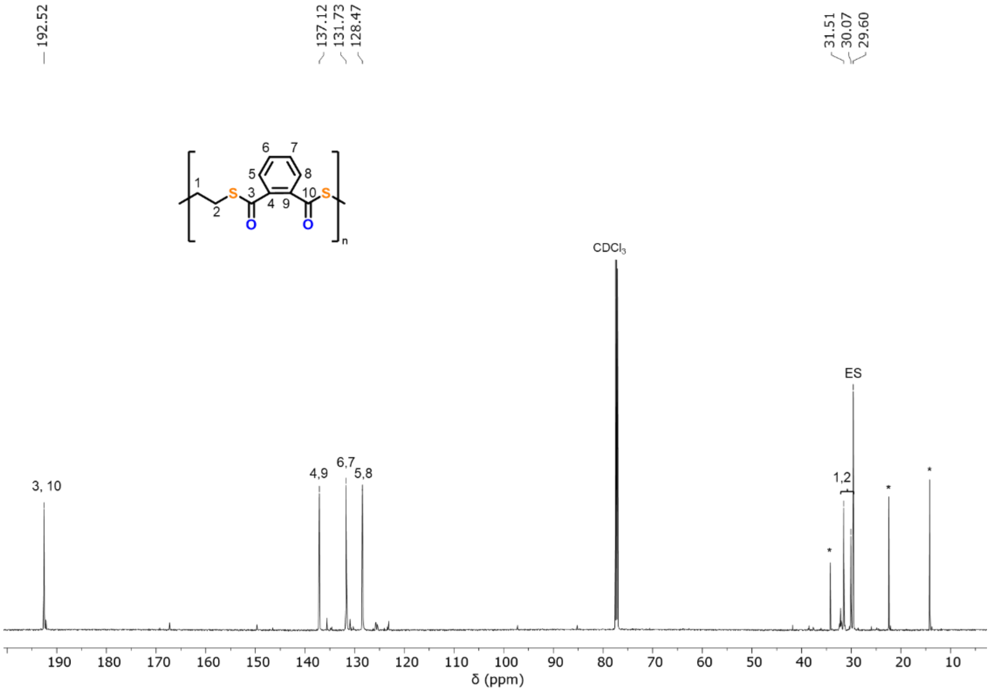

Figure S 63: ^{13}C NMR spectrum (151 MHz, $CDCl_3$, 25°C) of the precipitated polymer obtained from ethylene sulfide/PTA ROCOP. *denotes residual pentane.

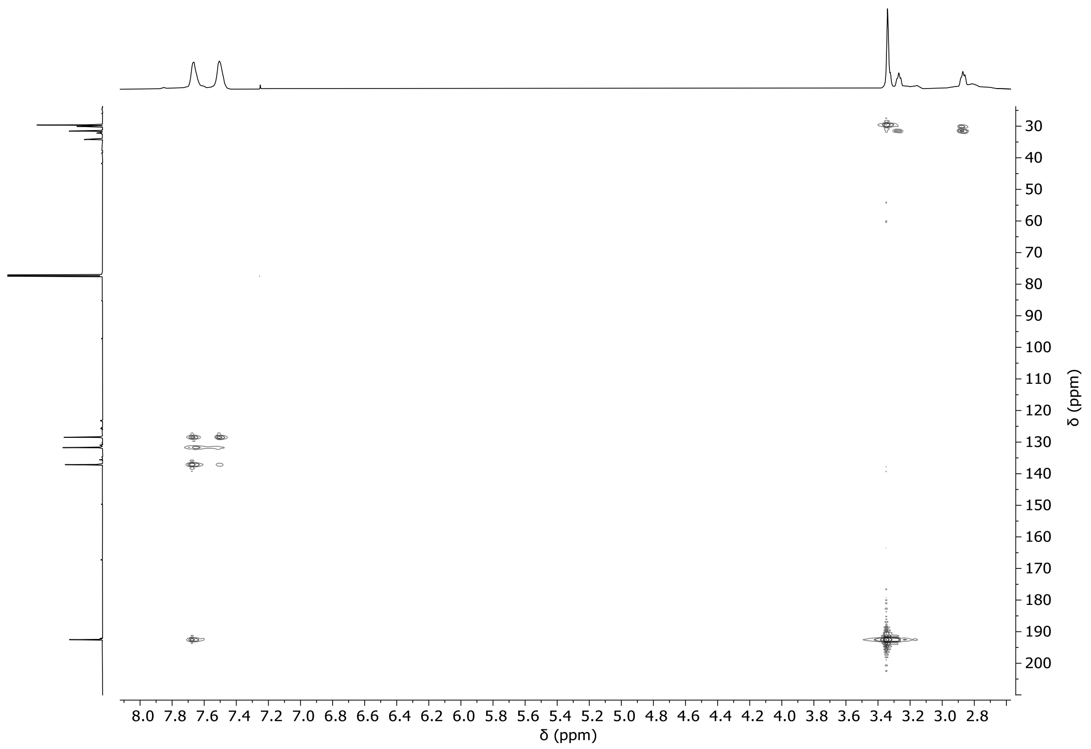

Figure S 64: ^{1}H-^{13}C HMBC NMR spectrum ($CDCl_3$, 25°C) of the precipitated polymer obtained from ethylene sulfide/PTA ROCOP.

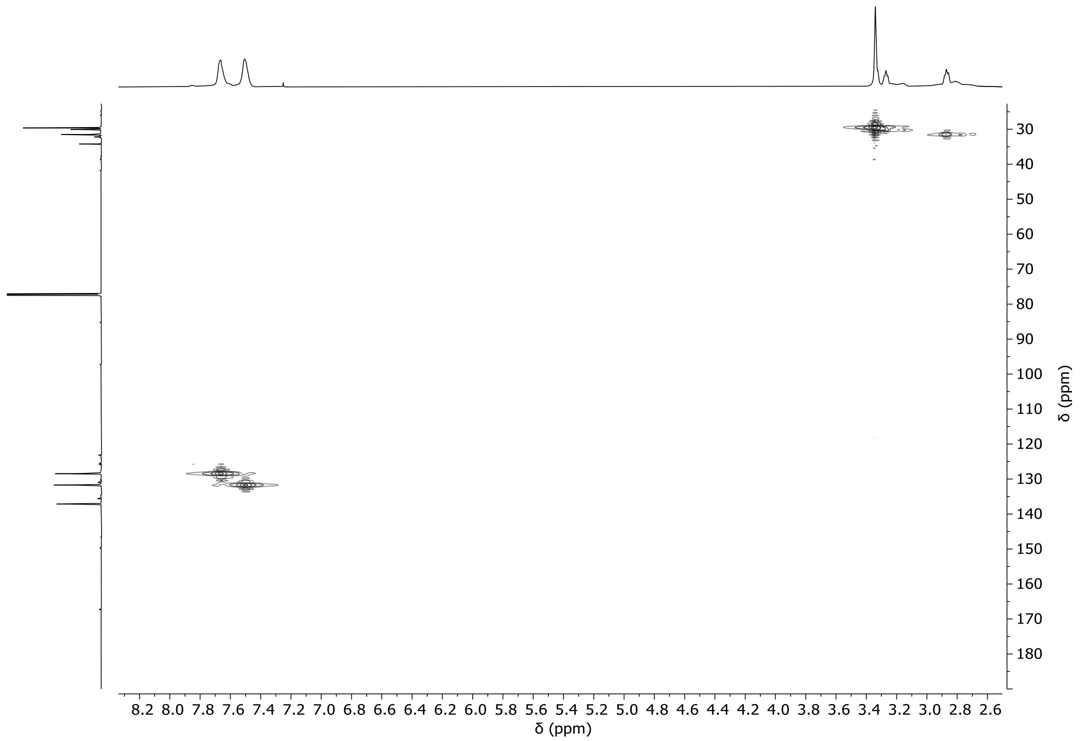

Figure S 65: ^{1}H-^{13}C HMQC NMR spectrum ($CDCl_3$, 25°C) of the precipitated polymer obtained from ethylene sulfide/PTA ROCOP.

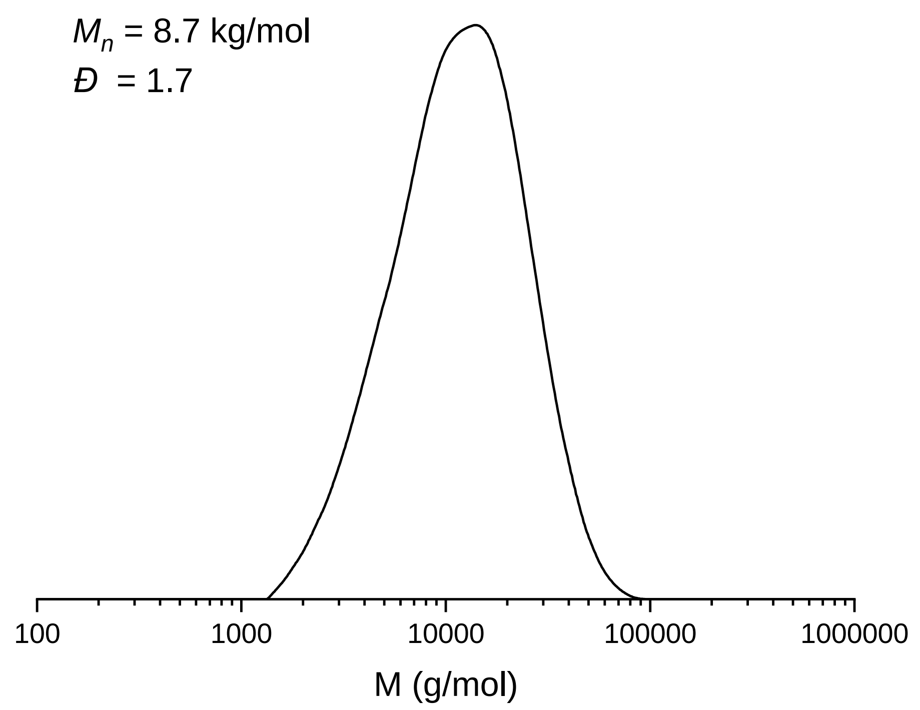

Figure S 66: GPC trace of the polymer obtained from ethylene sulfide/PTA ROCOP.

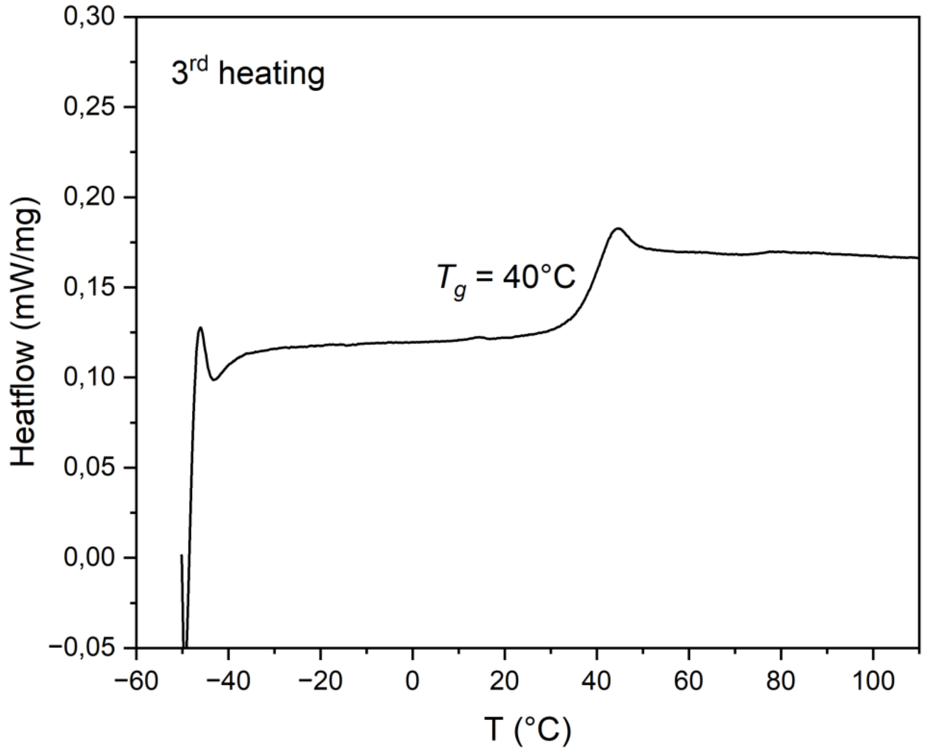

Figure S 67: DSC heating curve of the polymer obtained from ethylene sulfide/PTA ROCOP.

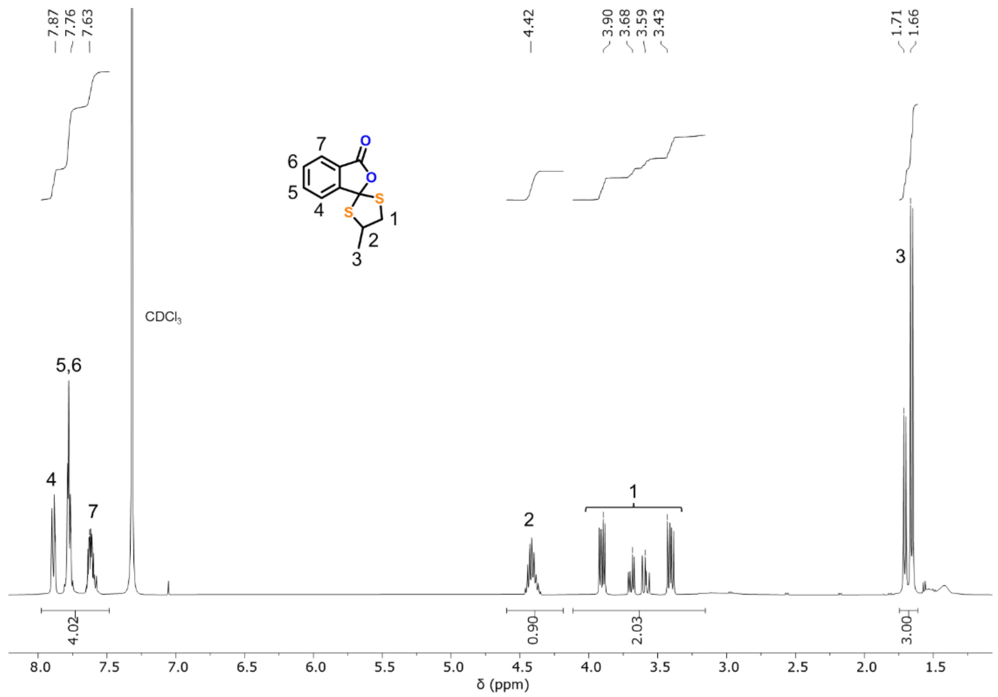

Figure S 68: ^{1}H NMR spectrum (600 MHz, $CDCl_3$, 25°C) of the cyclic product obtained from propylene sulfide/PTA ROCOP.

Section S5: Oxetane/PTA Copolymerisations

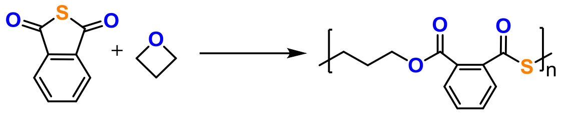

Scheme 1: Synthesis of PTA/OX copolymer.

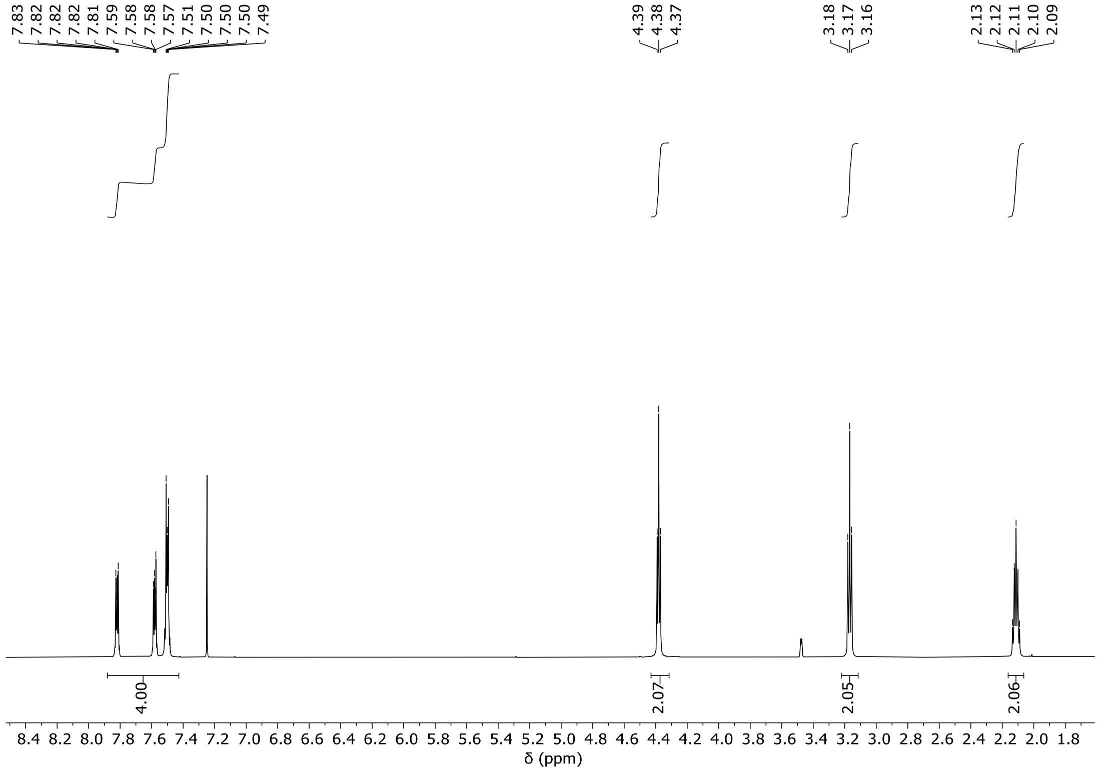

Figure S 69: ^{1}H NMR spectrum (400 MHz, $CDCl_3$, 25°C) of the polymer corresponding to table 1, run #0 showing a selective poly(ester-*alt*-thioester) formation; residual MeOH at ca. 3.4 ppm.

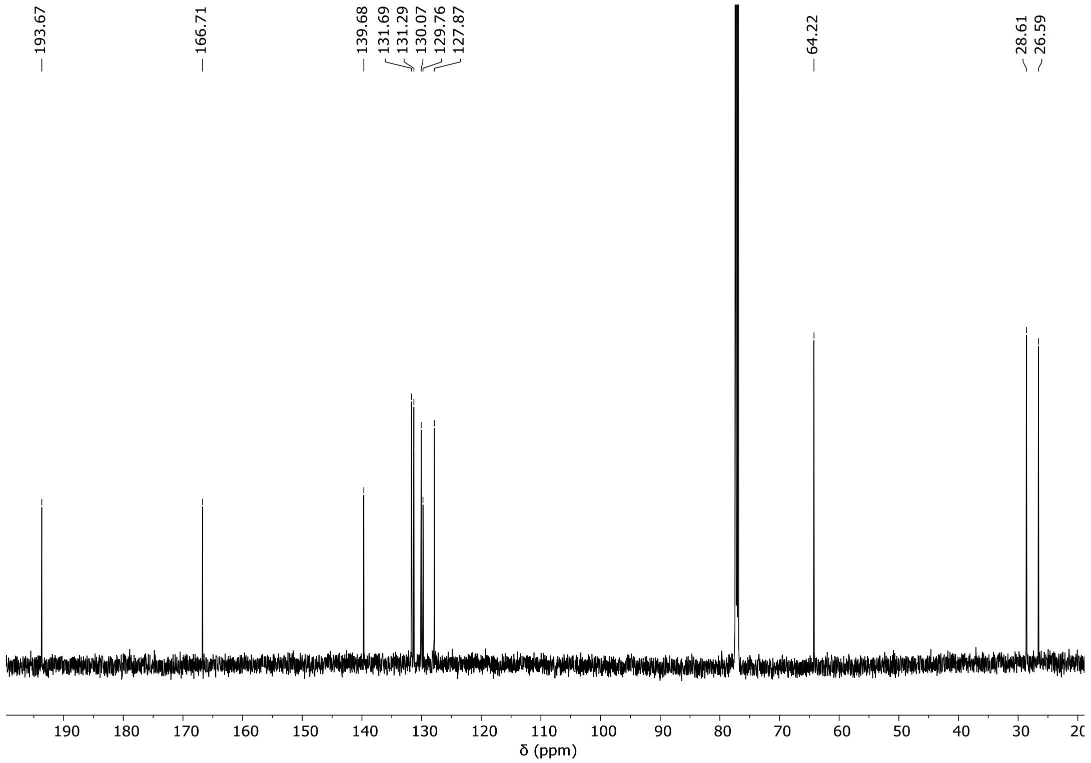

Figure S 70: ^{13}C NMR spectrum (126 MHz, $CDCl_3$, 25°C) spectrum of the polymer corresponding to table 1, run #0.

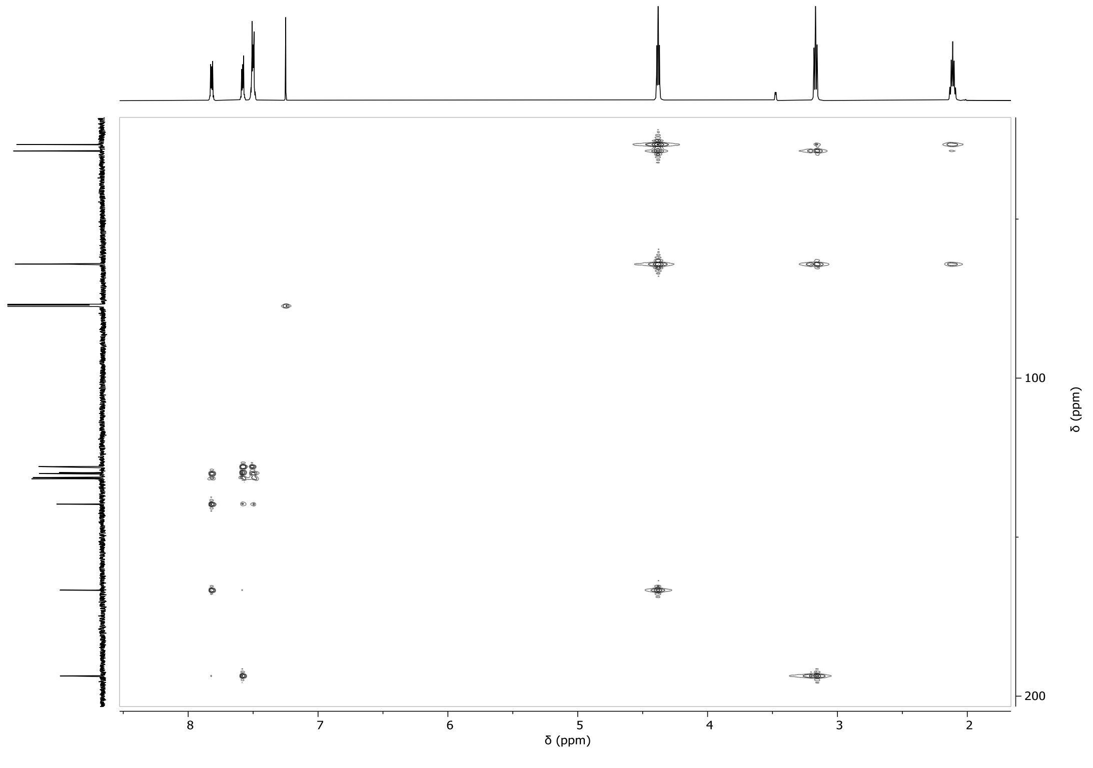

Figure S 71: ^{1}H-^{13}C HMBC NMR spectrum ($CDCl_3$, 25°C) spectrum of the polymer corresponding to table 1, run #0.

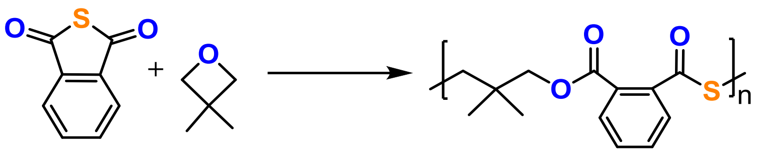

Scheme 2: Synthesis of CS_2/OX^{Me} copolymer.

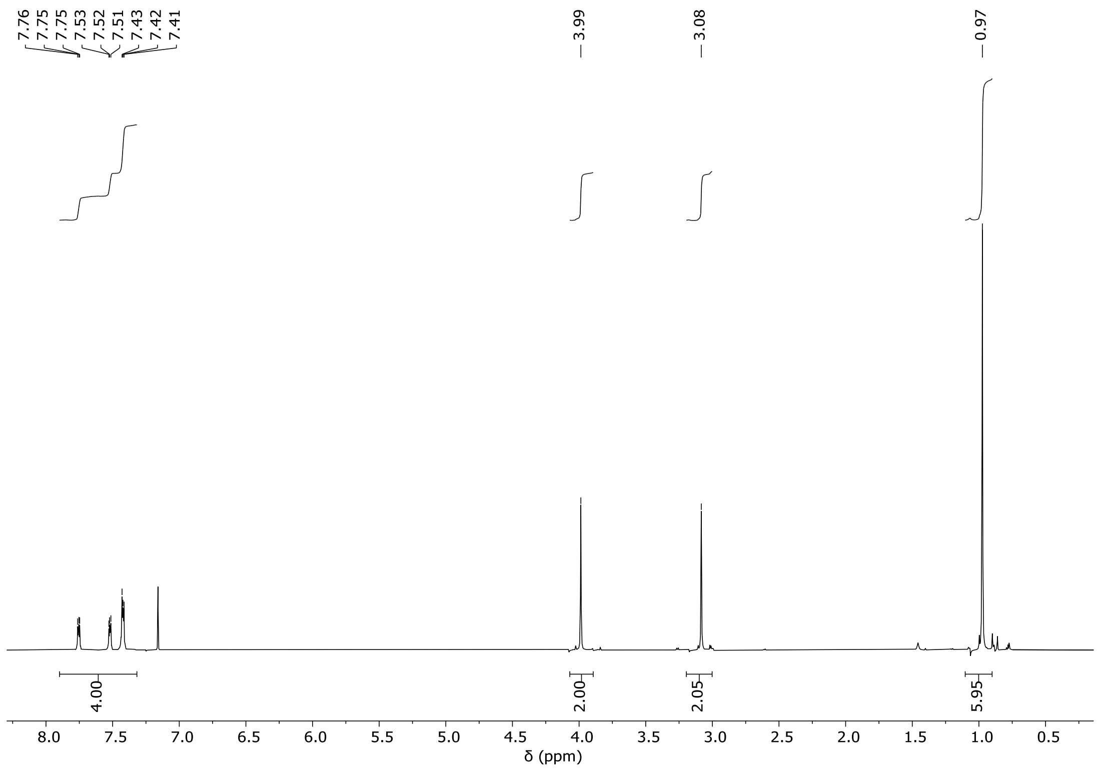

Figure S 72: ^{1}H NMR spectrum (400 MHz, $CDCl_3$, 25°C) of the isolated polymer corresponding to table 2, run #1 showing a selective poly(ester-*alt*-thioester) formation.

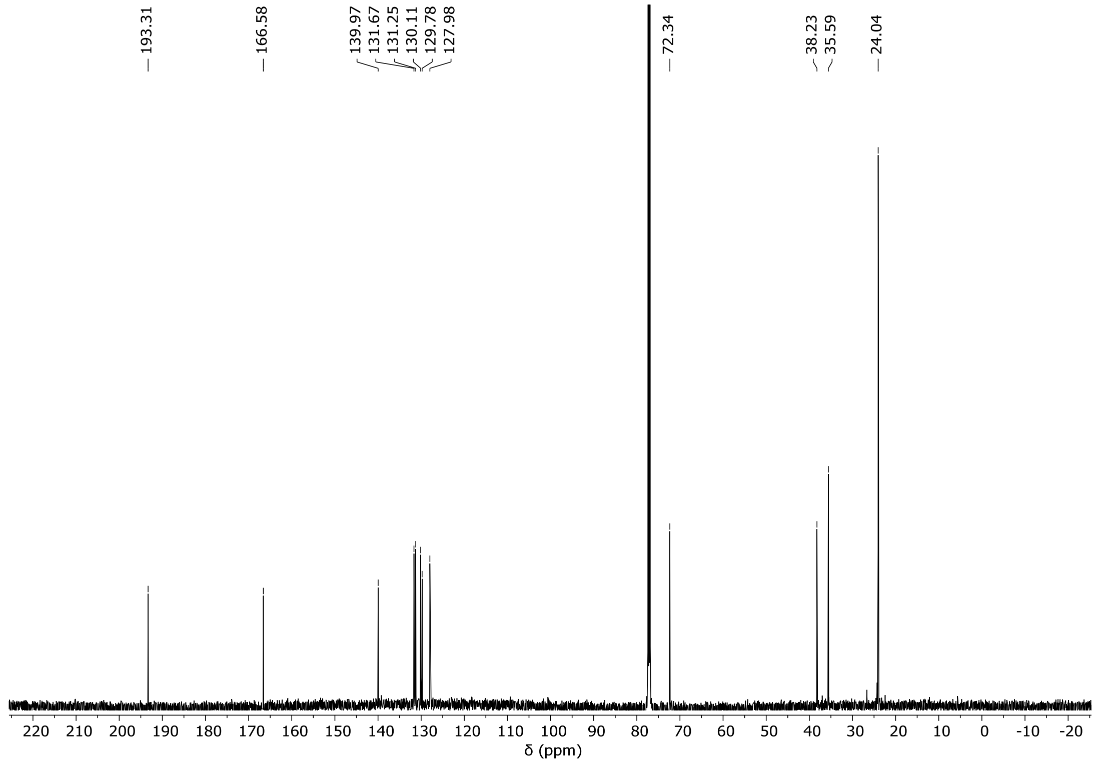

Figure S 73: ^{13}C NMR spectrum (126 MHz, $CDCl_3$, 25°C) of the isolated polymer corresponding to table 2, run #1.

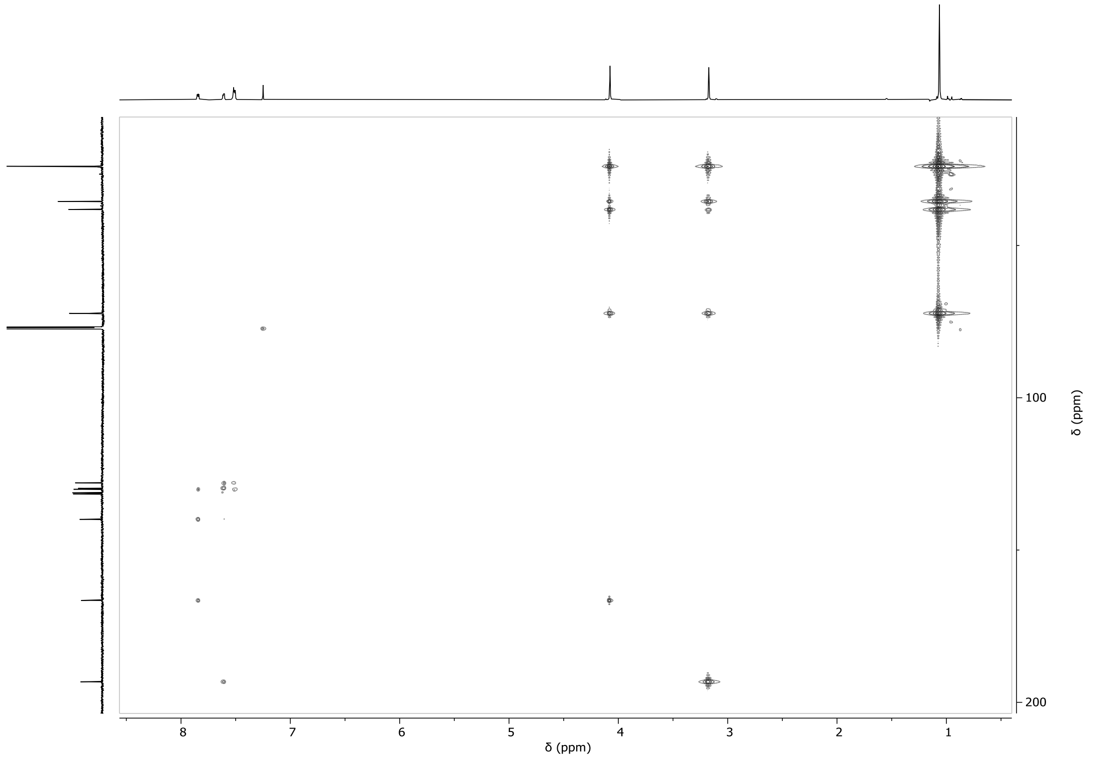

Figure S 74: ^1H-^{13}C HMBC NMR spectrum ($CDCl_3$, 25°C) spectrum of the isolated polymer corresponding to table 2, run #1.

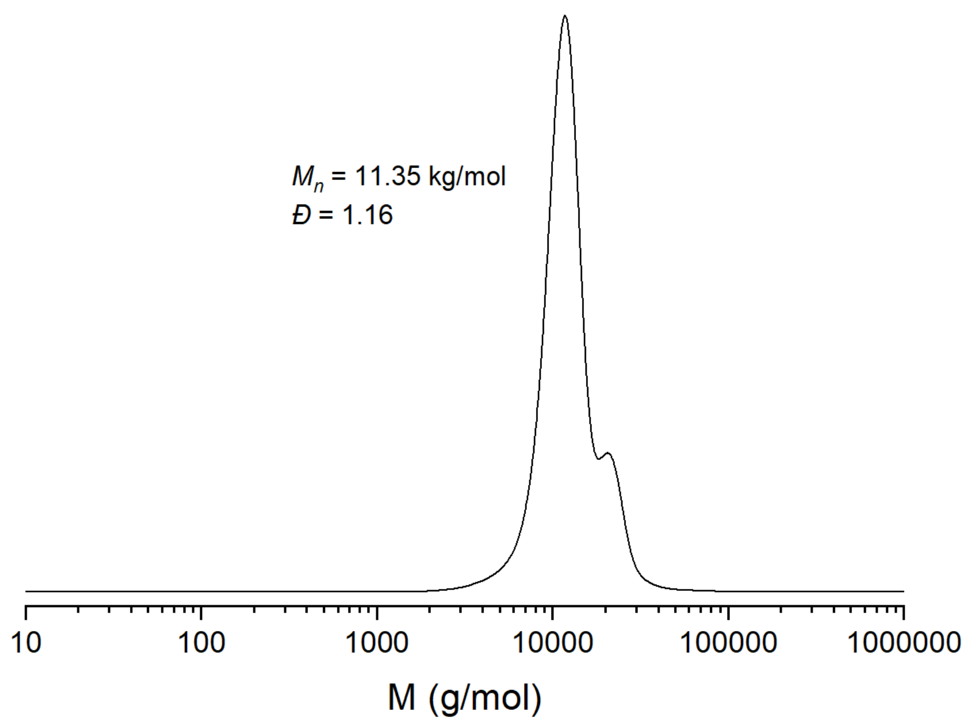

Figure S 75: SEC traces corresponding to table 2, run #1.

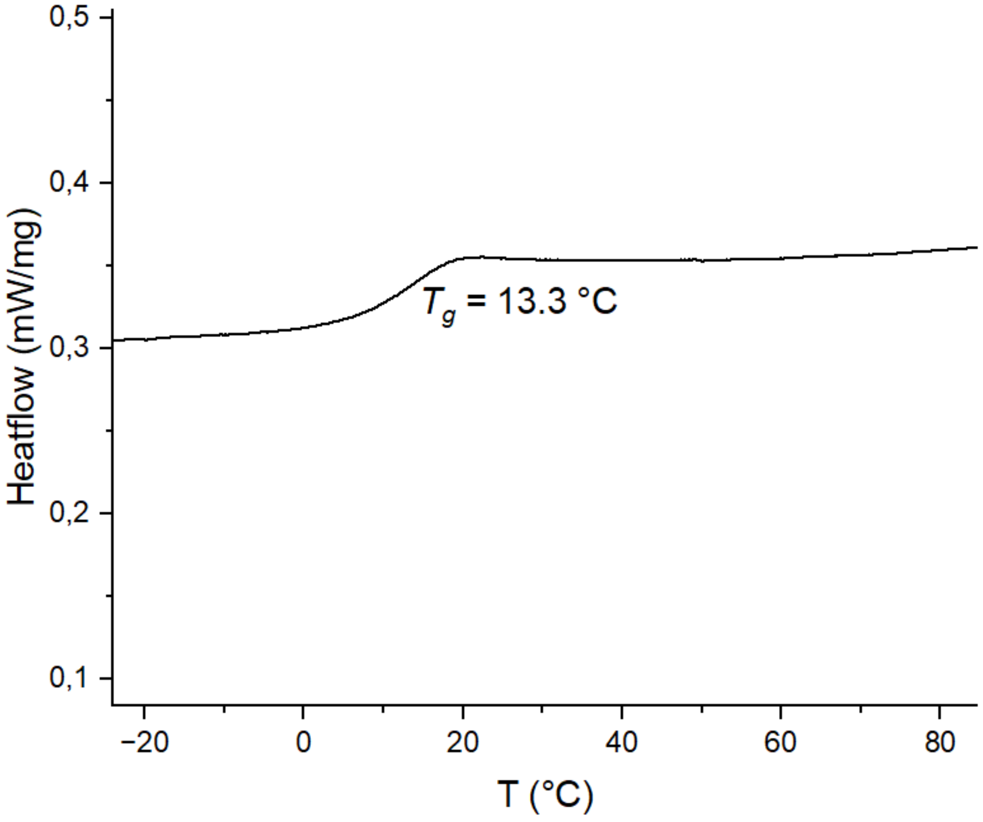

Figure S 76: DSC data of copolymer corresponding to table 2, run #1.

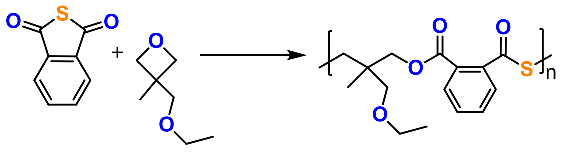

Scheme 3: Synthesis of CS_2/OX^{OEt} copolymer.

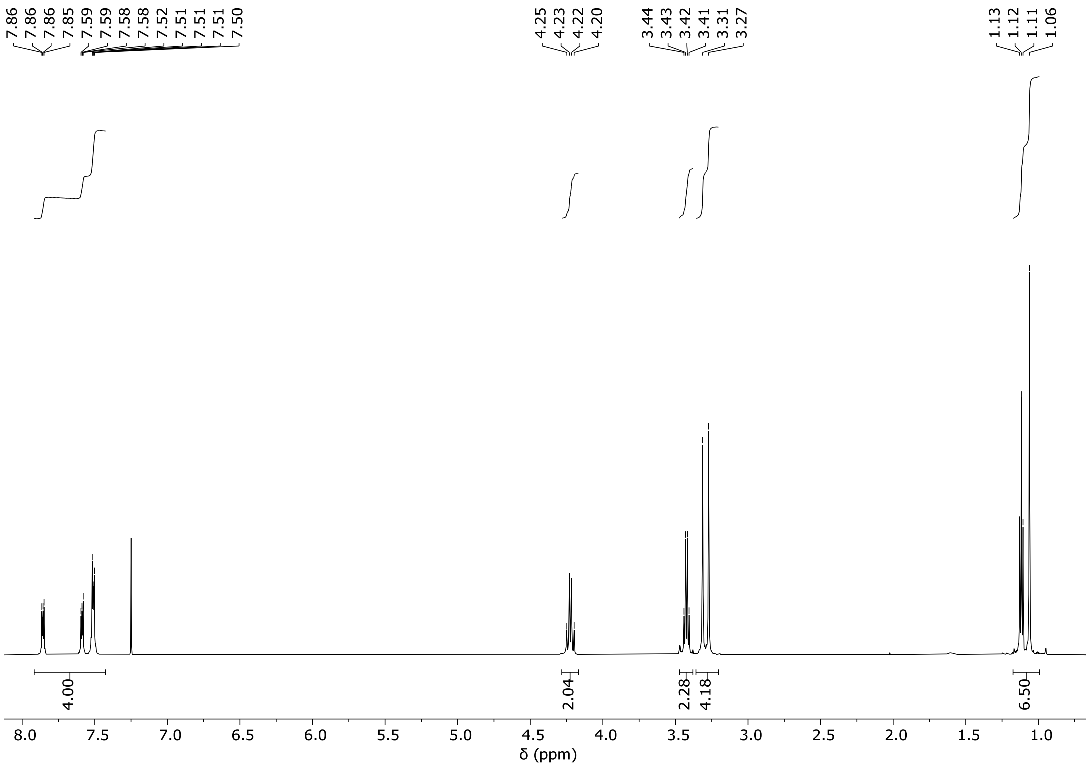

Figure S 77: ^{1}H NMR spectrum (400 MHz, $CDCl_3$, 25°C) of the isolated polymer corresponding to table 2, run #2 showing a selective poly(ester-*alt*-thioester) formation.

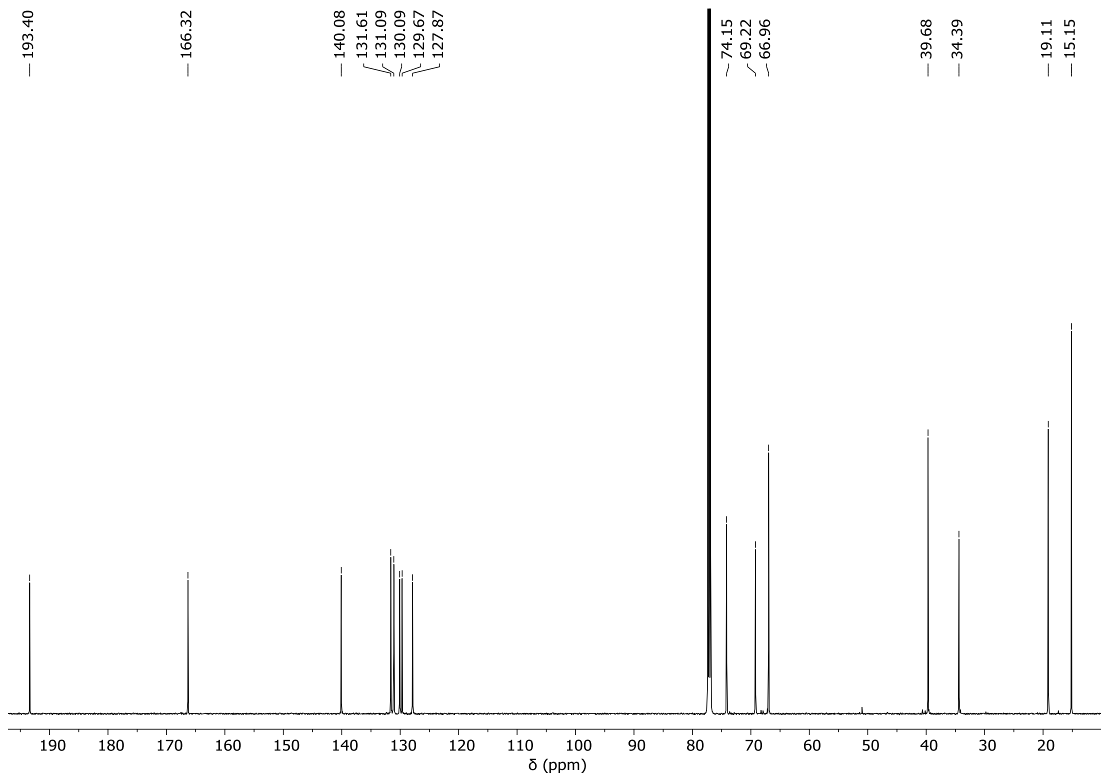

Figure S 78: ^{13}C NMR spectrum (126 MHz, $CDCl_3$, 25°C) of the isolated polymer corresponding to table 2, run #2.

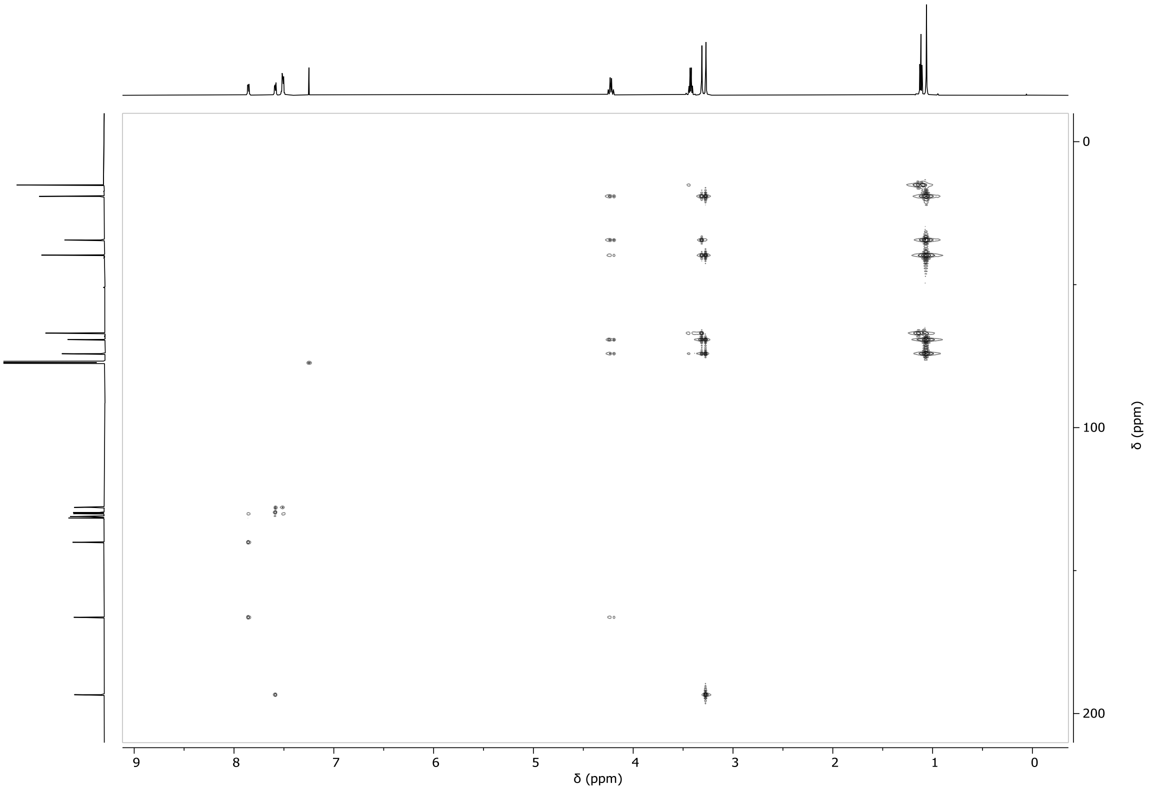

Figure S 79: ^{1}H-^{13}C HMBC NMR spectrum ($CDCl_3$, 25°C) spectrum of the isolated polymer corresponding to table 2, run #2.

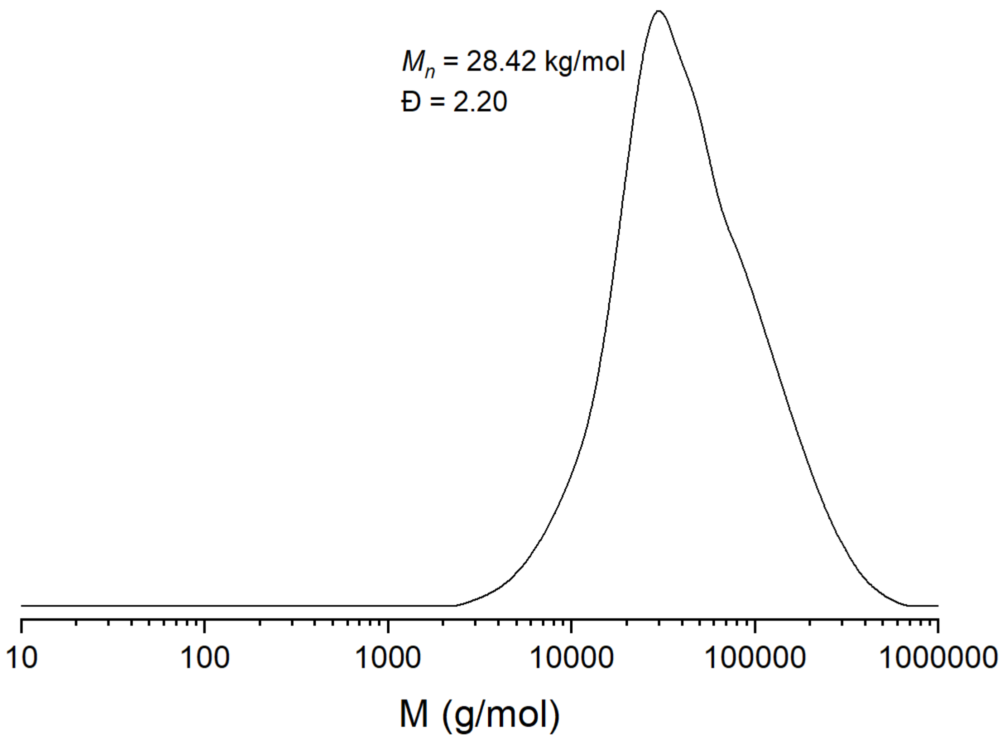

Figure S 80: SEC traces corresponding to table 2, run #2.

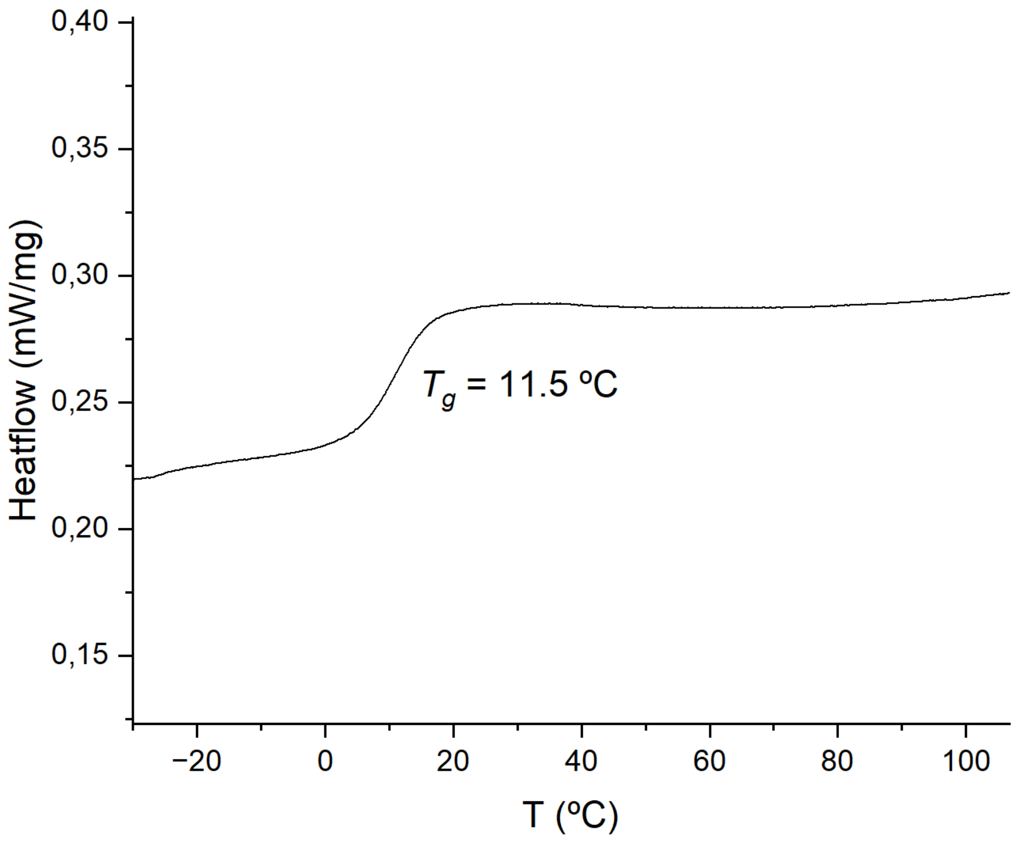

Figure S 81: DSC data of copolymer corresponding to table 2, run #2.

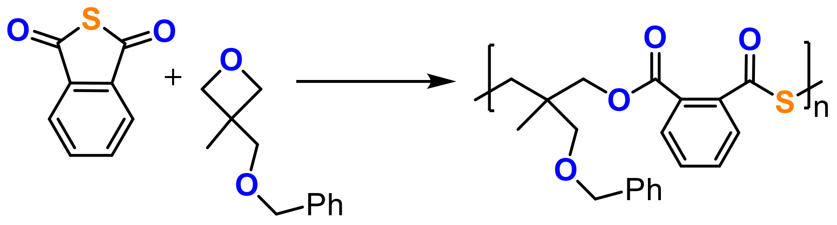

Scheme 4: Synthesis of PTA/OX^{OBn} copolymer.

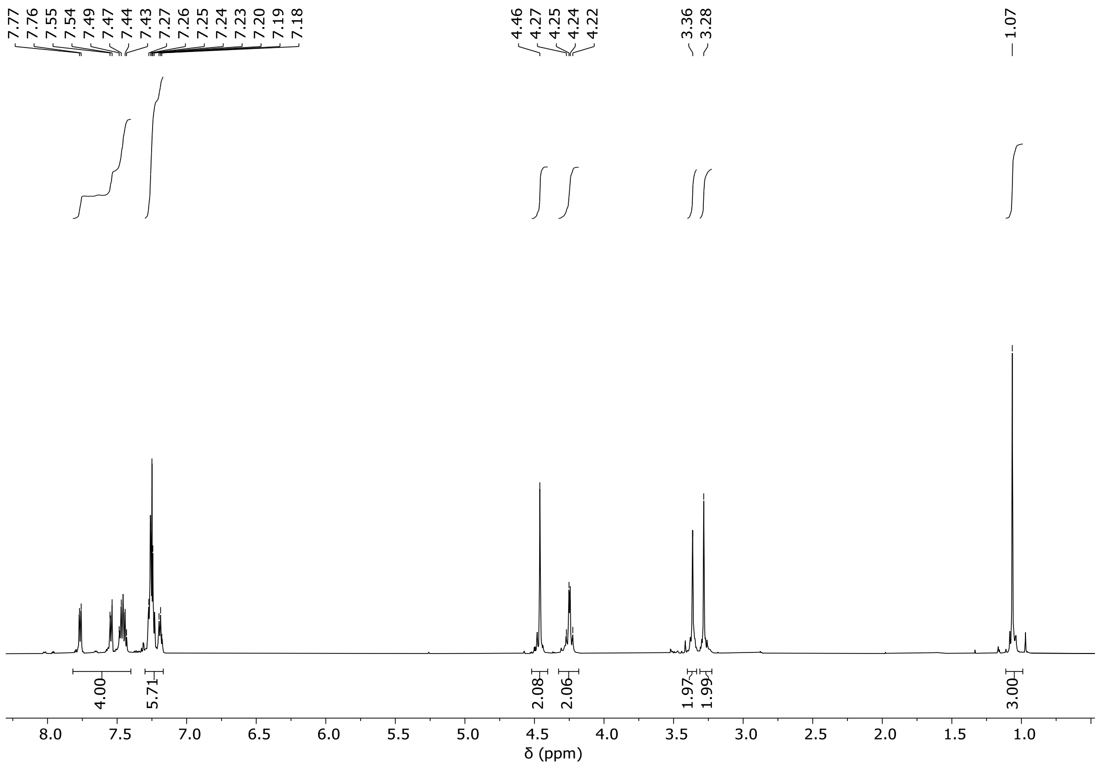

Figure S 82: ^{1}H NMR spectrum (400 MHz, $CDCl_3$, 25°C) of the isolated polymer corresponding to table 2, run #3 showing a selective poly(ester-*alt*-thioester) formation.

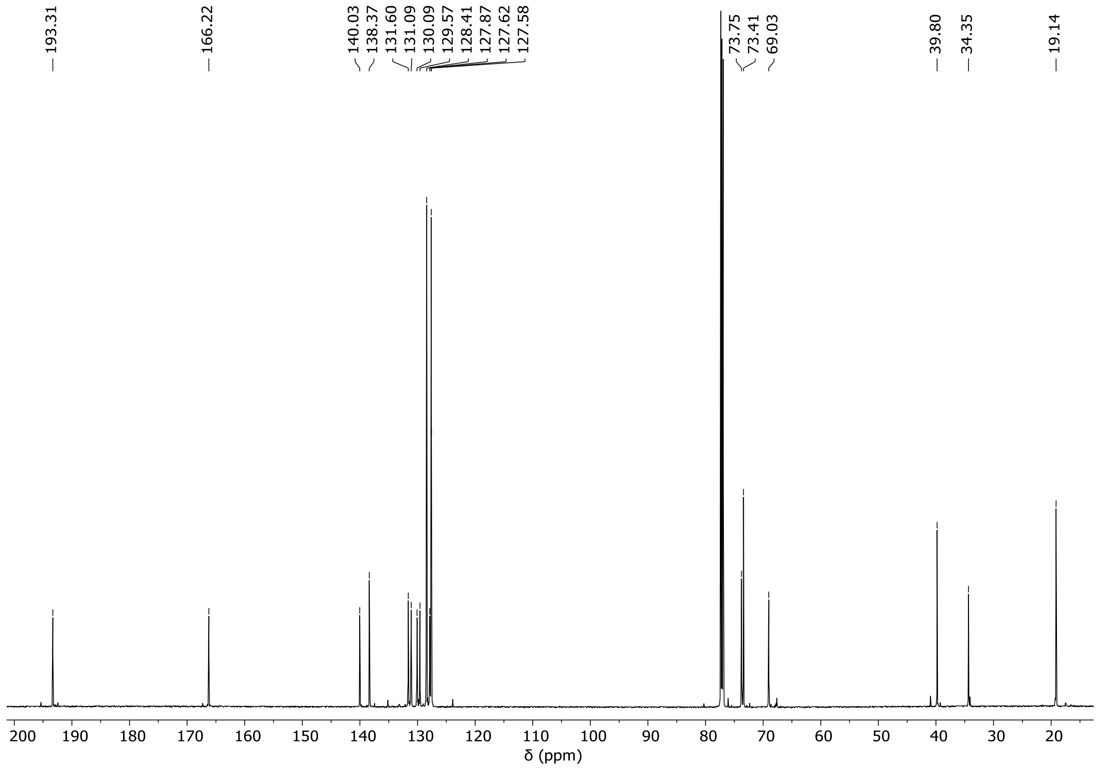

Figure S 83: ^{13}C NMR spectrum (126 MHz, $CDCl_3$, 25°C) of the isolated polymer corresponding to table 2, run #3.

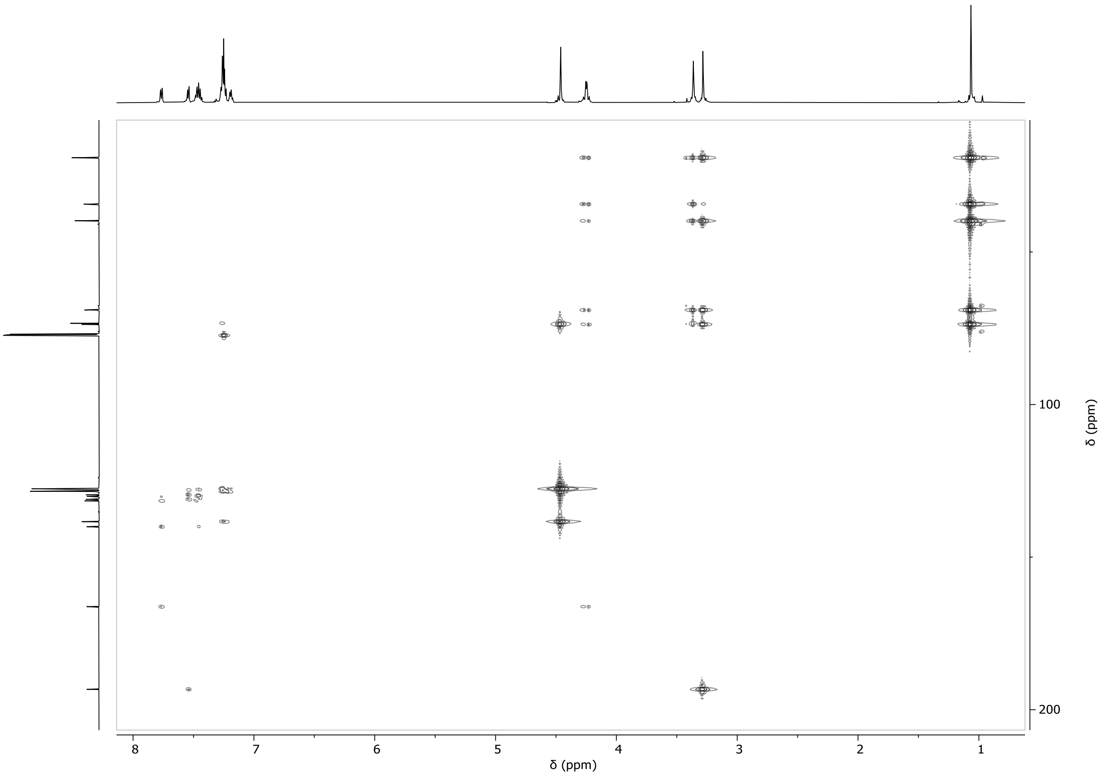

Figure S 84: ^{1}H-^{13}C HMBC NMR spectrum ($CDCl_3$, 25°C) spectrum of the isolated polymer corresponding to table 2, run #3.

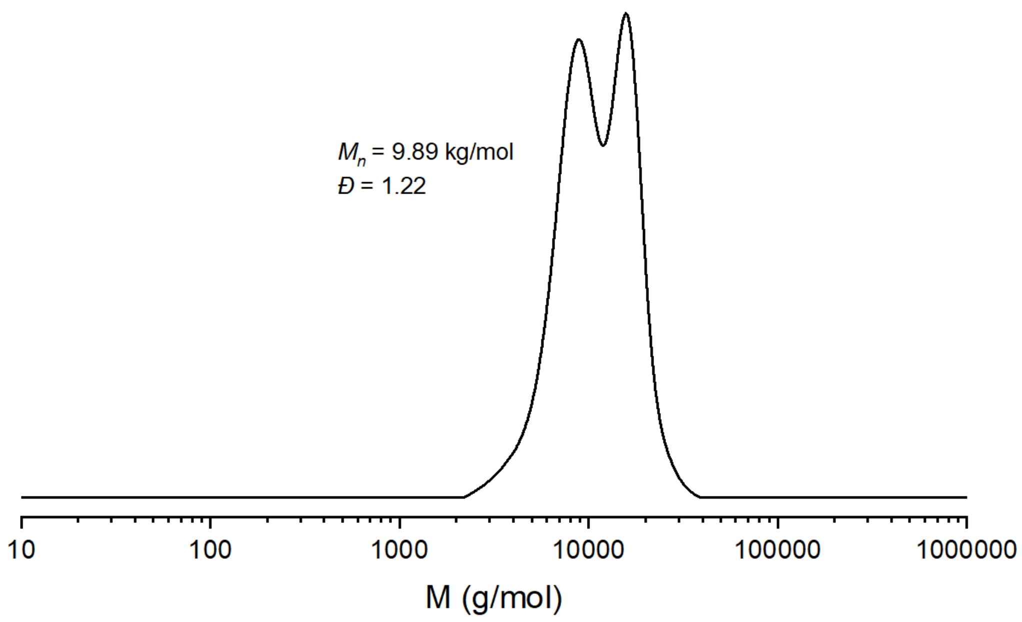

Figure S 85: SEC traces corresponding to table 2, run #3.

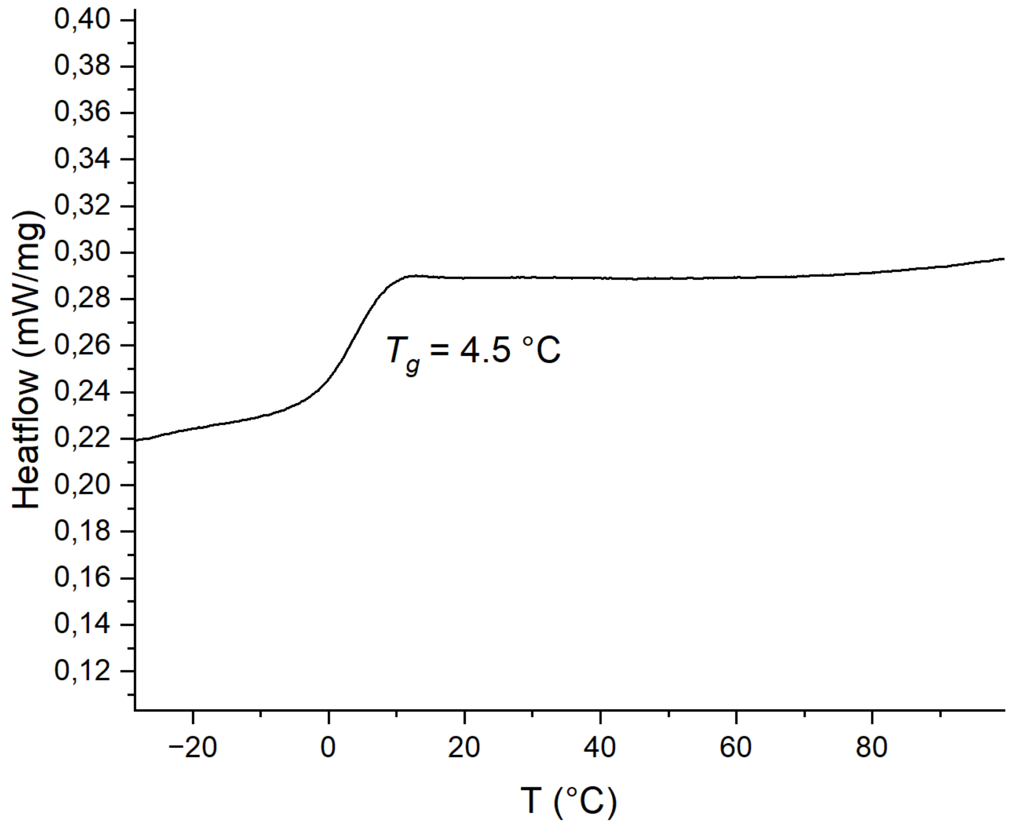

Figure S 86: DSC data of copolymer corresponding to table 2, run #3.

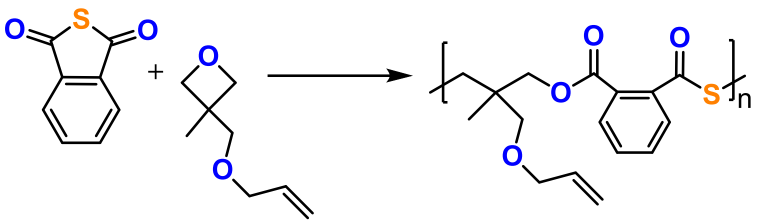

Scheme 5: Synthesis of PTA/OX^{OAllyl} copolymer.

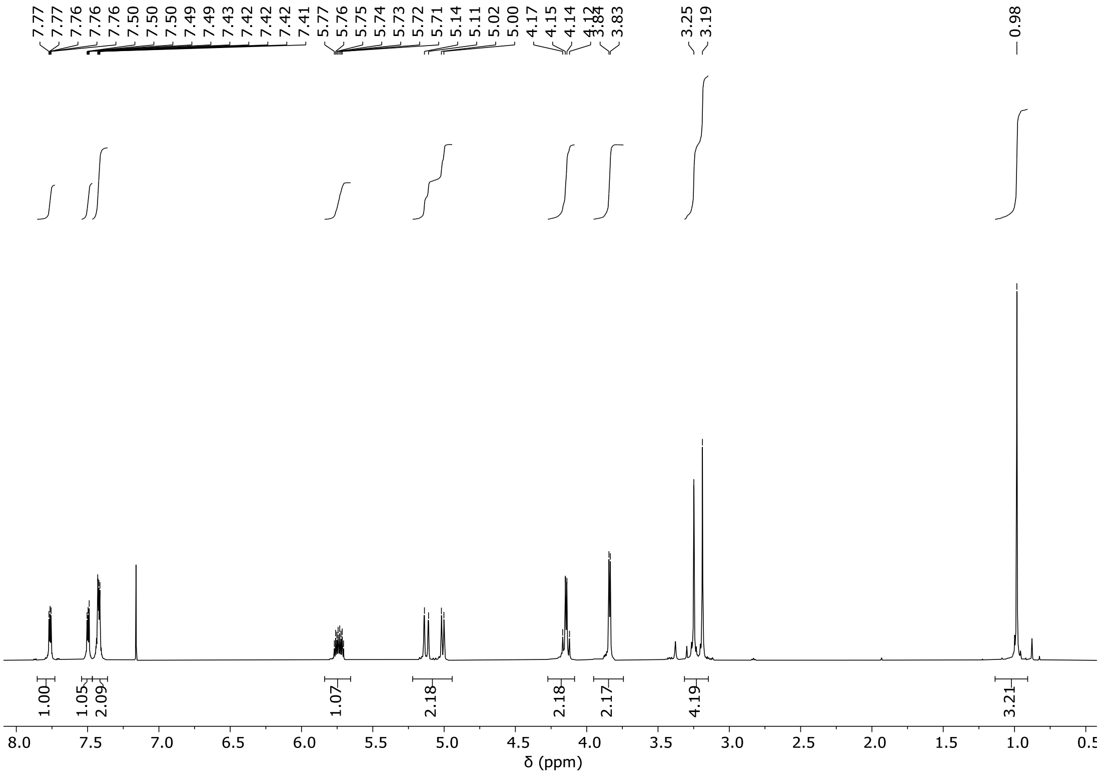

Figure S 87: ^{1}H NMR spectrum (400 MHz, $CDCl_3$, 25°C) of the isolated polymer corresponding to table 2, run #4 showing a selective poly(ester-*alt*-thioester) formation.

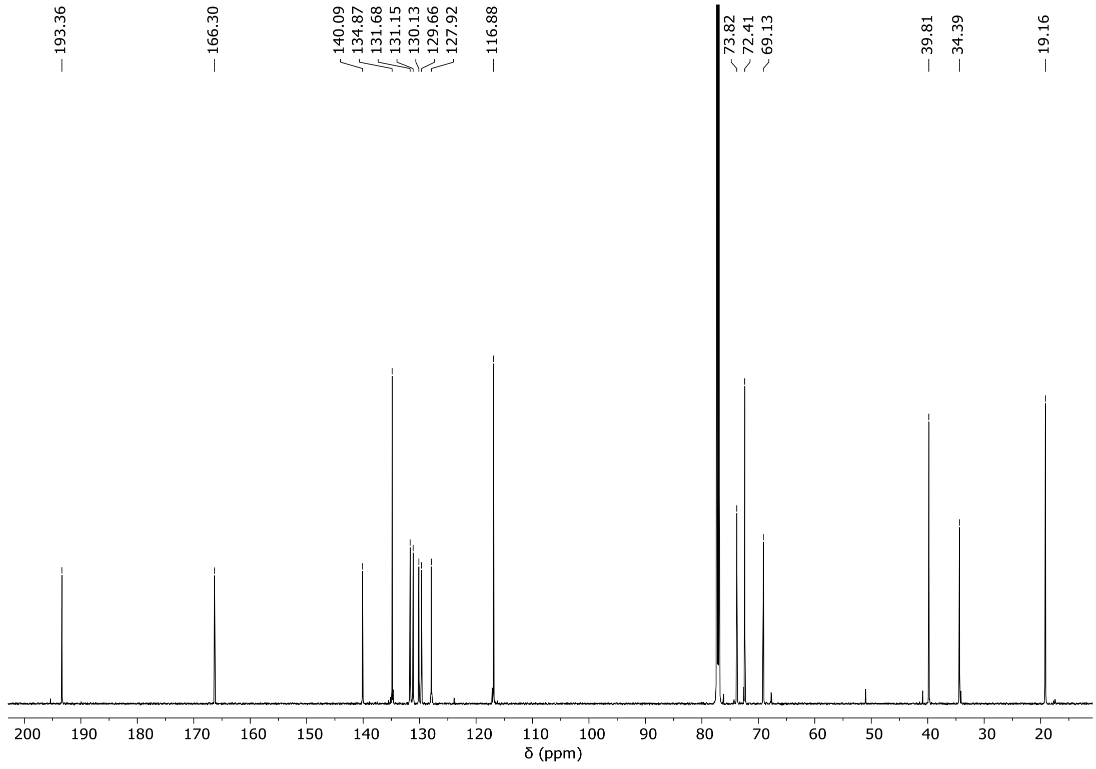

Figure S 88: ^{13}C NMR spectrum (126 MHz, $CDCl_3$, 25°C) of the isolated polymer corresponding to table 2, run #4.

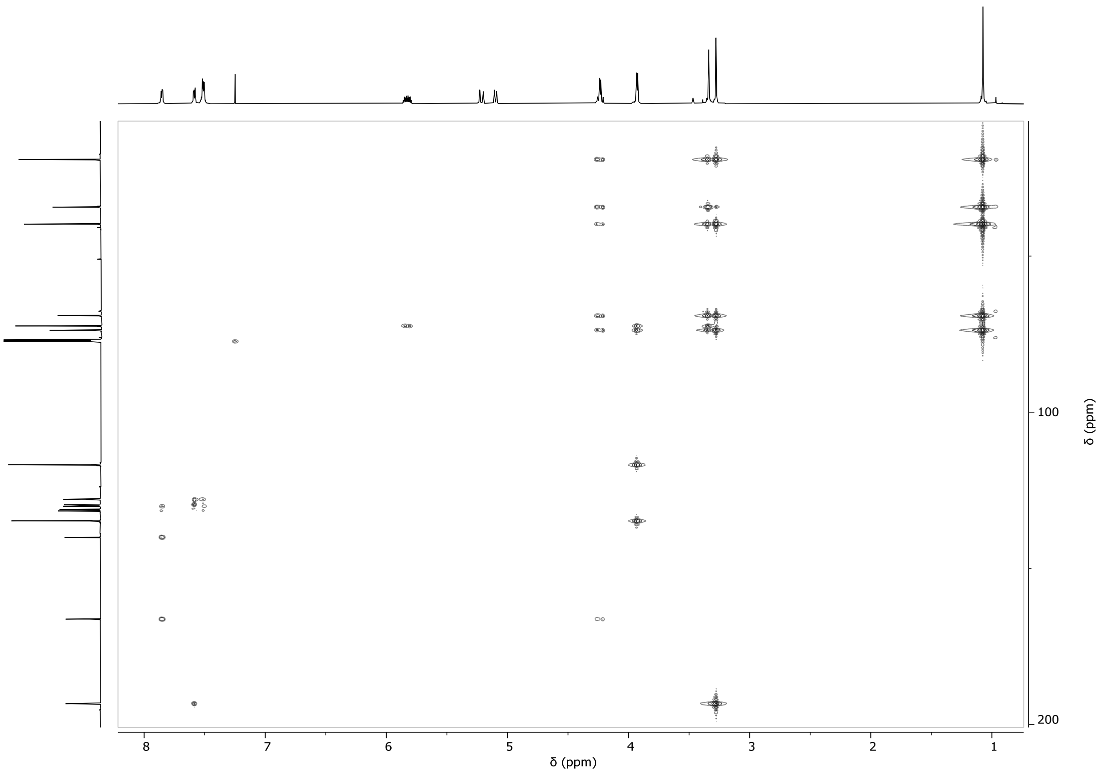

Figure S 89: ^{1}H-^{13}C HMBC NMR spectrum ($CDCl_3$, 25°C) spectrum of the isolated polymer corresponding to table 2, run #4.

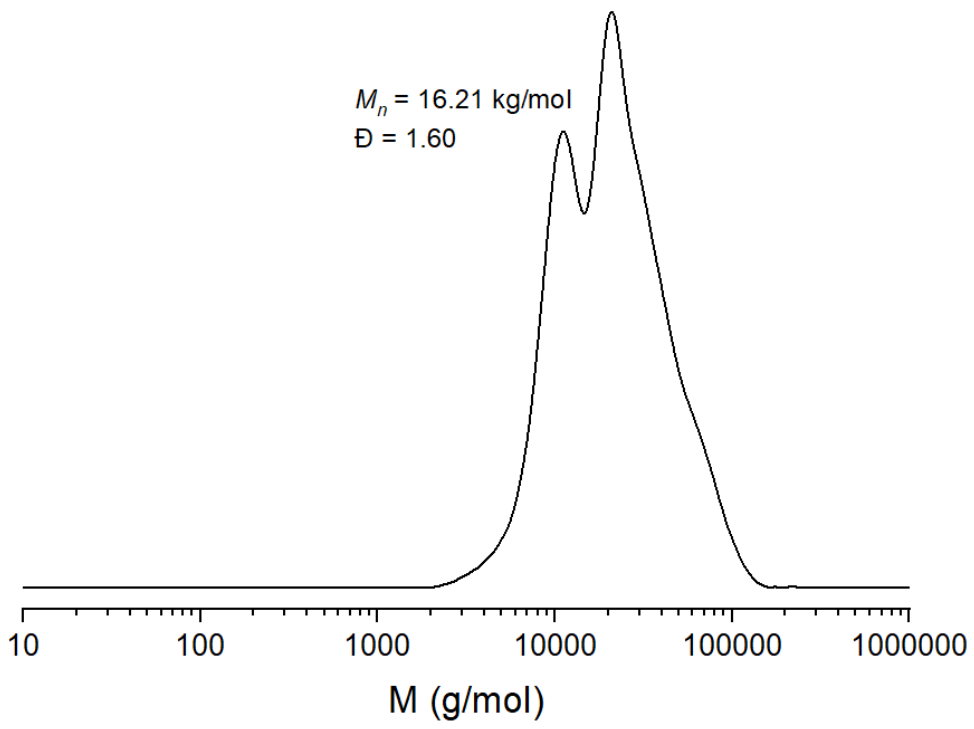

Figure S 90: SEC traces corresponding to table 2, run #4.

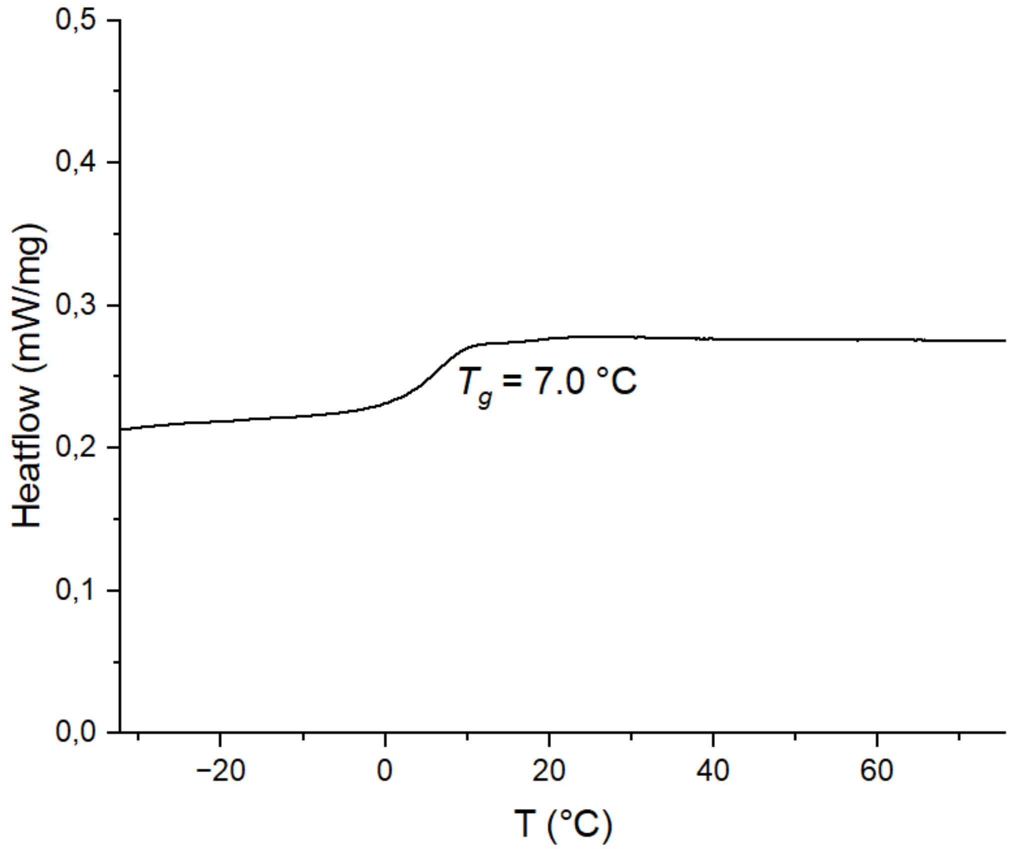

Figure S 91: DSC data of copolymer corresponding to table 2, run #4.

Section S6: DFT Studies

Table S 2: Calculated Gibbs free energies and ΔΔG.

R =	TS_{intra} (ii)	Int_{intra} (ii)	Int_{inter} (i)	ΔΔG
H	+108.1	+67.0	+57.3	–9.7
Me	+126.6	+80.1	+84,6	+4.5
$-C_3H_6-$	+149.7	+70.2	+80.0	+9.8

Gaussian16[3] Revision B.01 was used to perform DFT calculations with the B97D3 functional.[4] Geometry optimizations were performed with the def2-SVP basis set.[5,6] Frequency calculations of optimized structures were performed at the same level of theory (def2-SVP) to characterize the structures to be minima (no imaginary frequency) or transition states (one imaginary frequency) and yield thermodynamic corrections. Single point energy corrections were calculated at the B97D3 / def2-TZVPP level of theory. The bulky solvation effect of THF was simulated by SMD[7] continuum solvent mode at the B97D3 / def2-TZVPP level of theory. xyz-coordinates are available as separate xyz-files. It should be noted that all intermediates and transition states modelled in the computational investigations were calculated in the quartet ($^4Cr^{3+}$) spin-state.

Section S7: Epoxide/CS_2 Copolymerisations

Table S 3: LCrK catalysed CS_2/epoxide ROCOP with different epoxides.

Run	Epox.	t [h]	Conv. [%]^a	Polymer selectivity [%]^b
#0*	OX	20	99	>99
#1	EO	16	0	-
#2	PO	16	48	54
#3	DMO	41	44	0
#4*	CHO	8	49	76
#5	CPO	136	37	>99
#6	SO	24	55	37
#7	EGE	22	43	60
#8	FPO	24	86	0

ROCOP conducted at 50ºC with 1 eq. LCrK: 1000 eq. Epoxide: 2000 eq. CS_2. ^{a} CHO conversion calculated by comparing the relative integrals in the normalised ^1H NMR spectrum ($CDCl_3$, 400 MHz) of aliphatic resonances due to polymer versus unconsumed CHO. ^{b} Relative integrals in the normalised ^1H NMR spectrum ($CDCl_3$, 400 MHz) of aliphatic resonances due to polymer versus cyclic byproduct (dt-c5c). *adapted from ref. 1 (#0, T = 60°C) and ref. 8 (#4, T = 60°C), respectively.

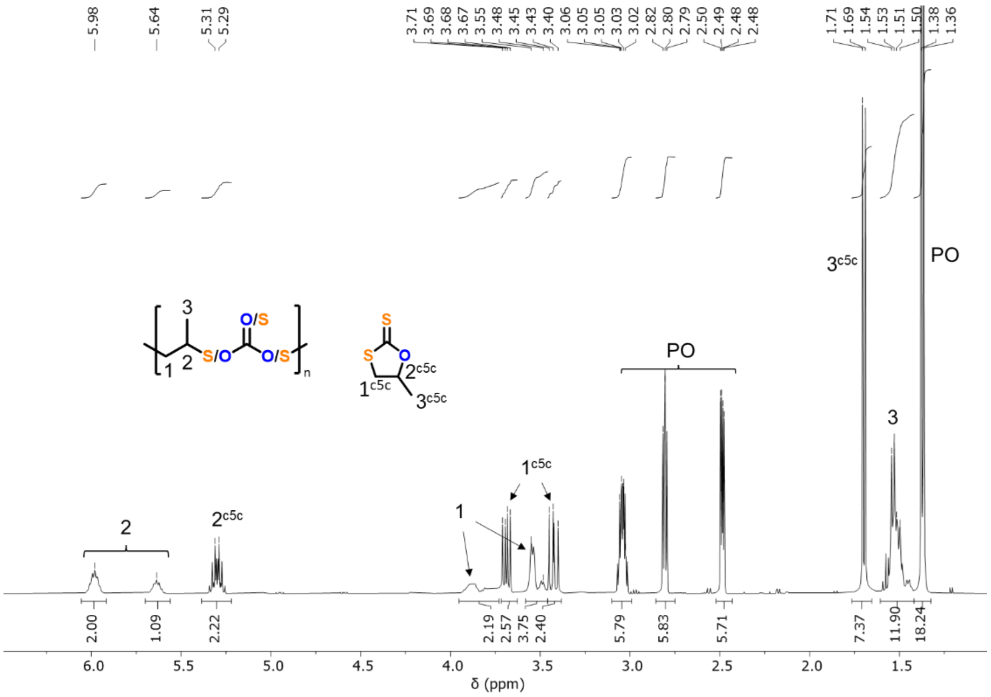

Figure S 92: ^{1}H NMR spectrum (400 MHz, $CDCl_3$, 25°C) of the crude reaction mixture corresponding to table S 2, run #2 showing a polymer selectivity of 54%.

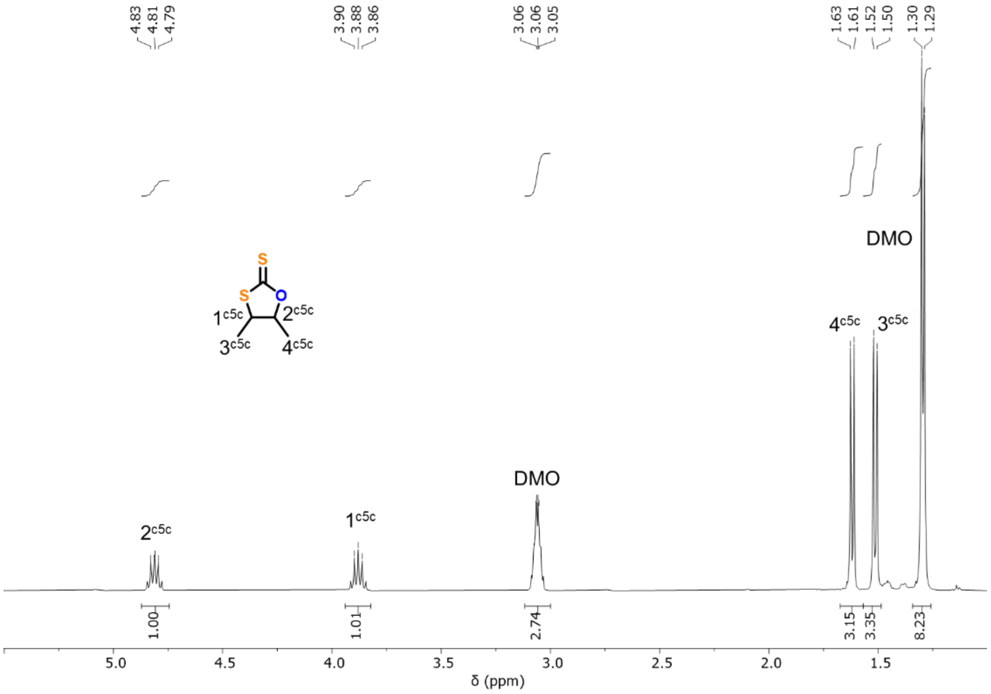

Figure S 93: ^{1}H NMR spectrum (400 MHz, $CDCl_3$, 25°C) of the crude reaction mixture corresponding to table S 2, run #3 showing only the formation of cyclic biproduct.

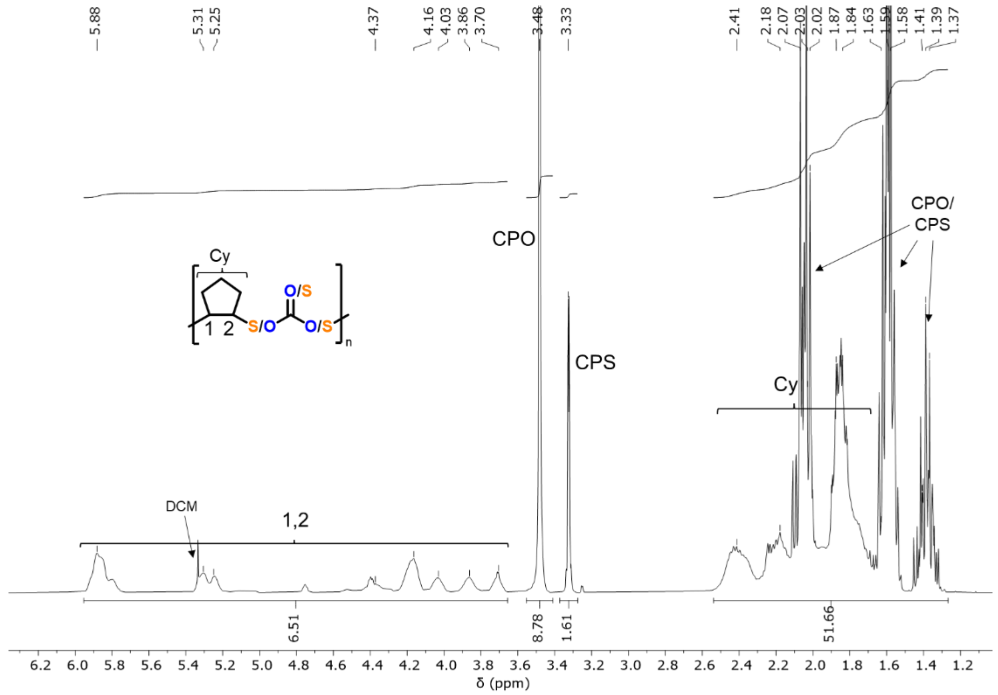

Figure S 94: ^{1}H NMR spectrum (400 MHz, $CDCl_3$, 25°C) of the crude reaction mixture corresponding to table S 2, run #5 showing a polymer selectivity of >99%. Significant formation of cyclopentane sulfide (CPS) is evident.

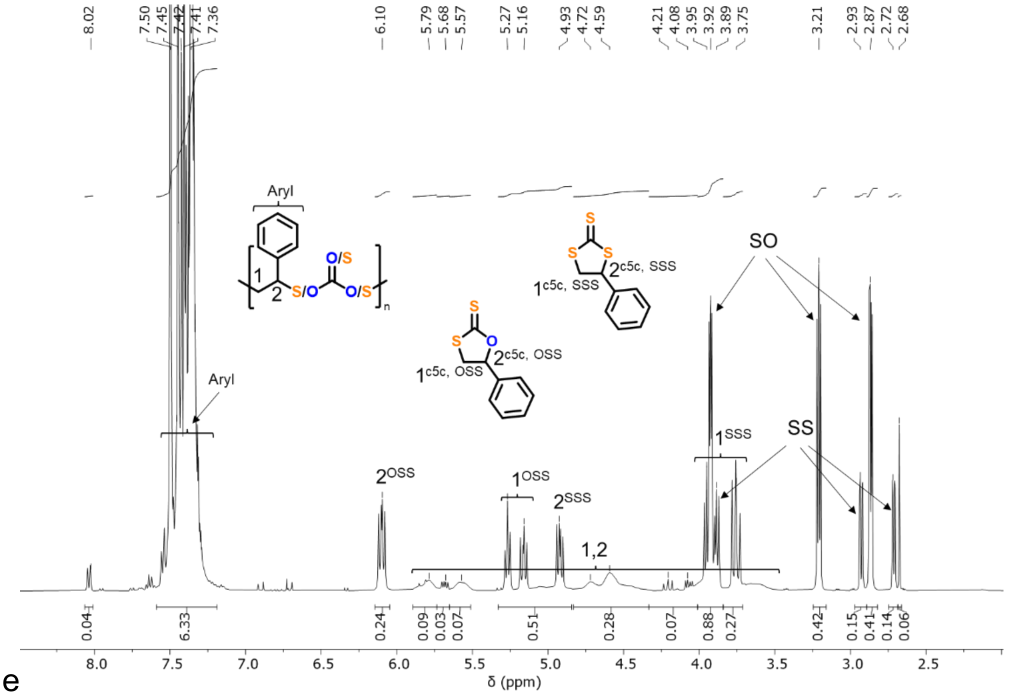

e

Figure S 95: ^{1}H NMR spectrum (400 MHz, $CDCl_3$, 25°C) of the crude reaction mixture corresponding to table S 2, run #6 showing a polymer selectivity of 37%. Significant formation of styrene sulfide (SS) is evident.

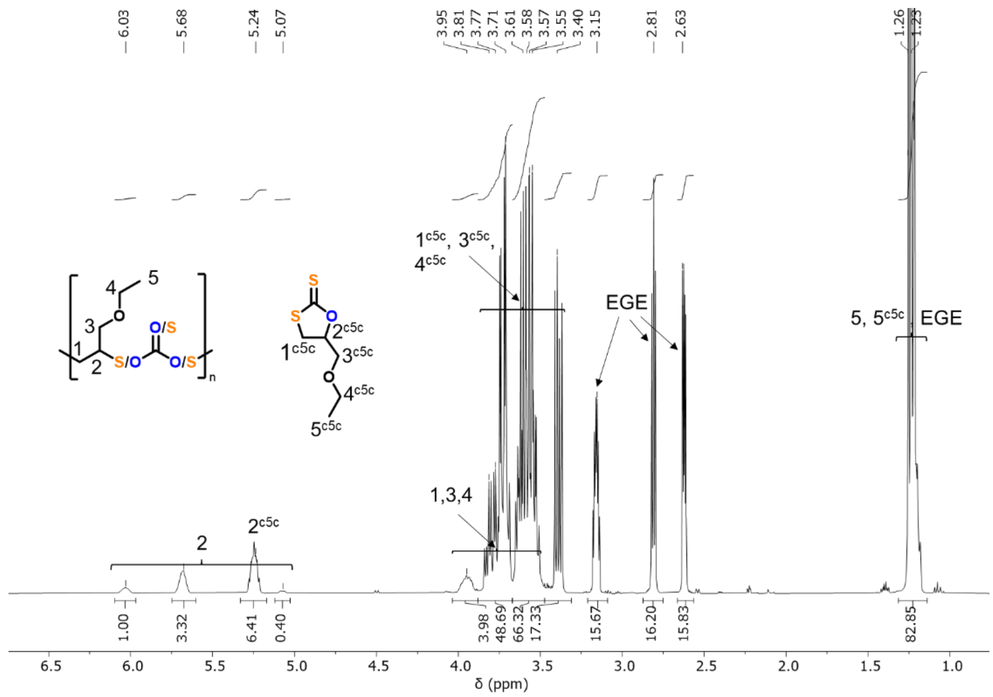

Figure S 96: ^{1}H NMR spectrum (400 MHz, $CDCl_3$, 25°C) of the crude reaction mixture corresponding to table S 2, run #7 showing a polymer selectivity of 60%.

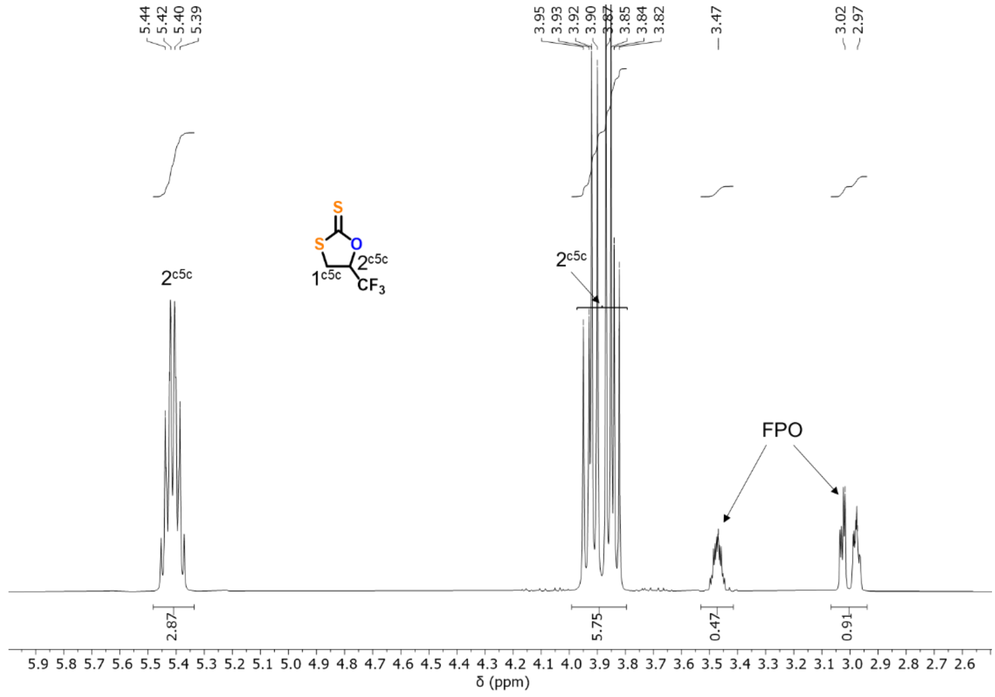

Figure S 97: ^{1}H NMR spectrum (400 MHz, $CDCl_3$, 25°C) of the crude reaction mixture corresponding to table S 2, run #8 showing only the formation of cyclic biproduct.

Section S8: Exceeding Reaction Times for (PO,OX)/(PTA,CS_2) Copolymerisations

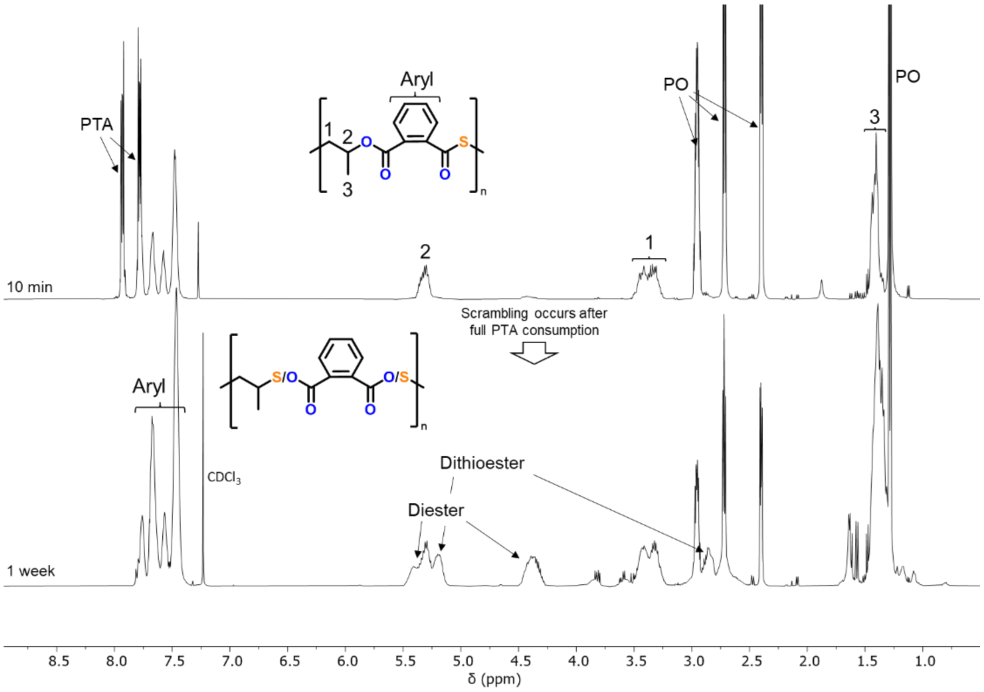

Figure S 98: Overlaid ^{1}H NMR spectra (400 MHz, $CDCl_3$, 25°C) of the aliquots taken after 10 min and 1 week of the PO/PTA ROCOP using the bicomponent metal catalyst Cat 1. It becomes evident that exceeding the reaction time leads to the formation of scrambled di(thio)ester linkages.

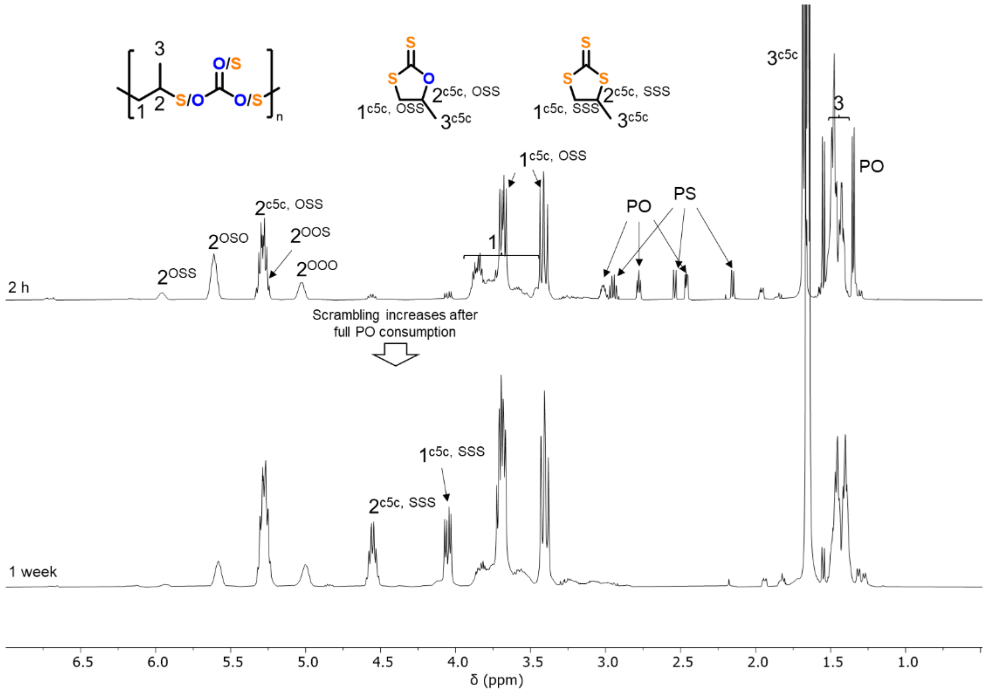

Figure S 99: Overlaid ^{1}H NMR spectra (400 MHz, $CDCl_3$, 25°C) of the aliquots taken after 2 h and 1 week of the PO/CS_2 ROCOP using the bicomponent metal catalyst Cat 1. It becomes evident that exceeding the reaction time leads to an increased formation of cyclic di- and trithiocarbonates while the relative amount of oxygen rich polymer linkages increases.

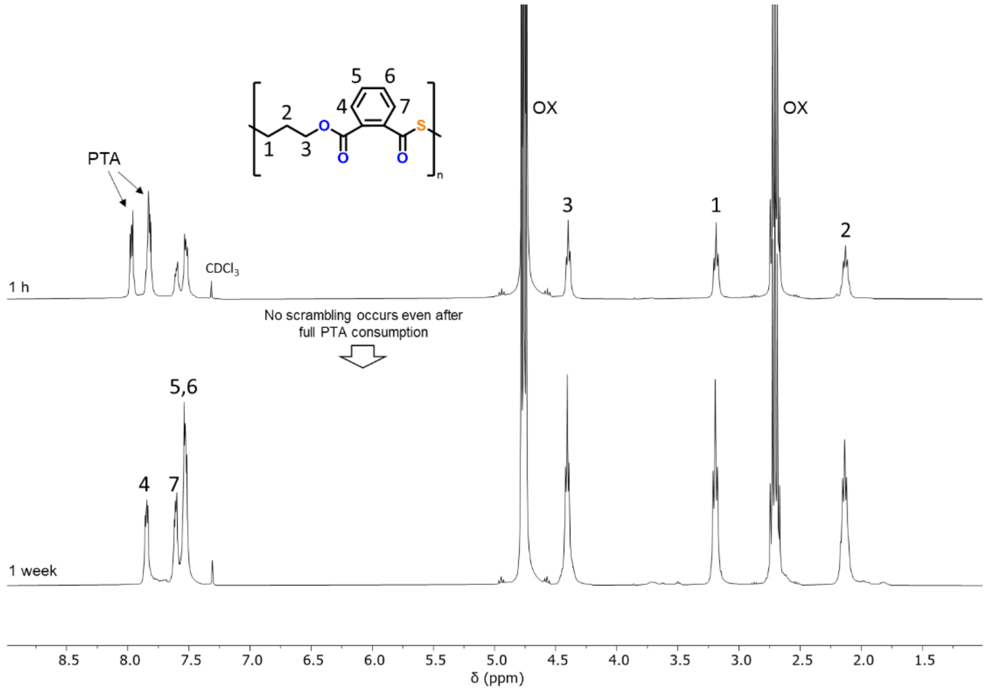

Figure S 100: Overlaid ^{1}H NMR spectra (400 MHz, $CDCl_3$, 25°C) of the aliquots taken after 1 h and 1 week of the OX/PTA ROCOP using the bicomponent metal catalyst Cat 1. No scrambling is observed even after exceeding the reaction time.

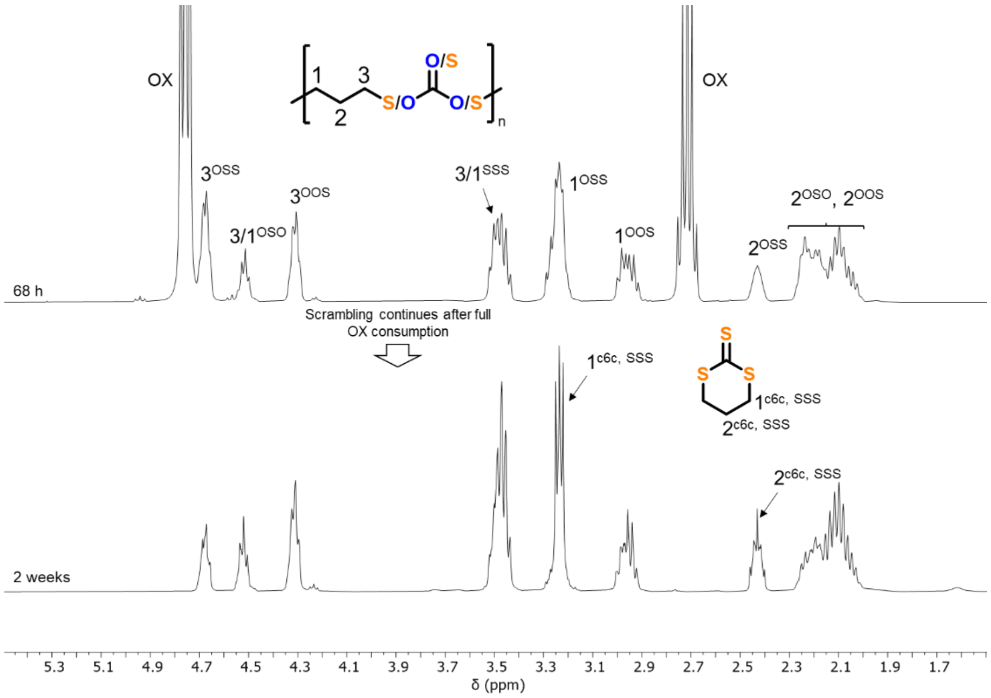

Figure S 101: Overlaid ^{1}H NMR spectra (400 MHz, $CDCl_3$, 25°C) of the aliquots taken after 68 h and 2 weeks of the OX/CS_2 ROCOP using the bicomponent metal catalyst Cat 1. It becomes evident that exceeding the reaction time leads to the formation of cyclic trithiocarbonate while the relative amount of oxygen rich polymer linkages increases.

Section S9: Crystallography

X-Ray data were collected on a BRUKER D8 Venture system. Data were collected at 100(2) K using graphite-monochromated Mo K_{α} radiation (λ_{α} = 0.71073 Å). The strategy for the data collection was evaluated by using the Smart software. The data were collected by the standard "*ψ-ω* scan techniques" and were scaled and reduced using Saint+software. The structures were solved by using Olex2,[9] the structure was solved with the XT[10] structure solution program using Intrinsic Phasing and refined with the XL refinement package[11,12] using Least Squares minimization. If it is noted, bond length and angles were measured with Diamond Crystal and Molecular Structure Visualization Version 4.6.2.[13] Drawings were generated with POV-Ray.[14]

Table S 4. Crystallographic data.

**Empirical formula**	$C_{11}H_{10}O_2S_2$
**Formula weight**	238.31
**Temperature/K**	100
**Crystal system**	triclinic
**Space group**	P-1
**a/Å**	8.5111(3)
**b/Å**	11.2673(4)
**c/Å**	12.0154(5)
**α/°**	90.902(2)
**β/°**	99.837(2)
**γ/°**	110.120(2)
**Volume/Å^3**	1062.56(7)
**Z**	4
**ρ_{calc}g/cm^3**	1.490
**μ/mm^{-1}**	0.475
**F(000)**	496.0
**Crystal size/mm^3**	0.351 × 0.165 × 0.137
**Crystal shape**	Block
**Crystal color**	Plate
**Radiation**	MoKα (λ = 0.71073)
**2Θ range for data collection/°**	3.862 to 52.786
**Index ranges**	-10 ≤ h ≤ 10, -14 ≤ k ≤ 14, -15 ≤ l ≤ 15
**Reflections collected**	34242
**Independent reflections**	4349 [R_{int} = 0.0655, R_{sigma} = 0.0331]
**Data/restraints/parameters**	4349/0/273
**Goodness-of-fit on F^2**	1.114
**Final R indexes [I>=2σ (I)]**	R_1 = 0.0381, wR_2 = 0.0812
**Final R indexes [all data]**	R_1 = 0.0609, wR_2 = 0.0999
**Largest diff. peak/hole / e Å^{-3}**	0.81/-0.34

Section S10: Bibliography

[1] C. Fronacon-Wood, B. R. Manjunathe, M. R. Stühler, C. Gallizioli, C. Müller, P. Pröhm, A.J.Plajer, *Nat. Commun.* **2023**, *14*, 4525.

[2] L.-Y. Wang, G.-G. Gu, T.-J. Yue, W.-M. Ren, X.-B. Lu, *Macromol.* **2019**, *52*, 2439-2445.

[3] M. J. Frisch, G. W. Trucks, H. B. Schlegel, G. E. Scuseria, M. A. Robb, J. R. Cheeseman, G. Scalmani, V. Barone, G. A. Petersson, H. Nakatsuji, et al., **2016**.

[4] S. Grimme, S. Ehrlich, L. Goerigk, *J. Comput. Chem.* **2011**, *32*, 1456–1465.

[5] F. Weigend, C. Hättig, H. Patzelt, R. Ahlrichs, S. Spencer, A. Willets, *Phys. Chem. Chem. Phys.* **2006**, *8*, 1057.

[6] F. Weigend, R. Ahlrichs, K. A. Peterson, T. H. Dunning, R. M. Pitzer, A. Bergner, *Phys. Chem. Chem. Phys.* **2005**, *7*, 3297.

[7] A. V. Marenich, C. J. Cramer, D. G. Truhlar, *J. Phys. Chem. B* **2009**, *113*, 6378–6396.

[8] J. Stephan, M. R. Stühler, S. M. Rupf, S. Neale, A. J. Plajer, *Cell Rep. Phys. Sci.* **2023**, *4*.

[9] O. V. Dolomanov, L. J. Bourhis, R. J. Gildea, J. A. K. Howard, H. Puschmann, *J. Appl. Cryst.* **2009**, *42*, 339–341.

[10] G. M. Sheldrick, *Acta Cryst.* **2015**, *A71*, 3–8.

[11] G. M. Sheldrick, *SHELXL Version 2014/7, Program for Chrystal Structure Solution and Refinement*, Göttingen, Germany, **2014**.

[12] G. M. Sheldrick, *Acta Cryst.* **2008**, *A64*, 112–122.

[13] K. Brandenburg, “Diamond: Crystal and Molecular Structure Visualization,” can be found under http://www.crystalimpact.com/diamond, **2017**.

[14] Persistance of Version Pty. Ltd., **2004**, Retrieved from http://www.povray.org/download/.
